# Supplementary material for: Filamentation of the bacterial bi-functional alcohol/aldehyde dehydrogenase AdhE is essential for substrate channeling and enzymatic regulation
Source: Nat Commun. 2020 Mar 18;11:1426. doi: 10.1038/s41467-020-15214-y (PMC7080775; doi:10.1038/s41467-020-15214-y)
Supplement: Supplementary file 1 — Supplementary Information [file 41467_2020_15214_MOESM1_ESM.pdf]

Supplementary information

Filamentation of the bacterial bi-functional alcohol/aldehyde dehydrogenase AdhE is essential for substrate channeling and enzymatic regulation

Pony et al.

a

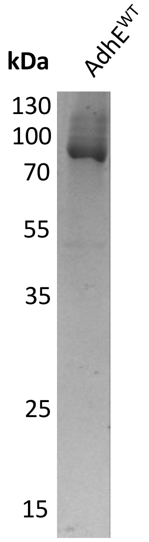

b

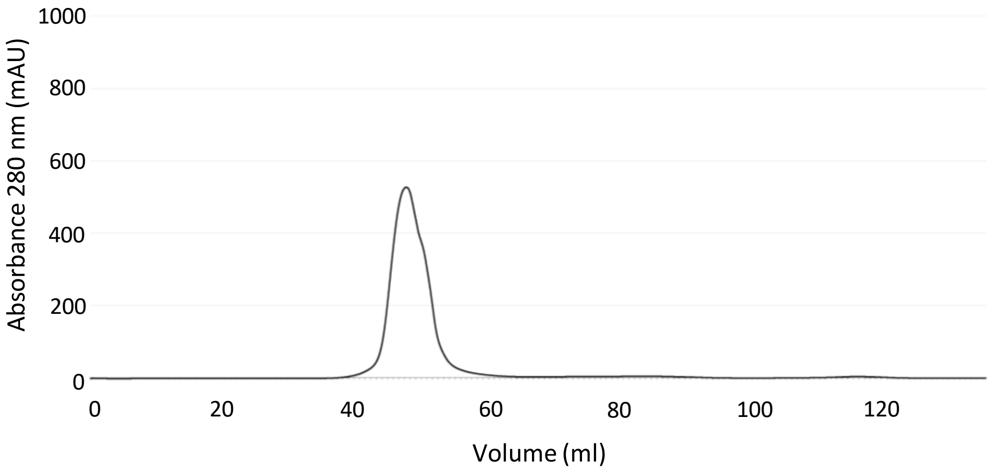

Supplementary Fig. 1. Purification of spirosomes. a. SDS-PAGE (12%) of purified AdhE^{WT}. Molecular weight are indicated on the left. **b.** Size-exclusion chromatography profile of purified AdhE^{WT} on a HiLoad 16/600 Superdex 200 pg column.

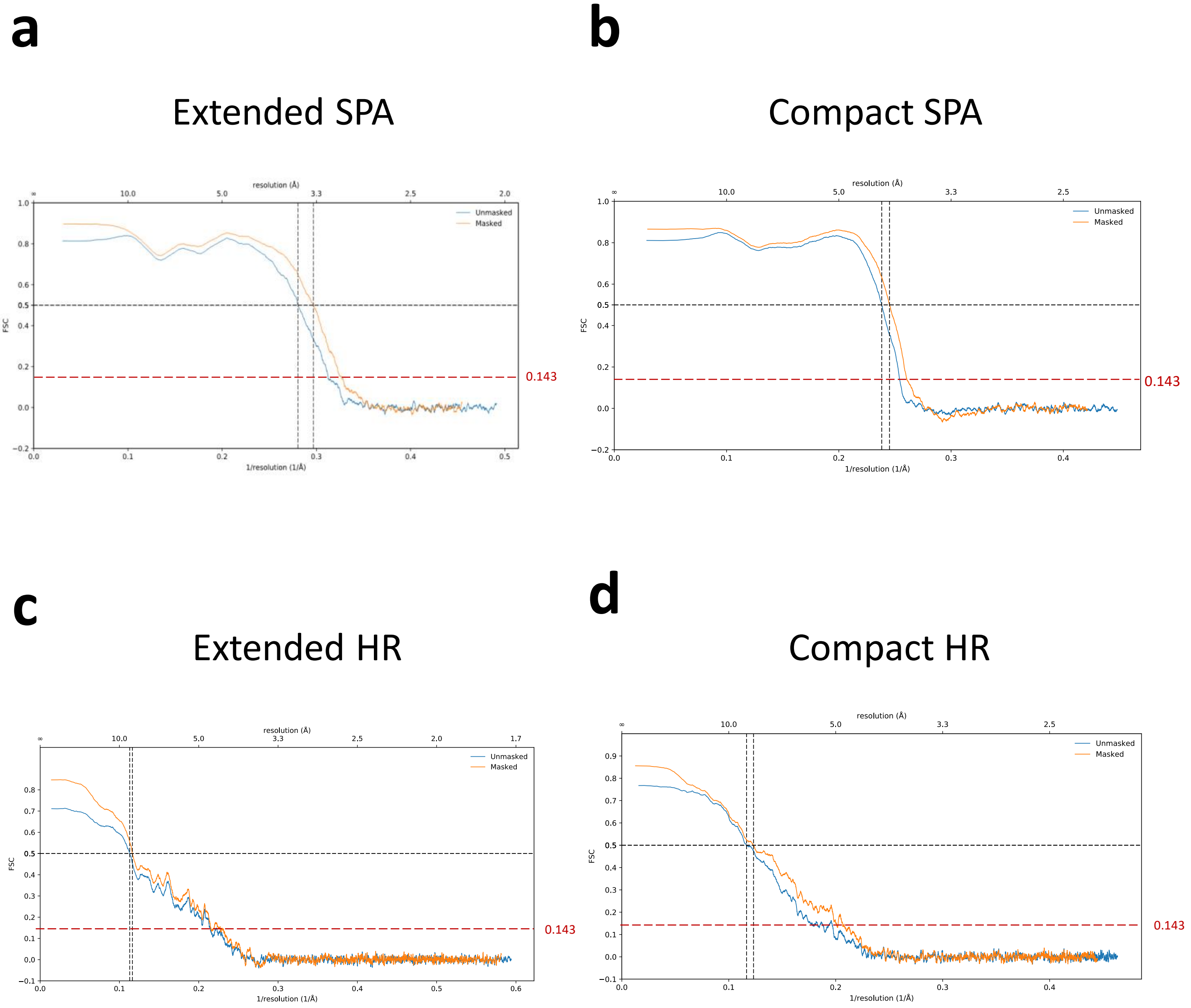

Supplementary Fig. 2. Resolution of the spirosome cryo-EM maps. Fourier shell correlation (FSC) curves for masked and unmasked reconstructions. **a.** extended spirosome obtained using SPA **b.** compact spirosome obtained using SPA **c.** extended spirosome obtained using HR **d.** compact spirosome obtained using HR. The resolution was calculated at the cut-off 0.143 of the FSC (red dashed line).

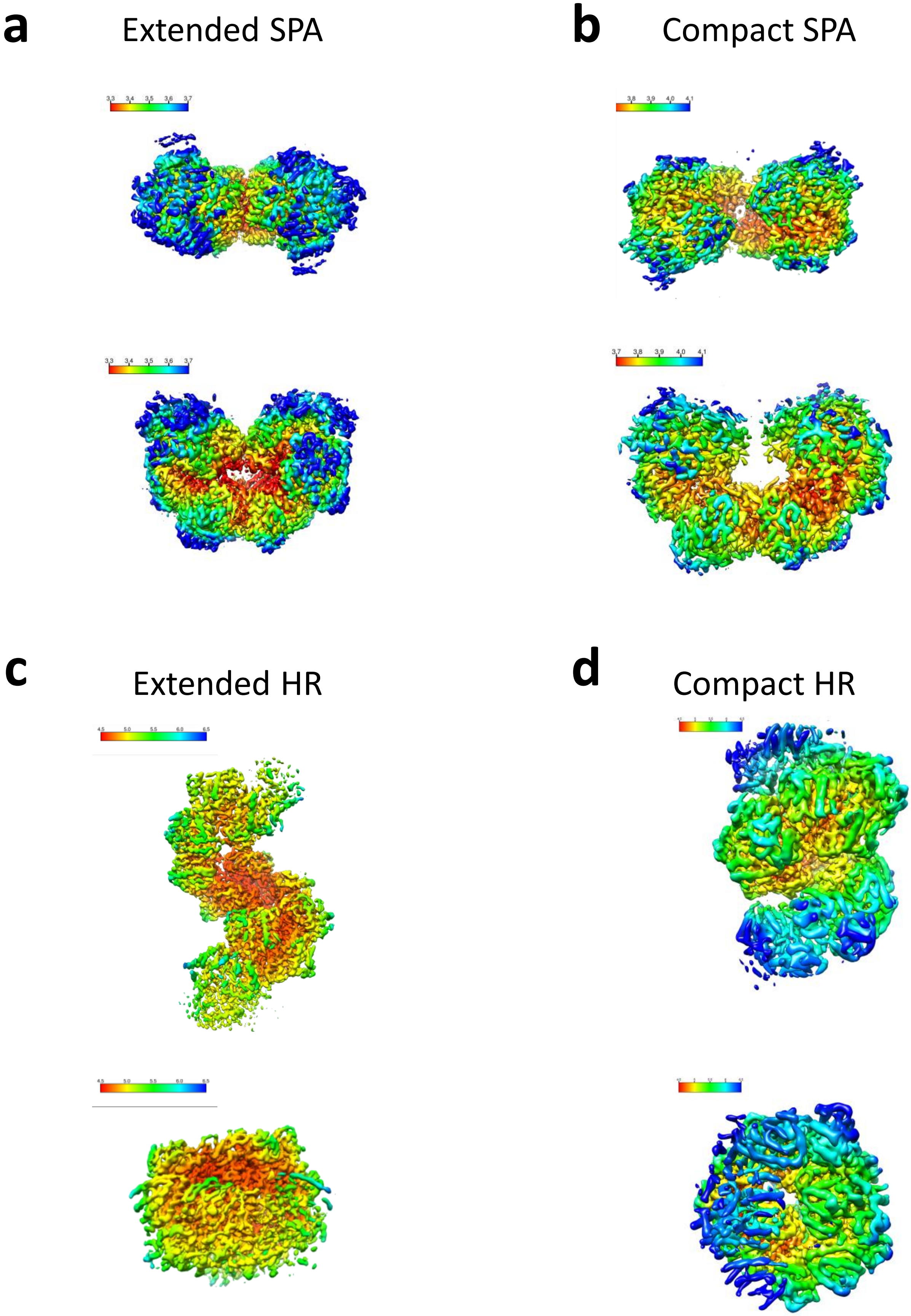

Supplementary Fig. 3. Representation of the local resolution in the cryo-EM maps . Local resolution is displayed on the cryoEM maps. A color-coded scale indicates the resolution values dispayed for each map **a.** extended spirosome (3.3Å (red) to 3.7Å (blue) resolution) obtained using SPA **b.** compact spirosome (3.7Å (red) to 4.1Å (blue) resolution) obtained using SPA. **c.** and **d.** extended spirosome and compact spirosome (4.5Å (red) to 6.5Å (blue) resolution) obtained using HR.

a

b

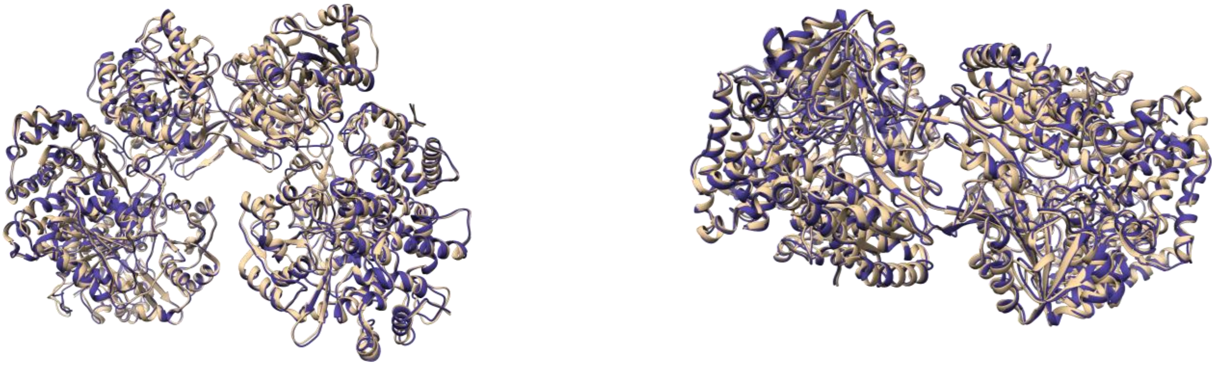

Supplementary Fig. 4. Comparison of apo and compact spirosome. Superimposition of the structures of the apo (PDB code: 6AHC) and the spirosomes in presence of NADH and Fe^{2+}.

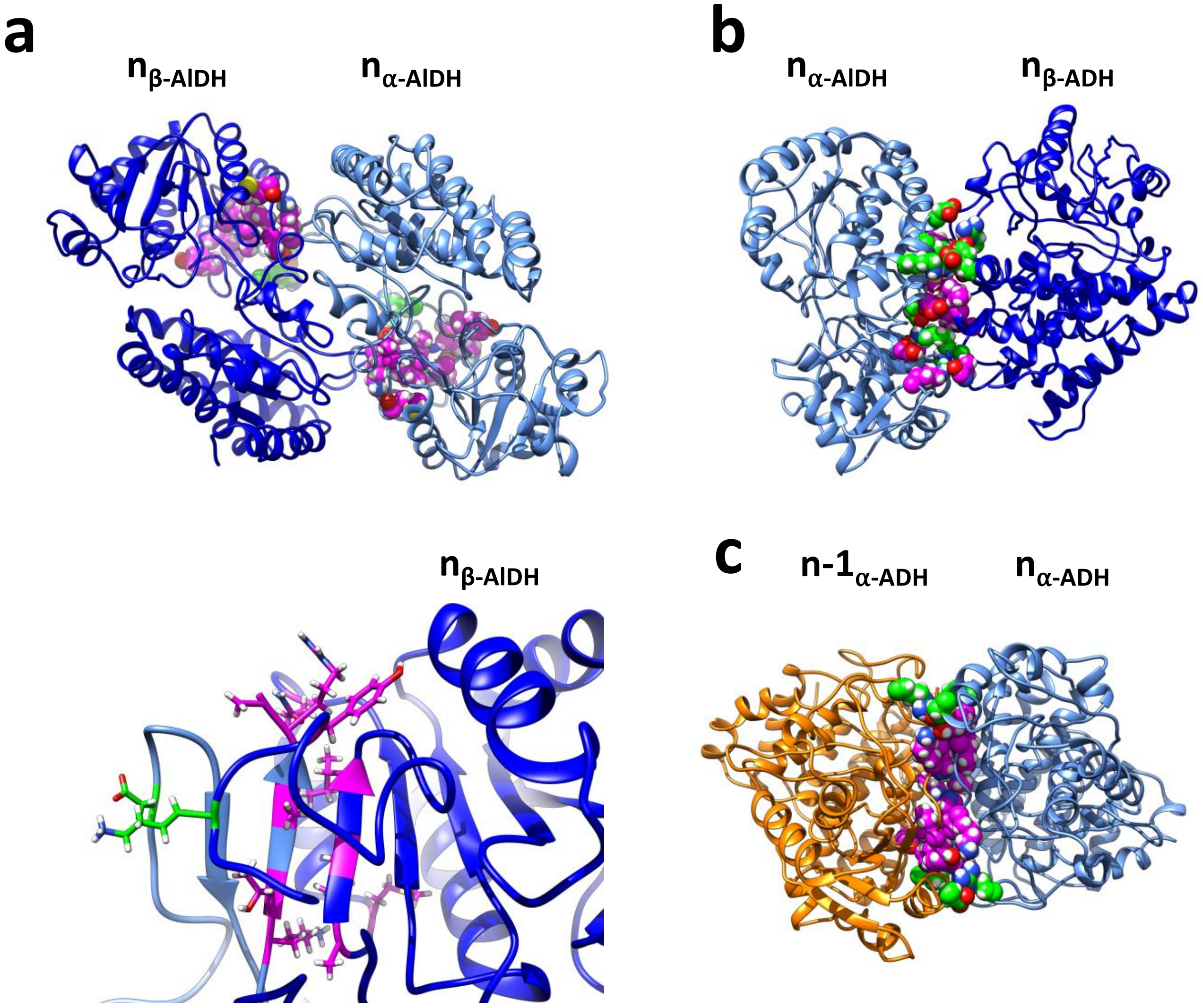

Supplementary Fig. 5. Representation of the residues involved in inter-domain interfaces within the extended spirosome. **a**. Ribbon representation of the acetaldehyde n_β (dark blue) – acetaldehyde n_α (light blue) dehydrogenase. Lower panel, zoomed representation of the main interface (beta-complementation) between the acetaldehyde n_β (dark blue) – acetaldehyde n_α (light blue) dehydrogenase **b.** Ribbon representation of the acetaldehyde n_α (light blue) – alcohol n_β (dark blue) dehydrogenase **c.** Ribbon representation of the alcohol $n\text{-}1_\alpha$ (orange) – alcohol n_α (light blue) dehydrogenase interfaces.

Residues involved in hydrogen bonds are displayed as spheres in pink and residues involved in salt bridges are displayed as spheres in green.

a

	ALDH	ADH	BOND
AlDH – ADH interface	GLU 63	TYR 848	H
		ILE 851	H
	HIS 70	ARG 572	H
	GLU 74	MET 574	H
	ARG 229	SER 764	H
	THR 239	LYS 759	H
	LYS 317	PRO 757	H
	ASP 44	ARG 572	SB
	ARG 46	ASP 838	SB
	LYS 51	GLU 834	SB
	ASP 64	LYS 759	SB
	ARG 229	ASP 767	SB

b

	AlDH	AlDH	BOND
AlDH – AlDH interface	GLN 375	ARG 447	H
	GLY 386	LYS 442	H
	MET 389		H
	ALA 392	THR 443	H
	ILE 394	ALA 445	H
	ILE 396	ARG 447	H
	TYR 409	ALA 448	H
	SER 430	LYS 441	H
	ASP 92	LYS 412	SB

c

	ADH	ADH	BOND
ADH – ADH interface	LEU 452	PHE 462	H
	HIS 454	ILE 460	H
	LYS 455	TYR 461	H
	GLU 669	SER 705	H
	PHE 670	GLN 674	H
	LYS 455	GLU 473	SB
	ARG 463	GLU 669	SB
	GLU 473	LYS 455	SB
		ARG 577	SB

Supplementary Fig. 6. PISA analysis of the 3 interfaces. Residues involved in hydrogen bonds and salt bridges are marked H and SB respectively. **a.** acetaldehyde (AlDH) – alcohol (ADH) dehydrogenase interface, **b.** acetaldehyde (AlDH) – acetaldehyde (AlDH) dehydrogenase interface **c.** alcohol (ADH) – alcohol (ADH) dehydrogenase interface.

a

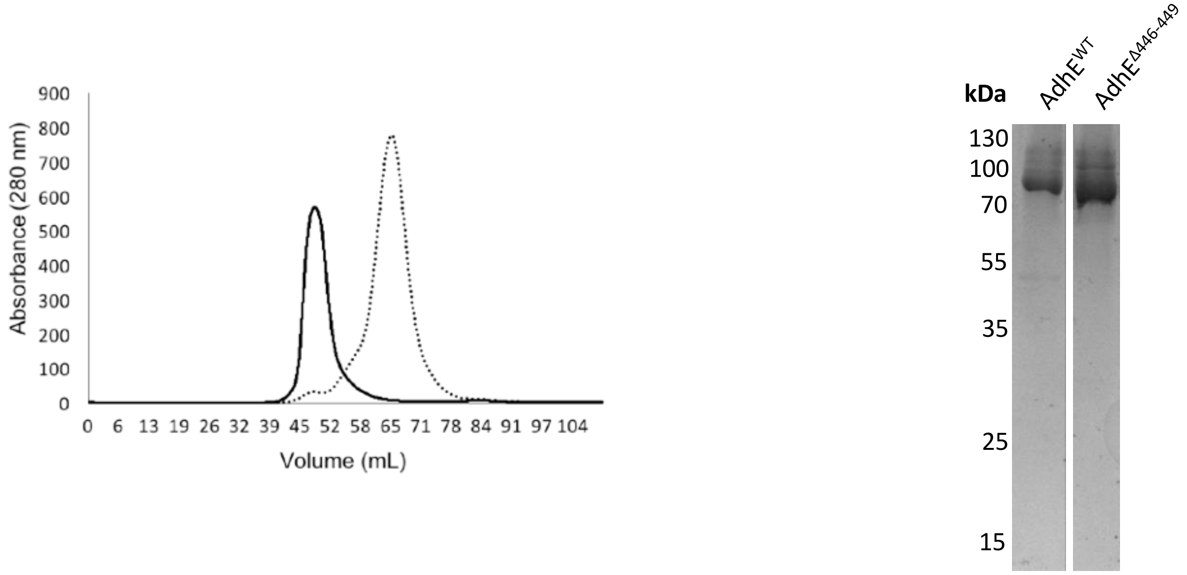

b

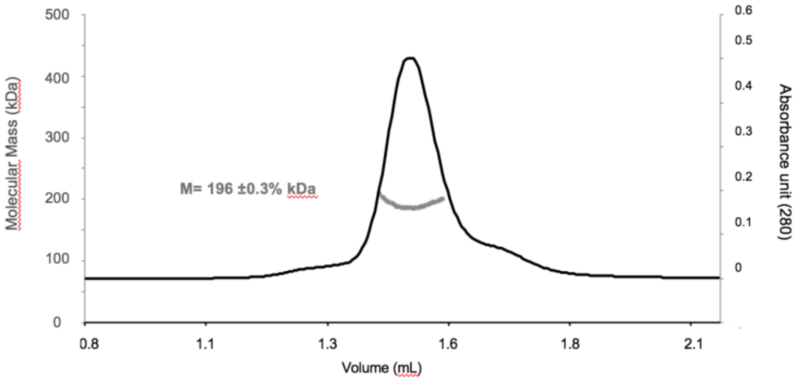

Supplementary Fig. 7. Expression test and purification of AdhE^{WT} and AdhE$^{\Delta446\text{-}449}$. a. Size-exclusion chromatography profile of purified AdhE^{WT} (dark line) and AdhE$^{\Delta446\text{-}449}$ (dashed line) on a HiLoad 16/600 Superdex 200 column (left) and SDS-PAGE (12%) of AdhE^{WT} and AdhE$^{\Delta446\text{-}449}$ (right). Molecular weight markers are indicated on the left. **b.** Size-exclusion profile of purified AdhE$^{\Delta446\text{-}449}$ on a Superdex S200 5/150 column coupled with MALS and RI.

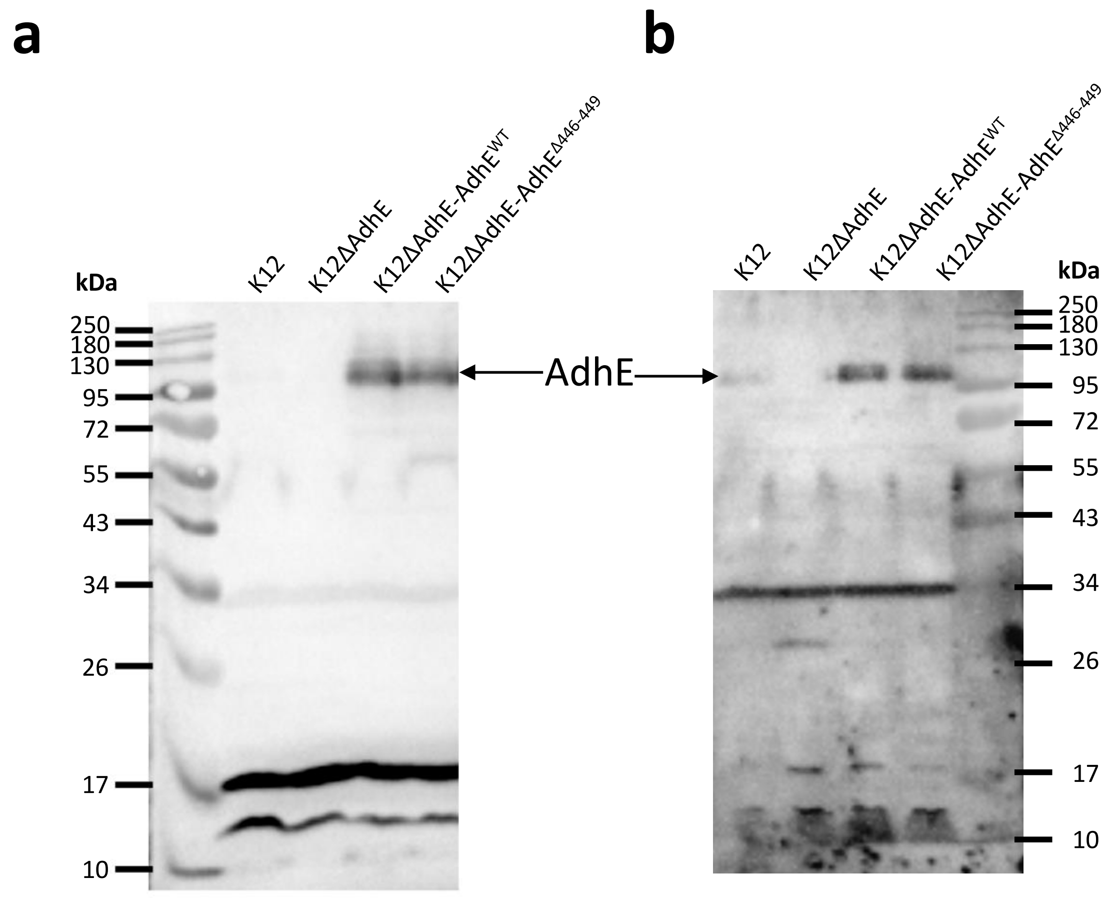

Supplementary Fig. 8. Western-blot profile of *E. coli* lysate expressing Wild-Type AdhE and Δ446-449 AdhE

a. Western-blot showing Wild-Type AdhE and Δ446-449 AdhE expression after induction in aerobic condition
b. Western-blot showing native AdhE, Wild-Type AdhE and Δ446-449 AdhE after anaerobic growth in minimal media

Primer	Sequence
AdhE_reverse	AAGGGGTTATGCTAGTTAGCGGATTTTTTCGCTTTTTTCTCAG
AdhE_foward	CCGCGCGGCAGCCATATGGCTGTTACTAATGTCGCTGA
AdhE446-449del_reverse	AGCAACGGTTTTCTTGTTG
AdhE446-449del_foward	AACATGTTGTGGCACAAAC
PKG116_reverse	CATATGGTACTCCTTATGGCATTATTGAT
PKG116_foward	GGATCCGCGGATAAATAAGTAACG
AdhE_reverse_PKG116	TTACTTATTTATCCGCGGATCCGGCCGTTTATGTTGCCAG
AdhE_foward_PKG116	CAATAATGCCATAAGGAGTACCATATGCATCATCACCATCACCACAGC GCGGGCGCAAGTGCAATGGCTGTTACTAATGTCGC

Strains	Source
K12 Escherichia coli	Our lab
BL21 (DE3) E. coli	Our lab
K12ΔAdhE	Keio collection[22]

Supplementary Fig. 9. Primer sequences and bacteria strains used
