# Supplementary material for: Filamentation of the bacterial bi-functional alcohol/aldehyde dehydrogenase AdhE is essential for substrate channeling and enzymatic regulation
Source: Nat Commun. 2020 Mar 18;11:1426. doi: 10.1038/s41467-020-15214-y (PMC7080775; doi:10.1038/s41467-020-15214-y)
Supplement: Supplementary file 3 — Description of Additional Supplementary Information [file 41467_2020_15214_MOESM3_ESM.pdf]

Description of Additional Supplementary Files

File Name: Supplementary Data 1
Description: Multiple sequence alignment of AlDH domain of *E. coli* AdhE and monofunctional AlDH enzymes.

File Name: Supplementary Data 2
Description: Multiple sequence alignment of ADH domain of *E. coli* AdhE and monofunctional ADH enzymes**.**

File Name: Supplementary Data 3
Description: Multiple sequence alignment of E. coli AdhE and oher AdhE homologs.
