# Supplementary material for: Filamentation of the bacterial bi-functional alcohol/aldehyde dehydrogenase AdhE is essential for substrate channeling and enzymatic regulation
Source: Nat Commun. 2020 Mar 18;11:1426. doi: 10.1038/s41467-020-15214-y (PMC7080775; doi:10.1038/s41467-020-15214-y)
Supplement: Supplementary file 5 — Supplementary Data 2 [file 41467_2020_15214_MOESM5_ESM.pptx]

## Slide 1
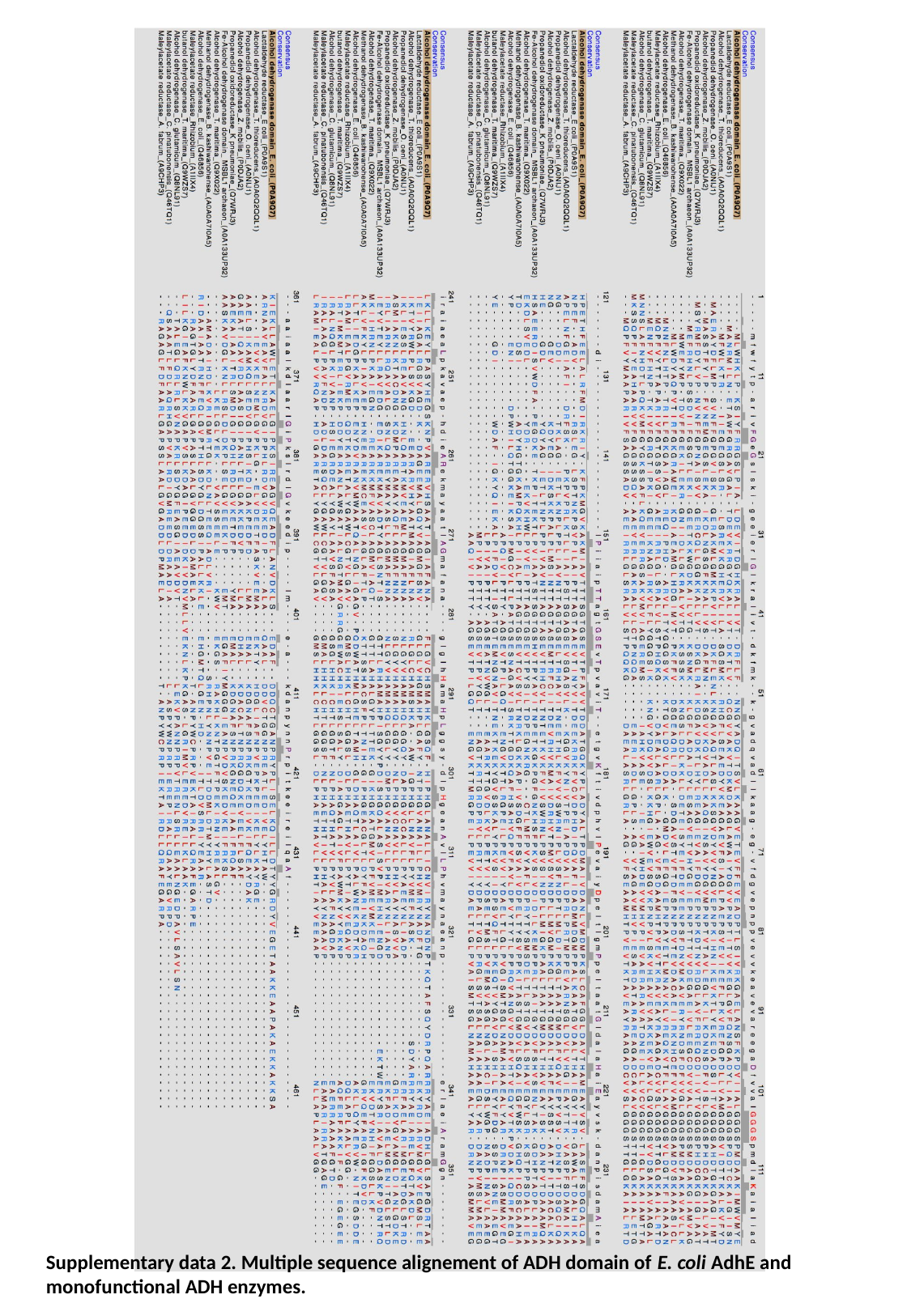

Supplementary data 2. Multiple sequence alignement of ADH domain of E. coli AdhE and monofunctional ADH enzymes.
