# Supplementary material for: Filamentation of the bacterial bi-functional alcohol/aldehyde dehydrogenase AdhE is essential for substrate channeling and enzymatic regulation
Source: Nat Commun. 2020 Mar 18;11:1426. doi: 10.1038/s41467-020-15214-y (PMC7080775; doi:10.1038/s41467-020-15214-y)
Supplement: Supplementary file 7 — Reporting Summary [file 41467_2020_15214_MOESM7_ESM.pdf]

nature research

Corresponding author(s): Rémi Fronzes

Last updated by author(s): Dec 16, 2019

Reporting Summary

Nature Research wishes to improve the reproducibility of the work that we publish. This form provides structure for consistency and transparency in reporting. For further information on Nature Research policies, see Authors & Referees and the Editorial Policy Checklist .

Statistics

For all statistical analyses, confirm that the following items are present in the figure legend, table legend, main text, or Methods section.

n/a	Confirmed	
☐	☒	The exact sample size (*n*) for each experimental group/condition, given as a discrete number and unit of measurement
☐	☒	A statement on whether measurements were taken from distinct samples or whether the same sample was measured repeatedly
☒	☐	The statistical test(s) used AND whether they are one- or two-sided<br>*Only common tests should be described solely by name; describe more complex techniques in the Methods section.*
☒	☐	A description of all covariates tested
☒	☐	A description of any assumptions or corrections, such as tests of normality and adjustment for multiple comparisons
☒	☐	A full description of the statistical parameters including central tendency (e.g. means) or other basic estimates (e.g. regression coefficient) AND variation (e.g. standard deviation) or associated estimates of uncertainty (e.g. confidence intervals)
☒	☐	For null hypothesis testing, the test statistic (e.g. *F*, *t*, *r*) with confidence intervals, effect sizes, degrees of freedom and *P* value noted<br>*Give P values as exact values whenever suitable.*
☒	☐	For Bayesian analysis, information on the choice of priors and Markov chain Monte Carlo settings
☒	☐	For hierarchical and complex designs, identification of the appropriate level for tests and full reporting of outcomes
☒	☐	Estimates of effect sizes (e.g. Cohen's *d*, Pearson's *r*), indicating how they were calculated

Our web collection on statistics for biologists contains articles on many of the points above.

Software and code

Policy information about availability of computer code

Data collection	Serial EM 3.7.10 : cryo-EM data collection<br>Tecan i-control (1.10.4.0)
Data analysis	Image treatment:<br>ImageJ (1.51s) (https://imagej.nih.gov/ij/index.html)<br><br>Cryo-EM data analysis:<br>gCTF (1.18)<br>MotionCor2 (1.1.0)<br>RELION (3.0.7)<br><br>Structure building:<br>Swiss-model (https://swissmodel.expasy.org)<br>Coot (0.8.9.1)<br>Phenix (1.14-3260)<br>Phyre (http://www.sbg.bio.ic.ac.uk/phyre2/)<br>PISA (http://www.ebi.ac.uk/pdbe/pisa/)<br>UCSF Chimera (1.14.0)<br><br>Structure validation:<br>MolProbity (http://molprobity.biochem.duke.edu/)

For manuscripts utilizing custom algorithms or software that are central to the research but not yet described in published literature, software must be made available to editors/reviewers. We strongly encourage code deposition in a community repository (e.g. GitHub). See the Nature Research guidelines for submitting code & software for further information.

Data

Policy information about availability of data

All manuscripts must include a data availability statement. This statement should provide the following information, where applicable:

- Accession codes, unique identifiers, or web links for publicly available datasets
- A list of figures that have associated raw data
- A description of any restrictions on data availability

Provide your data availability statement here.

Field-specific reporting

Please select the one below that is the best fit for your research. If you are not sure, read the appropriate sections before making your selection.

☒ Life sciences ☐ Behavioural & social sciences ☐ Ecological, evolutionary & environmental sciences

For a reference copy of the document with all sections, see nature.com/documents/nr-reporting-summary-flat.pdf

Life sciences study design

All studies must disclose on these points even when the disclosure is negative.

Sample size	No statistical methods were used to determine sample sizes.
Data exclusions	No data were excluded from the analysis
Replication	All attempts at replication were successful. Experiments were done in triplicate, each with three independent biological samples. Errors bar indicated in figure represent standard error of the mean.<br>All information are indicated in the Methods section, legend and corresponding figures.
Randomization	All experiments were performed with clonal populations.
Blinding	No blinding was performed as the acquisition and analysis methods require human intervention.

Reporting for specific materials, systems and methods

We require information from authors about some types of materials, experimental systems and methods used in many studies. Here, indicate whether each material, system or method listed is relevant to your study. If you are not sure if a list item applies to your research, read the appropriate section before selecting a response.

Materials & experimental systems

n/a	Involved in the study	
☐	☒	Antibodies
☒	☐	Eukaryotic cell lines
☒	☐	Palaeontology
☒	☐	Animals and other organisms
☒	☐	Human research participants
☒	☐	Clinical data

Methods

n/a	Involved in the study	
☒	☐	ChIP-seq
☒	☐	Flow cytometry
☒	☐	MRI-based neuroimaging

Antibodies

Antibodies used	Commercial antibodies:<br>-Rabbit polyclonal anti-alcohol/acetaldehyde dehydrogenase (Agrisera, Cat# AS10 748, lot# 451, dilution 1/1000)<br>-Peroxidase AffiniPure Donkey Anti-Rabbit secondary antibody (Jackson ImmunoResearch, Cat# 711-035-152, Lot# 129517, dilution 1/5000)
Validation	Antibody was validated in western-blot with samples expressing or non-expressing protein.<br>Laurenceau et al. (2015). Conserved Streptococcus pneumoniae Spirosomes Suggest a Single Type of Transformation Pilus in Competence. PLoS Pathog. 2015 Apr 15;11(4):e1004835. doi: 10.1371/journal.ppat.1004835.
