# Supplementary material for: Filamentation of the bacterial bi-functional alcohol/aldehyde dehydrogenase AdhE is essential for substrate channeling and enzymatic regulation
Source: Nat Commun. 2020 Mar 18;11:1426. doi: 10.1038/s41467-020-15214-y (PMC7080775; doi:10.1038/s41467-020-15214-y)
Supplement: Supplementary file 8 — Source Data [file 41467_2020_15214_MOESM8_ESM.pdf]

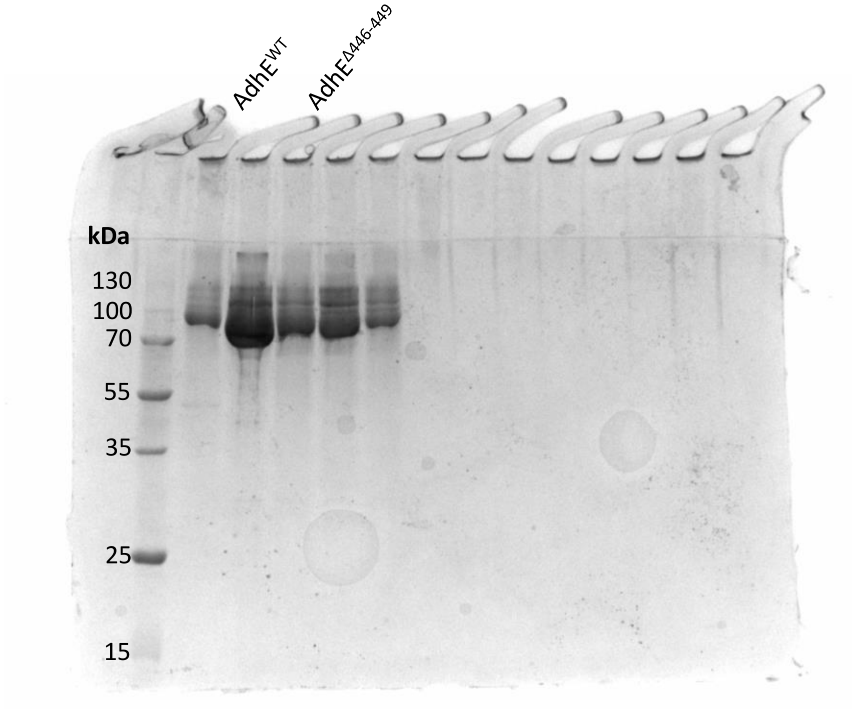

SDS-PAGE (12%) of purified AdhE^{WT} and AdhE$^{\Delta 446-449}$. Molecular weight (PageRuler Plus Prestained Protein Ladder (10-250kDa)) are indicated on the left. **b.** Size-exclusion chromatography profile of purified AdhE^{WT} on a HiLoad 16/600 Superdex 200 pg column.

a

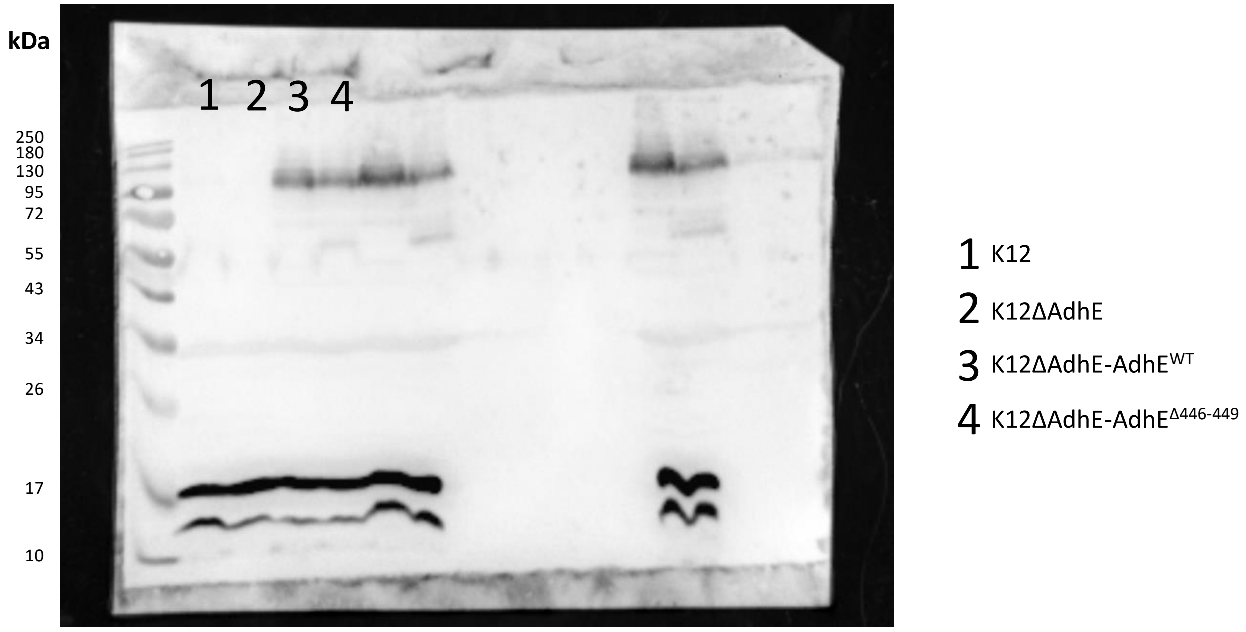

b

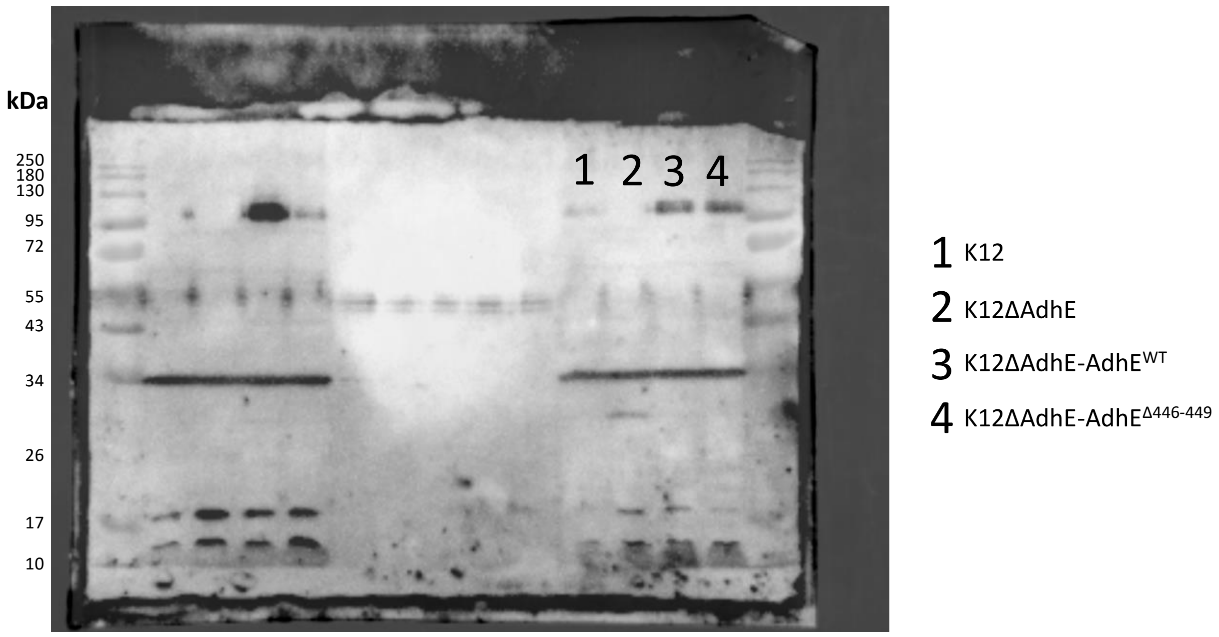

Western-blot profile of *E. coli* lysate expressing AdhE^{WT} and AdhE$^{Δ446-449}$. Molecular weight (Color Prestained Protein Standard, Broad Range (10-250kDa)) are indicated on the left.

a. Western-blot showing Wild-Type AdhE and Δ446-449 AdhE expression after induction in aerobic condition
b. Western-blot showing native AdhE, Wild-Type AdhE and Δ446-449 AdhE after anaerobic growth in minimal media

				WT AcetylCoA - Ethanol pH7		
Time (ms)		1			2	
0	0.7288	0.72170001	0.71969998	0.68339998	0.65869999	0.65170002
1.46881667	0.73150003	0.72030002	0.71679997	0.71960002	0.64889997	0.6433
2.93746667	0.73250002	0.72280002	0.7191	0.72549999	0.67189997	0.63069999
4.40608333	0.73509997	0.7227	0.71899998	0.7209	0.66119999	0.62639999
6.53145	0.7306	0.71939999	0.71850002	0.72030002	0.68080002	0.61879998
8.0001	0.7288	0.71569997	0.71439999	0.71670002	0.68959999	0.6257
9.46875	0.72860003	0.72100002	0.71749997	0.72180003	0.70090002	0.62940001
10.9374	0.73229998	0.71859998	0.71539998	0.71619999	0.70230001	0.63239998
12.4065667	0.7295	0.71359998	0.71100003	0.7159	0.71069998	0.63679999
13.8752167	0.7263	0.7173	0.71460003	0.71490002	0.713	0.63590002
15.34435	0.7299	0.7177	0.71210003	0.71079999	0.71460003	0.63319999
16.8135167	0.7299	0.71319997	0.71060002	0.70969999	0.71249998	0.63709998
18.2825	0.72530001	0.71420002	0.71060002	0.71530002	0.71649998	0.63870001
19.7512833	0.7245	0.71460003	0.70920002	0.70700002	0.71380001	0.63889998
21.21995	0.72469997	0.71609998	0.71149999	0.70060003	0.71450001	0.63779998
22.6887833	0.72430003	0.71520001	0.71130002	0.70209998	0.71380001	0.64069998
24.1576	0.72289997	0.7141	0.70770001	0.70569998	0.7141	0.63730001
25.6262333	0.72530001	0.71439999	0.71219999	0.7069	0.71340001	0.63859999
27.0949167	0.72299999	0.71170002	0.70929998	0.70099998	0.71109998	0.63880002
28.5637	0.72219998	0.71439999	0.70859998	0.69910002	0.71460003	0.64139998
30.0326833	0.72100002	0.7119	0.70719999	0.7001	0.71139997	0.64130002
31.5017	0.72430003	0.71520001	0.71069998	0.6997	0.71259999	0.64230001
32.9703333	0.72420001	0.71109998	0.70459998	0.69980001	0.71030003	0.63679999
34.4389667	0.71829998	0.71289998	0.70649999	0.70179999	0.71109998	0.63990003
35.9076333	0.72100002	0.71240002	0.70609999	0.70099998	0.70950001	0.63859999
37.3762667	0.72039998	0.71109998	0.70999998	0.70370001	0.71179998	0.63880002
38.84525	0.72009999	0.70999998	0.70490003	0.70139998	0.71280003	0.63800001
40.3140833	0.71990001	0.71039999	0.70569998	0.69859999	0.70920002	0.63660002
41.7827333	0.71859998	0.71039999	0.70880002	0.6997	0.70889997	0.63779998
43.2513833	0.71920002	0.70730001	0.70370001	0.70230001	0.71280003	0.64130002
44.7200667	0.71820003	0.71139997	0.704	0.7015	0.71069998	0.63880002
46.1886833	0.71689999	0.70850003	0.70469999	0.70050001	0.71030003	0.63630003
47.6573	0.71920002	0.70929998	0.7033	0.70130002	0.71149999	0.63849998
49.1263	0.71520001	0.70920002	0.70480001	0.69809997	0.71030003	0.63559997
50.59495	0.71789998	0.7098	0.70240003	0.69919997	0.70950001	0.63800001
52.0637667	0.69630003	0.66180003	0.69760001	0.50919998	0.65380001	0.66399997
53.5325833	0.61180001	0.60540003	0.65280002	0.52789998	0.62940001	0.6304
55.0012333	0.5851	0.58499998	0.62040001	0.56300002	0.61150002	0.6045
56.47005	0.56760001	0.57209998	0.59859997	0.55519998	0.59930003	0.59859997
57.93885	0.55650002	0.55720001	0.5808	0.54519999	0.58170003	0.59200001
59.4075	0.542	0.54689997	0.56519997	0.53070003	0.57099998	0.58859998
60.87635	0.53049999	0.53320003	0.54720002	0.52039999	0.5575	0.5826
62.34515	0.51849997	0.52060002	0.53289998	0.5018	0.54710001	0.58329999
63.8138	0.50770003	0.50830001	0.51840001	0.48859999	0.53539997	0.58029997

0.14686667	0.49509999	0.4941	0.50520003	0.48019999	0.52700001	0.57859999
0.29373333	0.48390001	0.48640001	0.4932	0.47229999	0.51370001	0.579
0.44056667	0.4781	0.4763	0.4833	0.46020001	0.50849998	0.57609999
0.58761667	0.4659	0.46630001	0.4711	0.4479	0.49649999	0.57279998
0.7345	0.4598	0.46039999	0.4632	0.43970001	0.49340001	0.57440001
0.88135	0.45140001	0.45190001	0.45559999	0.43279999	0.4849	0.5758
1.02821667	0.442	0.44240001	0.44490001	0.42460001	0.4781	0.57489997
1.17506667	0.43790001	0.43849999	0.44150001	0.4102	0.46919999	0.57209998
1.32193333	0.43290001	0.43110001	0.4325	0.40560001	0.46830001	0.57389998
1.61565	0.42629999	0.426	0.42469999	0.40169999	0.46450001	0.57440001
1.76255	0.42359999	0.42179999	0.42129999	0.39399999	0.45359999	0.57349998
1.9094	0.41859999	0.41670001	0.41679999	0.38960001	0.44940001	0.57099998
2.05625	0.41299999	0.41080001	0.40979999	0.38209999	0.4452	0.57419997
2.20313333	0.4093	0.40920001	0.40830001	0.37180001	0.43900001	0.5711
2.35	0.4039	0.4039	0.39829999	0.36930001	0.43560001	0.57419997
2.49685	0.40040001	0.39860001	0.39809999	0.3651	0.43220001	0.57480001
2.64371667	0.39750001	0.39629999	0.3926	0.35780001	0.4314	0.5733
2.79058333	0.39579999	0.3953	0.39250001	0.3567	0.42660001	0.57309997
3.0843	0.3899	0.3883	0.3863	0.35440001	0.42140001	0.57200003
3.23118333	0.38600001	0.38550001	0.38420001	0.3522	0.41909999	0.57139999
3.37805	0.38370001	0.384	0.37959999	0.34670001	0.41420001	0.5704
3.52491667	0.37920001	0.38139999	0.37560001	0.34029999	0.41280001	0.57300001
3.67176667	0.37630001	0.37720001	0.37419999	0.3389	0.41139999	0.5733
3.81866667	0.37529999	0.3761	0.3689	0.33469999	0.40970001	0.57050002
3.96551667	0.37200001	0.37380001	0.36930001	0.33219999	0.4041	0.5704
4.11236667	0.36759999	0.36930001	0.3644	0.3292	0.4023	0.56849998
4.25923333	0.36739999	0.36939999	0.3644	0.32780001	0.40130001	0.57169998
4.55298333	0.36390001	0.36570001	0.3608	0.32370001	0.3996	0.56980002
4.69983333	0.36289999	0.36320001	0.35679999	0.32539999	0.3976	0.5704
4.84668333	0.36090001	0.3619	0.35730001	0.31670001	0.39210001	0.57029998
4.99355	0.3603	0.35859999	0.35319999	0.31909999	0.3933	0.56980002
5.79713333	0.35690001	0.35859999	0.35280001	0.31529999	0.39179999	0.56889999
5.944	0.3538	0.35499999	0.35069999	0.31349999	0.3892	0.57029998
6.09086667	0.35120001	0.35229999	0.34900001	0.31130001	0.3888	0.56950003
6.23773333	0.34740001	0.3515	0.34720001	0.30989999	0.38839999	0.57120001
6.3846	0.3511	0.35139999	0.34349999	0.30700001	0.38330001	0.56840003
6.67831667	0.34599999	0.34619999	0.34079999	0.30340001	0.38170001	0.56760001
6.82518333	0.345	0.34799999	0.34150001	0.3028	0.3829	0.56999999
6.97206667	0.34259999	0.34419999	0.33770001	0.30219999	0.37740001	0.56720001
7.11893333	0.34099999	0.34009999	0.33649999	0.30230001	0.37819999	0.5693
7.26578333	0.34060001	0.3418	0.33570001	0.2965	0.3752	0.56830001
7.41265	0.33750001	0.34200001	0.33419999	0.294	0.3707	0.5693
7.55951667	0.33919999	0.3414	0.3348	0.29519999	0.37180001	0.56889999
7.70638333	0.3362	0.33590001	0.32969999	0.29179999	0.36849999	0.5661
7.85326667	0.3337	0.3326	0.3263	0.2911	0.36899999	0.56690001
8.14698333	0.33160001	0.3348	0.3292	0.29280001	0.36770001	0.56770003

8.29385	0.3303	0.33239999	0.32730001	0.29350001	0.3687	0.56620002
8.4407	0.33090001	0.33199999	0.32600001	0.2902	0.36399999	0.5693
8.58758333	0.32749999	0.33039999	0.32480001	0.28569999	0.36199999	0.56599998
8.73443333	0.32600001	0.32859999	0.32339999	0.2841	0.36140001	0.565
8.8813	0.32589999	0.32730001	0.32100001	0.2843	0.36250001	0.56580001
9.02816667	0.32460001	0.32359999	0.3197	0.28259999	0.3612	0.56699997
9.17501667	0.31979999	0.32499999	0.3168	0.2834	0.3583	0.56669998
9.3219	0.32190001	0.32429999	0.31810001	0.2823	0.35890001	0.56660002
9.61561667	0.32120001	0.32499999	0.31850001	0.27829999	0.354	0.5668
9.7625	0.32010001	0.32139999	0.31470001	0.2807	0.35749999	0.56650001
9.90936667	0.31999999	0.32210001	0.31580001	0.2782	0.35609999	0.56660002
10.0562333	0.3177	0.3202	0.31380001	0.278	0.35460001	0.56739998
10.2030833	0.3159	0.32069999	0.31200001	0.27500001	0.3511	0.56449997
10.34995	0.3168	0.31999999	0.3127	0.27399999	0.3504	0.56339997
10.4968	0.31720001	0.3195	0.3136	0.27309999	0.34990001	0.56569999
10.6436833	0.31349999	0.3177	0.30989999	0.27410001	0.35100001	0.56550002
10.79055	0.31200001	0.3184	0.30860001	0.27410001	0.3495	0.5661
11.0842667	0.3134	0.3154	0.30829999	0.27239999	0.34619999	0.56379998
11.2311167	0.3105	0.31290001	0.3057	0.27149999	0.34599999	0.565
11.378	0.31	0.31330001	0.30680001	0.26989999	0.34709999	0.56550002
11.52505	0.30899999	0.31099999	0.30399999	0.27020001	0.34459999	0.56559998
11.6719333	0.308	0.31079999	0.3035	0.26840001	0.3461	0.56389999
11.81875	0.30790001	0.31009999	0.30410001	0.2676	0.3434	0.5643
11.9659667	0.30649999	0.3082	0.30149999	0.2658	0.34330001	0.56440002
12.1128333	0.3066	0.31	0.3019	0.2692	0.34009999	0.56440002
12.2597167	0.30250001	0.3055	0.3003	0.2649	0.33860001	0.56349999
12.5534333	0.3037	0.3055	0.301	0.26460001	0.3387	0.56239998
12.7002833	0.30469999	0.31110001	0.30180001	0.26109999	0.3355	0.56199998
12.84715	0.3019	0.3044	0.30000001	0.26460001	0.33970001	0.56410003
12.9940167	0.30360001	0.3055	0.30019999	0.25960001	0.33680001	0.56190002
13.1408667	0.30090001	0.30509999	0.29879999	0.26269999	0.3348	0.5589
13.28775	0.3012	0.30199999	0.2974	0.26089999	0.33590001	0.56279999
13.4346167	0.30039999	0.30160001	0.2942	0.26030001	0.33649999	0.56209999
13.5814667	0.30019999	0.30199999	0.29640001	0.25929999	0.3339	0.5636
13.7283667	0.29750001	0.3008	0.2951	0.259	0.3326	0.56
14.0224167	0.29789999	0.3008	0.29409999	0.25799999	0.333	0.56110001
14.1692667	0.2974	0.29800001	0.29409999	0.25729999	0.33250001	0.56279999
14.3161333	0.29800001	0.29949999	0.29429999	0.25639999	0.3312	0.55980003
14.463	0.2956	0.2994	0.29249999	0.25659999	0.33219999	0.56370002
14.6098667	0.2933	0.2949	0.28819999	0.25580001	0.32789999	0.55879998
14.7567333	0.29519999	0.2983	0.29080001	0.25619999	0.33070001	0.5607
14.9036	0.29350001	0.29609999	0.28749999	0.25639999	0.32789999	0.56169999
15.0504833	0.29100001	0.2949	0.2888	0.2529	0.32879999	0.55860001
15.1975	0.2931	0.29390001	0.28799999	0.25220001	0.32539999	0.56290001
15.4913833	0.29049999	0.29460001	0.28549999	0.25220001	0.3256	0.56129998
15.6382667	0.29350001	0.2958	0.2899	0.25400001	0.3276	0.56160003

15.7851167	0.29100001	0.294	0.28659999	0.25099999	0.32730001	0.56010002
15.9323167	0.2881	0.29249999	0.28310001	0.25229999	0.32440001	0.56150001
16.0791833	0.2906	0.2904	0.2827	0.2529	0.3285	0.56279999
16.22605	0.28819999	0.29089999	0.2859	0.25009999	0.32359999	0.56059998
16.37295	0.28799999	0.29280001	0.28459999	0.24770001	0.32089999	0.56209999
16.5198	0.28960001	0.29089999	0.2827	0.24519999	0.3215	0.55900002
16.66665	0.28569999	0.29010001	0.28310001	0.2491	0.32339999	0.56089997
16.9603833	0.2863	0.28999999	0.28389999	0.2483	0.32370001	0.5618
17.10725	0.2859	0.2902	0.2845	0.2476	0.32210001	0.5607
17.2541167	0.28459999	0.28780001	0.2811	0.2449	0.31909999	0.55870003
17.4009667	0.2834	0.28549999	0.2818	0.24600001	0.31959999	0.56110001
17.5478333	0.28420001	0.28780001	0.28	0.2463	0.32030001	0.55989999
17.6947	0.28310001	0.28659999	0.27939999	0.2455	0.31830001	0.56
17.8415667	0.28310001	0.28569999	0.27700001	0.2429	0.31869999	0.55919999
17.9884333	0.28169999	0.28819999	0.28029999	0.2456	0.31850001	0.56019998
18.13565	0.2825	0.28549999	0.27959999	0.24349999	0.3159	0.55650002
18.4293667	0.28380001	0.2872	0.27900001	0.2441	0.31600001	0.56040001
18.5762333	0.28119999	0.28349999	0.27599999	0.24699999	0.3179	0.56199998
18.7231	0.28	0.2852	0.2762	0.2437	0.31810001	0.56199998
18.8699833	0.28279999	0.2836	0.2755	0.244	0.3136	0.55970001
19.0169833	0.28259999	0.2852	0.27590001	0.242	0.31600001	0.56080002
19.16385	0.27849999	0.28130001	0.27270001	0.24070001	0.3136	0.55940002
19.3107	0.28130001	0.2841	0.27869999	0.24150001	0.3132	0.55940002
19.4575833	0.27970001	0.28380001	0.27700001	0.243	0.31400001	0.56010002
19.6044333	0.27739999	0.28040001	0.27399999	0.2427	0.3125	0.55970001
19.8981833	0.27829999	0.27900001	0.27270001	0.2419	0.31549999	0.5614
20.0450667	0.2775	0.28150001	0.2721	0.2404	0.31400001	0.56169999
20.1919167	0.27669999	0.2791	0.2714	0.2388	0.31029999	0.55830002
20.3387833	0.27540001	0.27770001	0.27219999	0.2393	0.31200001	0.55860001
20.4856333	0.2748	0.2748	0.27079999	0.2392	0.31200001	0.55769998
20.6325	0.27739999	0.27880001	0.27289999	0.23999999	0.31040001	0.55769998
20.7793667	0.27469999	0.2789	0.271	0.2377	0.3116	0.55839998
20.9262333	0.27579999	0.2789	0.2719	0.2369	0.31	0.5618
21.0730833	0.27360001	0.27810001	0.271	0.23800001	0.3114	0.55909997
21.3668333	0.2739	0.27739999	0.26879999	0.2332	0.30720001	0.55699998
21.5137	0.2735	0.2748	0.26820001	0.23559999	0.30770001	0.55839998
21.6605667	0.2744	0.27689999	0.26809999	0.2374	0.30790001	0.55800003
21.8074333	0.27219999	0.27579999	0.26710001	0.23630001	0.308	0.5582
21.9542833	0.27270001	0.27669999	0.26800001	0.2357	0.3071	0.55599999
22.1013	0.27160001	0.27469999	0.2685	0.2359	0.3053	0.5564
22.2481833	0.27039999	0.2735	0.2658	0.235	0.30520001	0.55739999
22.3950667	0.27059999	0.2753	0.26879999	0.23540001	0.3037	0.55589998
22.5419	0.2696	0.2744	0.26789999	0.235	0.3055	0.55680001
22.8356333	0.27149999	0.27579999	0.2674	0.23549999	0.30680001	0.55860001
22.9825167	0.27020001	0.27500001	0.26719999	0.2331	0.30579999	0.55930001
23.1293667	0.27039999	0.27289999	0.2658	0.2341	0.3055	0.55860001

23.27625	0.26840001	0.2728	0.26710001	0.2331	0.3037	0.55739999
23.4231167	0.26890001	0.27180001	0.26539999	0.2334	0.3055	0.55989999
23.5699667	0.26730001	0.27160001	0.26499999	0.2333	0.30219999	0.55879998
23.717	0.26949999	0.27309999	0.26660001	0.2316	0.3028	0.55470002
23.8638667	0.2696	0.2723	0.264	0.2324	0.30160001	0.55720001
24.0107333	0.2694	0.27079999	0.2638	0.2313	0.3012	0.55409998
24.30445	0.26859999	0.27329999	0.2642	0.23280001	0.30270001	0.55970001
24.4513333	0.26879999	0.27090001	0.26620001	0.23	0.30129999	0.55629998
24.5981833	0.26589999	0.2683	0.26210001	0.2308	0.30160001	0.55589998
24.7450667	0.2669	0.27070001	0.26300001	0.2291	0.29859999	0.55409998
24.89195	0.26570001	0.2685	0.26199999	0.2295	0.29980001	0.5582
25.0387833	0.26679999	0.271	0.2626	0.22920001	0.2985	0.55489999
25.18565	0.2651	0.26840001	0.26249999	0.2317	0.3012	0.55549997
25.3325333	0.26530001	0.26699999	0.26100001	0.227	0.29800001	0.55629998
25.4793833	0.26429999	0.26710001	0.2604	0.2295	0.2994	0.55559999
25.7731167	0.2647	0.26840001	0.2615	0.23	0.30000001	0.55809999
25.9199667	0.26449999	0.26820001	0.2622	0.22589999	0.29800001	0.55409998
26.0668167	0.26300001	0.26769999	0.25960001	0.2261	0.2958	0.5535
26.2137167	0.2617	0.2665	0.26050001	0.2271	0.2949	0.55290002
26.3605667	0.264	0.2694	0.2604	0.2274	0.29660001	0.55269998
26.5074333	0.26300001	0.26589999	0.25960001	0.2261	0.2951	0.5535
26.6543167	0.26210001	0.26440001	0.25889999	0.2277	0.2965	0.5557
26.8011667	0.2597	0.2656	0.25600001	0.22570001	0.2965	0.55470002
26.9480333	0.2622	0.2656	0.2588	0.2273	0.29609999	0.55610001
27.2417667	0.26199999	0.26609999	0.26010001	0.22849999	0.29750001	0.5571
27.3887	0.26280001	0.26550001	0.2606	0.227	0.29699999	0.55519998
27.5356667	0.26210001	0.26359999	0.2588	0.2261	0.2942	0.5535
27.6825333	0.26050001	0.264	0.255	0.2251	0.29589999	0.55610001
27.8293833	0.26109999	0.26660001	0.25709999	0.2263	0.29319999	0.55419999
27.9762667	0.2606	0.26289999	0.2561	0.2261	0.29519999	0.55559999
28.1231	0.26109999	0.26539999	0.259	0.2254	0.2931	0.55290002
28.2699833	0.25889999	0.2638	0.2543	0.22229999	0.29159999	0.55400002
28.41685	0.26050001	0.264	0.25729999	0.2234	0.29159999	0.5528
28.7105667	0.25729999	0.26300001	0.25420001	0.2212	0.29139999	0.5528
28.8574333	0.25889999	0.26249999	0.25420001	0.2235	0.29229999	0.5535
29.0043	0.2579	0.26179999	0.2545	0.2237	0.29190001	0.55269998
29.1511667	0.2595	0.2617	0.25470001	0.2253	0.29319999	0.5539
29.2980167	0.2545	0.2581	0.25229999	0.223	0.29139999	0.55150002
29.4449333	0.2577	0.2617	0.25150001	0.22319999	0.2924	0.55440003
29.59195	0.25819999	0.26230001	0.25220001	0.22229999	0.29280001	0.55309999
29.7389667	0.25639999	0.2599	0.25119999	0.2218	0.28830001	0.5539
29.8858167	0.2572	0.26140001	0.2552	0.22239999	0.2933	0.55229998
30.1795667	0.25670001	0.2615	0.25440001	0.2233	0.29170001	0.55400002
30.3264167	0.25619999	0.26019999	0.25119999	0.22050001	0.2897	0.55269998
30.4733	0.2552	0.25780001	0.25189999	0.2225	0.29049999	0.55299997
30.6202167	0.25709999	0.2588	0.25099999	0.2209	0.2886	0.5503

30.7670167	0.2568	0.26089999	0.25369999	0.2199	0.28929999	0.5528
30.9142167	0.25510001	0.25979999	0.25029999	0.2216	0.28889999	0.55199999
31.0610833	0.25529999	0.2586	0.25170001	0.21789999	0.28690001	0.55019999
31.20795	0.2529	0.2572	0.24950001	0.2198	0.287	0.55290002
31.3548167	0.25409999	0.25839999	0.25119999	0.2168	0.28670001	0.54979998
31.6485333	0.255	0.25819999	0.25139999	0.2211	0.2886	0.55320001
31.7954167	0.2518	0.257	0.2475	0.2168	0.28780001	0.55299997
31.9423167	0.25560001	0.25819999	0.25	0.21969999	0.28709999	0.55190003
32.0891333	0.25279999	0.25740001	0.2489	0.219	0.29030001	0.55199999
32.236	0.25080001	0.25639999	0.24699999	0.2199	0.28799999	0.551
32.3828667	0.2538	0.25749999	0.2491	0.2182	0.28420001	0.5528
32.5297333	0.25330001	0.25569999	0.24860001	0.21950001	0.2854	0.55000001
32.6766	0.2516	0.25639999	0.2474	0.2189	0.2868	0.55360001
32.82345	0.2529	0.25580001	0.2481	0.21619999	0.2843	0.54949999
33.1172	0.25260001	0.25709999	0.2492	0.2186	0.28580001	0.55119997
33.2640667	0.2511	0.25510001	0.24600001	0.22	0.28569999	0.54960001
33.4109333	0.25279999	0.25650001	0.25009999	0.2177	0.28510001	0.5492
33.5578167	0.2511	0.25470001	0.2484	0.2182	0.28889999	0.55239999
33.70465	0.2507	0.25479999	0.24680001	0.221	0.28560001	0.55180001
33.8515167	0.2511	0.2552	0.24699999	0.2181	0.28510001	0.5499
33.9983833	0.2493	0.25279999	0.2449	0.2172	0.2854	0.55070001
34.1452833	0.2511	0.2543	0.24699999	0.21799999	0.28529999	0.55159998
34.2920833	0.24959999	0.25569999	0.2471	0.2184	0.28650001	0.55220002
34.58585	0.24969999	0.2536	0.24439999	0.2168	0.2814	0.54930001
34.7327333	0.25040001	0.25319999	0.24510001	0.2172	0.2845	0.55110002
34.8795833	0.2494	0.25139999	0.2449	0.21529999	0.28130001	0.54970002
35.0264333	0.2507	0.25670001	0.2473	0.2176	0.285	0.55140001
35.1733	0.249	0.25260001	0.24519999	0.2146	0.28099999	0.54970002
35.3202	0.2476	0.25229999	0.2454	0.2146	0.28259999	0.54879999
35.4670333	0.2493	0.25299999	0.24680001	0.215	0.2841	0.54759997
35.6139	0.24600001	0.249	0.24519999	0.2172	0.2818	0.55059999
35.76075	0.25040001	0.25580001	0.2471	0.2149	0.2814	0.5521
36.0544833	0.25	0.25420001	0.2465	0.2131	0.28060001	0.54710001
36.2013667	0.2472	0.25170001	0.2448	0.21529999	0.2836	0.54790002
36.3482333	0.2483	0.2516	0.2457	0.21430001	0.28150001	0.55000001
36.4950833	0.24699999	0.252	0.2445	0.21430001	0.28119999	0.55049998
36.64195	0.2485	0.25319999	0.24609999	0.2156	0.2827	0.54890001
36.7888	0.249	0.25240001	0.24439999	0.2174	0.2823	0.55019999
36.9356833	0.2466	0.2498	0.2432	0.21250001	0.278	0.54619998
37.08255	0.2498	0.2511	0.24259999	0.21349999	0.27779999	0.54759997
37.2294333	0.2482	0.25279999	0.2448	0.2139	0.28259999	0.54869998
37.5231333	0.2448	0.2507	0.2419	0.21259999	0.27939999	0.5499
37.6700167	0.2471	0.2509	0.2432	0.2112	0.27950001	0.5456
37.8169	0.2481	0.2511	0.24529999	0.2177	0.2827	0.55110002
37.96375	0.24600001	0.25029999	0.24339999	0.2139	0.28099999	0.54900002
38.1106	0.2455	0.24770001	0.24169999	0.2155	0.2807	0.55080003

38.2574667	0.2454	0.249	0.24250001	0.21359999	0.28	0.55019999
38.4045	0.2422	0.2484	0.23999999	0.2142	0.27860001	0.5478
38.5513667	0.24250001	0.2476	0.2393	0.2133	0.28009999	0.54799998
38.6982667	0.24519999	0.24950001	0.2403	0.2131	0.27779999	0.54820001
38.9921167	0.24600001	0.2481	0.2403	0.21259999	0.27900001	0.54939997
39.1389667	0.24600001	0.2498	0.24089999	0.2103	0.2789	0.54540002
39.2858333	0.2438	0.24779999	0.2397	0.21179999	0.27720001	0.54710001
39.43275	0.2463	0.25150001	0.24169999	0.2139	0.2764	0.54930001
39.5797667	0.244	0.24789999	0.23999999	0.21170001	0.27869999	0.54720002
39.7266167	0.2449	0.2489	0.2428	0.2133	0.2771	0.54720002
39.8734667	0.2414	0.2455	0.2388	0.21340001	0.2793	0.5503
40.0203333	0.2439	0.2493	0.24089999	0.2148	0.27759999	0.54750001
40.1672333	0.24240001	0.2473	0.2411	0.2113	0.2782	0.5492
40.4609333	0.2441	0.2492	0.2403	0.2128	0.2762	0.54750001
40.6078	0.24330001	0.2483	0.24150001	0.21160001	0.27779999	0.54589999
40.75465	0.2441	0.24770001	0.23909999	0.21259999	0.27579999	0.54710001
40.9015333	0.24339999	0.24600001	0.2411	0.2133	0.27770001	0.54890001
41.0484	0.24259999	0.2448	0.2386	0.2114	0.2762	0.5474
41.1952667	0.2438	0.2471	0.2405	0.2105	0.2753	0.5467
41.3421333	0.2436	0.2448	0.2376	0.2123	0.27669999	0.54650003
41.489	0.24160001	0.2471	0.2362	0.2119	0.2771	0.54619998
41.63585	0.242	0.2466	0.23729999	0.2085	0.2746	0.54390001
41.9295833	0.2432	0.2472	0.2392	0.21080001	0.27509999	0.5478
42.0764333	0.2403	0.2464	0.2375	0.21080001	0.27559999	0.54710001
42.22335	0.2429	0.24690001	0.2396	0.2103	0.27399999	0.5456
42.3701667	0.2404	0.2466	0.2379	0.2093	0.273	0.54390001
42.51705	0.2402	0.2441	0.23720001	0.2087	0.2753	0.54570001
42.6639167	0.2431	0.2463	0.2384	0.2113	0.27540001	0.54619998
42.8107833	0.2414	0.2466	0.2367	0.20810001	0.27239999	0.5467
42.9576333	0.2402	0.2436	0.23639999	0.20990001	0.27599999	0.54619998
43.1045167	0.2406	0.2427	0.23459999	0.2115	0.27649999	0.54710001
43.39825	0.241	0.2448	0.2366	0.20999999	0.27489999	0.5474
43.5451	0.23980001	0.2457	0.2359	0.2085	0.2723	0.54290003
43.6919667	0.23999999	0.2428	0.2333	0.2102	0.27309999	0.54579997
43.8388667	0.2395	0.24429999	0.2367	0.2098	0.2766	0.54449999
43.9857	0.2386	0.2437	0.23270001	0.2093	0.27200001	0.54269999
44.1325667	0.2383	0.2446	0.2353	0.20810001	0.27039999	0.54519999
44.2794333	0.2402	0.244	0.2362	0.21070001	0.2746	0.54769999
44.4263	0.23999999	0.2436	0.23639999	0.20829999	0.2739	0.54650003
44.57315	0.241	0.2438	0.23819999	0.2085	0.27410001	0.54519999
44.8669	0.2384	0.2439	0.2335	0.20829999	0.2714	0.54530001
45.01375	0.2386	0.2428	0.234	0.2084	0.2719	0.546
45.1606167	0.23989999	0.24529999	0.2362	0.2087	0.2726	0.54519999
45.3074833	0.2366	0.2418	0.2349	0.2096	0.27540001	0.54610002
45.45435	0.2369	0.2422	0.2353	0.20900001	0.27289999	0.5474
45.6012167	0.23810001	0.2422	0.2358	0.2094	0.27320001	0.5438

45.7481	0.23980001	0.2441	0.2349	0.20999999	0.2744	0.54680002
45.89495	0.23890001	0.2418	0.2349	0.20909999	0.27239999	0.54369998
46.0418167	0.2379	0.24169999	0.2333	0.20720001	0.27149999	0.54339999
46.33555	0.2384	0.2427	0.2349	0.2067	0.26980001	0.54439998
46.4824	0.2385	0.2432	0.236	0.20829999	0.26989999	0.546
46.6292667	0.23649999	0.2423	0.23469999	0.20810001	0.2714	0.54570001
46.7761333	0.2366	0.2406	0.2344	0.20469999	0.2687	0.54390001
46.923	0.23710001	0.2392	0.2305	0.2062	0.26980001	0.5442
47.06985	0.2385	0.2438	0.2344	0.20990001	0.2728	0.54610002
47.2167167	0.2366	0.23999999	0.23360001	0.2084	0.2703	0.54299998
47.3635667	0.23639999	0.2395	0.2331	0.20630001	0.27000001	0.54470003
47.5104667	0.237	0.242	0.234	0.20720001	0.27070001	0.54430002
47.8042167	0.23630001	0.2404	0.2334	0.204	0.2678	0.54269999
47.9510667	0.237	0.24169999	0.23280001	0.20479999	0.27070001	0.5424
48.0981667	0.235	0.2393	0.2325	0.2068	0.27070001	0.54449999
48.2451167	0.237	0.24089999	0.2323	0.20739999	0.2683	0.54189998
48.3919833	0.2362	0.2404	0.2335	0.2067	0.27090001	0.54519999
48.53885	0.2358	0.2401	0.2352	0.2084	0.27149999	0.54500002
48.6857167	0.23559999	0.23890001	0.2318	0.2049	0.2692	0.54140002
48.8326	0.2359	0.23810001	0.2316	0.20649999	0.27039999	0.54409999
48.9794333	0.2361	0.2403	0.23190001	0.2066	0.2667	0.54320002
49.2731667	0.2351	0.2388	0.2315	0.20730001	0.271	0.54409999
49.4200333	0.2358	0.2387	0.23100001	0.20810001	0.26989999	0.54159999
49.5669	0.2358	0.23899999	0.2307	0.2059	0.26809999	0.53930002
49.7137667	0.2342	0.2397	0.2322	0.2051	0.26679999	0.54079998
49.8606167	0.23549999	0.23909999	0.2323	0.20630001	0.2696	0.54409999
50.0075167	0.23280001	0.2375	0.2314	0.2044	0.2674	0.54149997
50.1543667	0.2331	0.2388	0.2278	0.2057	0.26750001	0.54079998
50.3012167	0.23270001	0.2387	0.23019999	0.2079	0.27000001	0.54430002
50.4480833	0.23540001	0.2384	0.2317	0.204	0.2678	0.54049999
50.7418167	0.236	0.2401	0.23	0.2045	0.26820001	0.54119998
50.8886833	0.2335	0.23710001	0.2282	0.20550001	0.26820001	0.54409999
51.03555	0.2343	0.23810001	0.23029999	0.2043	0.26699999	0.54250002
51.1824	0.23370001	0.2406	0.2304	0.20200001	0.266	0.54089999
51.3293	0.2335	0.23800001	0.2305	0.205	0.2678	0.54180002
51.4761833	0.2332	0.2379	0.23109999	0.20479999	0.26730001	0.5406
51.6230167	0.235	0.2376	0.23019999	0.2034	0.2665	0.54140002
51.77005	0.2341	0.237	0.23109999	0.2054	0.26750001	0.5438
51.9169	0.23100001	0.23720001	0.2287	0.2054	0.26609999	0.5406
52.2108167	0.2323	0.2383	0.2289	0.20290001	0.2669	0.53909999
52.3576667	0.2331	0.2362	0.2272	0.20469999	0.2667	0.54170001
52.5045333	0.2322	0.23819999	0.2298	0.2045	0.26890001	0.54110003
52.6513833	0.2333	0.237	0.2289	0.2053	0.2669	0.54159999
52.7982667	0.2317	0.237	0.2286	0.20290001	0.26449999	0.54049999
52.94515	0.2333	0.2386	0.2287	0.205	0.26449999	0.54140002
53.092	0.2334	0.2369	0.22840001	0.2036	0.26640001	0.54049999

53.2388667	0.2342	0.23729999	0.2299	0.2036	0.26879999	0.54250002
53.3857167	0.23119999	0.23360001	0.22669999	0.2066	0.2683	0.54189998
53.67945	0.2321	0.2344	0.2288	0.20299999	0.2631	0.53930002
53.8263167	0.2308	0.23540001	0.22830001	0.2036	0.26199999	0.53789997
53.9731833	0.2339	0.2366	0.2289	0.2024	0.2633	0.53680003
54.1200333	0.2317	0.23649999	0.22759999	0.20290001	0.26699999	0.54189998
54.2669	0.2325	0.23649999	0.2274	0.2031	0.2649	0.53890002
54.4137667	0.2323	0.23649999	0.228	0.20379999	0.2615	0.53659999
54.56065	0.23119999	0.23549999	0.22830001	0.2045	0.2638	0.53860003
54.7075167	0.2297	0.23459999	0.22499999	0.2031	0.26550001	0.54189998
54.8543667	0.23100001	0.236	0.22660001	0.20200001	0.26289999	0.53829998
55.1481	0.2318	0.23459999	0.2282	0.2001	0.2633	0.53799999
55.2949667	0.2277	0.233	0.22679999	0.20119999	0.26339999	0.53839999
55.4418333	0.23119999	0.2333	0.2271	0.20119999	0.26460001	0.53750002
55.5886833	0.2296	0.23469999	0.2261	0.2042	0.26370001	0.5406
55.7357333	0.2304	0.2339	0.22669999	0.2026	0.26390001	0.53860003
55.8825833	0.2298	0.23370001	0.2278	0.204	0.26480001	0.54119998
56.0294667	0.2309	0.23450001	0.226	0.2023	0.2622	0.53649998
56.1763333	0.2309	0.2335	0.22499999	0.2044	0.2647	0.53860003
56.3232	0.22840001	0.23459999	0.2261	0.2045	0.26269999	0.5381
56.6169167	0.229	0.2324	0.2247	0.2016	0.26210001	0.53680003
56.7637667	0.2288	0.23469999	0.2278	0.205	0.26699999	0.54030001
56.91065	0.23	0.23459999	0.2252	0.2008	0.26289999	0.54009998
57.0575	0.2279	0.23280001	0.22480001	0.2042	0.26199999	0.53899997
57.2045333	0.22939999	0.2331	0.22319999	0.20119999	0.26179999	0.54030001
57.3514167	0.2281	0.2334	0.2244	0.2022	0.2631	0.53799999
57.4982833	0.2289	0.2335	0.2255	0.2022	0.26210001	0.53789997
57.64515	0.2273	0.23289999	0.2247	0.2001	0.26199999	0.537
57.7920167	0.2282	0.23270001	0.2251	0.1996	0.26190001	0.53649998
58.0857333	0.2286	0.2322	0.2237	0.2025	0.26409999	0.53719997
58.2325833	0.227	0.2309	0.2235	0.2022	0.26190001	0.53909999
58.37945	0.2291	0.23119999	0.22589999	0.2016	0.25979999	0.53570002
58.5263333	0.2269	0.23270001	0.222	0.2016	0.26089999	0.53719997
58.6731833	0.2271	0.23280001	0.22570001	0.2015	0.26350001	0.53680003
58.82005	0.22840001	0.233	0.2237	0.1983	0.26069999	0.53579998
58.9669	0.2295	0.2313	0.2244	0.20110001	0.26159999	0.53740001
59.1137833	0.2274	0.2321	0.2246	0.204	0.26210001	0.53839999
59.26065	0.22939999	0.2332	0.2245	0.20299999	0.26159999	0.53839999
59.55455	0.22939999	0.2349	0.2264	0.2027	0.2626	0.54000002
59.7014167	0.22849999	0.2318	0.2253	0.20029999	0.26089999	0.5363
59.8482833	0.22669999	0.2317	0.2234	0.20299999	0.26339999	0.53670001
59.9951333	0.227	0.2296	0.2228	0.19939999	0.2613	0.53680003
60.1420167	0.22570001	0.2315	0.2202	0.2005	0.26109999	0.53619999
60.28885	0.22830001	0.23379999	0.2261	0.1978	0.26019999	0.53609997
60.4357333	0.22499999	0.2296	0.221	0.1998	0.26179999	0.53560001
60.5825833	0.2263	0.2309	0.2227	0.1996	0.26050001	0.53460002

60.7294833	0.22660001	0.2322	0.2226	0.1971	0.2581	0.53259999
61.0232	0.2263	0.2318	0.22220001	0.20209999	0.26179999	0.53729999
61.1700667	0.22669999	0.22920001	0.22220001	0.2018	0.26100001	0.53750002
61.3169333	0.2247	0.2288	0.2217	0.1999	0.2597	0.53759998
61.4637833	0.2263	0.2299	0.2247	0.2005	0.25870001	0.53430003
61.6106667	0.2273	0.23109999	0.22390001	0.2017	0.2617	0.53430003
61.7575167	0.2245	0.2291	0.21969999	0.2016	0.26069999	0.53560001
61.9043833	0.2261	0.2305	0.22220001	0.1992	0.26019999	0.53640002
62.05125	0.2269	0.2323	0.2235	0.1981	0.259	0.5345
62.1981	0.2272	0.22930001	0.2219	0.20020001	0.25909999	0.53530002
62.4920333	0.22400001	0.23	0.2221	0.1988	0.2579	0.53359997
62.6388833	0.2269	0.22849999	0.2225	0.19750001	0.25979999	0.53539997
62.78575	0.22400001	0.2297	0.22050001	0.19859999	0.2595	0.53469998
62.9326167	0.2262	0.2295	0.22239999	0.20100001	0.25940001	0.53680003
63.0794833	0.2235	0.2282	0.22050001	0.2009	0.26159999	0.53740001
63.22635	0.22400001	0.2271	0.21969999	0.20020001	0.26120001	0.53390002
63.3732	0.2265	0.23029999	0.22239999	0.2015	0.25920001	0.53549999
63.52005	0.22490001	0.22830001	0.22149999	0.2008	0.2606	0.53469998
63.6669333	0.226	0.2304	0.2207	0.19939999	0.25780001	0.53479999
63.96065	0.22319999	0.2321	0.2234	0.20100001	0.25960001	0.53579998
64.1075333	0.22480001	0.229	0.22139999	0.20110001	0.25819999	0.53469998
64.2544	0.2271	0.23019999	0.2226	0.1998	0.259	0.53609997
64.4012667	0.2235	0.2289	0.22059999	0.1996	0.25960001	0.53549999
64.5481333	0.2202	0.22589999	0.2193			
64.695	0.2244	0.2295	0.22149999			
64.84185	0.22490001	0.23019999	0.2219			
64.9887167	0.2235	0.2273	0.22139999			
64.9887167						

Mutant 446-44

	3			1		
0.73890001	0.62360001	0.76889998	0.7119	0.71259999	0.72320002	0.53359997
0.78009999	0.6613	0.72970003	0.7098	0.7105	0.72240001	0.6031
0.77600002	0.67690003	0.82669997	0.71130002	0.71039999	0.72409999	0.6178
0.77219999	0.70899999	0.73400003	0.71289998	0.71249998	0.71710002	0.62339997
0.7669	0.74690002	0.76050001	0.71100003	0.7112	0.71679997	0.62230003
0.75599998	0.75080001	0.7723	0.70770001	0.70990002	0.71929997	0.61769998
0.7586	0.745	0.74400002	0.70910001	0.71069998	0.71640003	0.61369997
0.75089997	0.74349999	0.7342	0.70819998	0.7112	0.71859998	0.61320001
0.7561	0.7306	0.77420002	0.70740002	0.70969999	0.71920002	0.61129999
0.75709999	0.7299	0.80470002	0.70880002	0.71069998	0.71780002	0.60970002
0.74769998	0.71799999	0.77749997	0.70590001	0.7076	0.71679997	0.60659999
0.76010001	0.72839999	0.76980001	0.70609999	0.70550001	0.71319997	0.6067
0.76499999	0.72210002	0.7227	0.7069	0.70810002	0.71600002	0.61449999
0.77579999	0.73449999	0.7906	0.70289999	0.70520002	0.71560001	0.61860001
0.77060002	0.7353	0.75779998	0.70539999	0.70829999	0.71179998	0.61629999
0.79100001	0.75510001	0.78210002	0.70499998	0.70639998	0.71420002	0.61909997
0.7773	0.7529	0.77939999	0.7044	0.7033	0.71179998	0.62099999
0.7809	0.77700001	0.7809	0.70459998	0.70539999	0.7148	0.62269998
0.78280002	0.7755	0.78719997	0.70529997	0.7062	0.71280003	0.62089998
0.77289999	0.77109998	0.7809	0.70450002	0.70499998	0.71249998	0.62339997
0.75760001	0.77340001	0.76239997	0.7015	0.70240003	0.71090001	0.62110001
0.77509999	0.75400001	0.77149999	0.7022	0.7062	0.7119	0.62690002
0.76160002	0.75370002	0.76910001	0.70300001	0.70319998	0.7123	0.62110001
0.76740003	0.74980003	0.75910002	0.70120001	0.70289999	0.70859998	0.62169999
0.77289999	0.75269997	0.77859998	0.7008	0.70300001	0.71399999	0.62309998
0.76660001	0.75559998	0.76499999	0.70359999	0.7026	0.71310002	0.62110001
0.76849997	0.76819998	0.7737	0.70359999	0.7026	0.71060002	0.62199998
0.76389998	0.75690001	0.78329998	0.70050001	0.70179999	0.70889997	0.62040001
0.76160002	0.75529999	0.75220001	0.70230001	0.70490003	0.71100003	0.62040001
0.78289998	0.75410002	0.7784	0.70200002	0.7026	0.71240002	0.6261
0.77020001	0.7511	0.78609997	0.70099998	0.7022	0.70959997	0.6189
0.75190002	0.75050002	0.77899998	0.69870001	0.70050001	0.71090001	0.61849999
0.76480001	0.74699998	0.77560002	0.70130002	0.7026	0.71109998	0.62080002
0.7608	0.7507	0.78869998	0.70109999	0.6997	0.70969999	0.61659998
0.6512	0.61659998	0.63840002	0.69840002	0.69980001	0.70840001	0.6171
0.61540002	0.57590002	0.59079999	0.62099999	0.63929999	0.5675	0.59369999
0.59189999	0.55540001	0.57819998	0.61210001	0.61119998	0.54089999	0.59170002
0.58099997	0.54579997	0.55919999	0.61290002	0.61489999	0.546	0.58350003
0.57480001	0.53469998	0.55589998	0.6027	0.60479999	0.54640001	0.57230002
0.56569999	0.52079999	0.54220003	0.61360002	0.61729997	0.55669999	0.56739998
0.55419999	0.51200002	0.5345	0.6182	0.6225	0.56489998	0.56599998
0.54089999	0.49610001	0.52209997	0.61750001	0.62949997	0.57300001	0.56559998
0.52679998	0.48930001	0.514	0.61900002	0.62949997	0.58029997	0.5668
0.51679999	0.47940001	0.5115	0.62550002	0.63679999	0.58459997	0.56819999

0.50840002	0.4707	0.4948	0.62800002	0.64050001	0.59189999	0.56690001
0.4939	0.4648	0.49219999	0.63099998	0.63840002	0.5966	0.5614
0.4878	0.45879999	0.4831	0.6336	0.6433	0.60329998	0.56349999
0.48339999	0.45289999	0.4689	0.64270002	0.64780003	0.61119998	0.56129998
0.4725	0.43900001	0.46759999	0.63980001	0.64719999	0.6171	0.56519997
0.4668	0.43529999	0.45649999	0.64560002	0.64810002	0.62099999	0.56489998
0.46129999	0.43020001	0.44839999	0.6397	0.6523	0.61900002	0.56190002
0.4465	0.42770001	0.4456	0.6473	0.65530002	0.62870002	0.55989999
0.44319999	0.41949999	0.44010001	0.64270002	0.65270001	0.62879997	0.56089997
0.4402	0.4172	0.43270001	0.64249998	0.65880001	0.63160002	0.56269997
0.43759999	0.41440001	0.42820001	0.64880002	0.65789998	0.63380003	0.56230003
0.4321	0.4073	0.42140001	0.64990002	0.65850002	0.63520002	0.5582
0.43040001	0.4034	0.42070001	0.65149999	0.65869999	0.63929999	0.56089997
0.4249	0.40279999	0.41440001	0.65060002	0.65259999	0.63749999	0.55800003
0.42019999	0.39520001	0.4084	0.64859998	0.65750003	0.63810003	0.56059998
0.4183	0.39410001	0.40400001	0.65219998	0.65490001	0.63660002	0.55949998
0.41010001	0.39120001	0.40650001	0.65140003	0.65460002	0.63810003	0.55610001
0.4102	0.39019999	0.40689999	0.65289998	0.65630001	0.63950002	0.55549997
0.40580001	0.38589999	0.39910001	0.6498	0.65460002	0.63840002	0.55900002
0.40470001	0.38800001	0.40059999	0.65090001	0.65329999	0.63910002	0.5575
0.39860001	0.3822	0.39469999	0.65390003	0.65270001	0.63700002	0.55720001
0.39179999	0.37760001	0.38929999	0.64920002	0.65060002	0.63660002	0.5546
0.39019999	0.37760001	0.38710001	0.65179998	0.65140003	0.63569999	0.55610001
0.3872	0.37560001	0.38609999	0.64499998	0.6494	0.63599998	0.55739999
0.38299999	0.3716	0.38049999	0.64660001	0.65499997	0.63380003	0.55559999
0.37959999	0.37059999	0.37819999	0.64899999	0.65259999	0.63319999	0.55290002
0.37400001	0.36539999	0.37360001	0.64889997	0.64880002	0.63520002	0.55729997
0.36939999	0.3626	0.37099999	0.64359999	0.6505	0.63440001	0.55309999
0.36880001	0.3637	0.3707	0.64219999	0.64889997	0.63370001	0.55699998
0.36590001	0.36250001	0.36759999	0.64780003	0.65170002	0.63499999	0.55690002
0.36050001	0.36050001	0.3642	0.6433	0.65090001	0.63270003	0.5539
0.36059999	0.3558	0.36160001	0.64529997	0.64850003	0.63300002	0.55760002
0.35949999	0.3506	0.35960001	0.64279997	0.64420003	0.63440001	0.55400002
0.35479999	0.35139999	0.3563	0.64069998	0.64700001	0.6336	0.55489999
0.3545	0.3477	0.3538	0.64380002	0.64429998	0.63370001	0.55479997
0.3522	0.3452	0.35010001	0.64069998	0.64700001	0.63340002	0.55430001
0.34740001	0.34619999	0.35069999	0.64319998	0.64740002	0.63059998	0.55199999
0.347	0.3373	0.3436	0.6426	0.64679998	0.6347	0.55489999
0.34760001	0.33939999	0.34450001	0.63779998	0.64399999	0.63020003	0.55010003
0.34380001	0.33750001	0.3423	0.63349998	0.64020002	0.63059998	0.55519998
0.34380001	0.33520001	0.3396	0.64240003	0.64649999	0.6347	0.55290002
0.34169999	0.33489999	0.33719999	0.6415	0.64829999	0.63160002	0.55089998
0.34079999	0.33239999	0.33340001	0.64170003	0.64990002	0.63440001	0.55180001
0.33989999	0.329	0.3301	0.63690001	0.64749998	0.63099998	0.54839998
0.34099999	0.32800001	0.3299	0.63330001	0.64090002	0.62849998	0.5517
0.33649999	0.3265	0.33000001	0.63550001	0.6415	0.6286	0.55019999

0.3346	0.32390001	0.32699999	0.63550001	0.6408	0.62959999	0.55339998
0.3339	0.32460001	0.32550001	0.63529998	0.6426	0.62970001	0.55150002
0.33019999	0.3202	0.3242	0.63599998	0.64109999	0.62989998	0.54879999
0.33199999	0.32089999	0.3231	0.63770002	0.64389998	0.62720001	0.54729998
0.3301	0.3193	0.31990001	0.63249999	0.63980001	0.62760001	0.5517
0.32949999	0.31560001	0.31900001	0.63160002	0.63739997	0.6293	0.54820001
0.32600001	0.31560001	0.32049999	0.63230002	0.63749999	0.62540001	0.55140001
0.322	0.3152	0.3161	0.63459998	0.64249998	0.62699997	0.55229998
0.3204	0.31459999	0.31740001	0.63569999	0.63980001	0.62830001	0.54970002
0.3211	0.31079999	0.31760001	0.63200003	0.64039999	0.62589997	0.54879999
0.31799999	0.31150001	0.31779999	0.63389999	0.63919997	0.62589997	0.54979998
0.31920001	0.31209999	0.31330001	0.62900001	0.63889998	0.62720001	0.5535
0.31659999	0.30919999	0.3123	0.63370001	0.6408	0.63249999	0.55080003
0.31900001	0.3064	0.31040001	0.63050002	0.63620001	0.6275	0.54869998
0.31650001	0.30579999	0.3098	0.63370001	0.64069998	0.6286	0.54949999
0.31279999	0.30559999	0.3091	0.63200003	0.63870001	0.6275	0.55129999
0.31020001	0.3046	0.30840001	0.63389999	0.63770002	0.62699997	0.54769999
0.3109	0.30360001	0.30149999	0.6286	0.63550001	0.62580001	0.55070001
0.31330001	0.30360001	0.301	0.63010001	0.63959998	0.62580001	0.54930001
0.31150001	0.30230001	0.30160001	0.62910002	0.63679999	0.62809998	0.55080003
0.3107	0.30140001	0.3035	0.62809998	0.63639998	0.62419999	0.55040002
0.31200001	0.3021	0.30469999	0.63020003	0.63889998	0.625	0.54909998
0.3071	0.2999	0.30180001	0.62870002	0.63669997	0.62370002	0.54879999
0.3073	0.29890001	0.29840001	0.62639999	0.63330001	0.62620002	0.54769999
0.30509999	0.3001	0.2956	0.62910002	0.63779998	0.62559998	0.55049998
0.3037	0.29719999	0.2976	0.62629998	0.63739997	0.62419999	0.54979998
0.3057	0.29249999	0.2965	0.62760001	0.63599998	0.62629998	0.54790002
0.30540001	0.29660001	0.29820001	0.62840003	0.63880002	0.62709999	0.54689997
0.30239999	0.296	0.29350001	0.62819999	0.63429999	0.62279999	0.55089998
0.30360001	0.2929	0.29539999	0.62529999	0.63239998	0.62519997	0.54659998
0.3021	0.29350001	0.29229999	0.62669998	0.63789999	0.62330002	0.54640001
0.30090001	0.29300001	0.29319999	0.62699997	0.63319999	0.62120003	0.54970002
0.29899999	0.2929	0.29409999	0.62639999	0.63380003	0.62400001	0.54759997
0.30070001	0.29190001	0.29080001	0.6257	0.6347	0.62400001	0.54699999
0.29589999	0.29069999	0.2877	0.62449998	0.63059998	0.62040001	0.54750001
0.29370001	0.28979999	0.289	0.6239	0.63459998	0.62300003	0.54640001
0.29339999	0.2888	0.2881	0.62510002	0.62959999	0.62290001	0.5499
0.2942	0.2877	0.2904	0.62639999	0.6354	0.62269998	0.54809999
0.2897	0.28650001	0.28870001	0.62159997	0.62970001	0.62449998	0.54900002
0.29179999	0.28529999	0.28510001	0.6214	0.63279998	0.61919999	0.5456
0.29280001	0.2825	0.287	0.62629998	0.63270003	0.62159997	0.54830003
0.29069999	0.28439999	0.28380001	0.6232	0.62980002	0.6196	0.54769999
0.29030001	0.2825	0.2807	0.62230003	0.62900001	0.6207	0.54659998
0.28729999	0.28119999	0.285	0.62330002	0.63129997	0.62129998	0.54839998
0.28749999	0.2809	0.28060001	0.62150002	0.6293	0.61879998	0.54530001
0.28659999	0.27869999	0.2798	0.6243	0.63440001	0.6214	0.5503

0.29100001	0.28200001	0.28130001	0.62349999	0.63190001	0.61930001	0.54689997
0.28670001	0.28099999	0.27959999	0.61879998	0.62919998	0.62	0.54699999
0.2913	0.2802	0.2753	0.62019998	0.62900001	0.62010002	0.5474
0.2913	0.27680001	0.27810001	0.6225	0.6293	0.61900002	0.54549998
0.2879	0.2773	0.2735	0.62150002	0.62779999	0.61799997	0.54509997
0.2843	0.27829999	0.27489999	0.62370002	0.63029999	0.61900002	0.5438
0.28639999	0.27630001	0.27900001	0.62029999	0.62849998	0.61849999	0.54830003
0.2832	0.2775	0.27340001	0.61989999	0.6268	0.61760002	0.5442
0.28099999	0.27320001	0.27250001	0.62040001	0.63249999	0.62029999	0.5492
0.2825	0.2735	0.2757	0.62059999	0.62669998	0.61720002	0.54650003
0.28200001	0.27360001	0.27680001	0.61919999	0.62620002	0.61760002	0.54750001
0.2841	0.27689999	0.27289999	0.61879998	0.625	0.61750001	0.54710001
0.28259999	0.27630001	0.27559999	0.61720002	0.62599999	0.61799997	0.54680002
0.2852	0.27630001	0.27410001	0.61739999	0.62540001	0.61699998	0.5492
0.27919999	0.27110001	0.27079999	0.62099999	0.62830001	0.616	0.54720002
0.2798	0.27239999	0.26980001	0.61680001	0.62360001	0.6146	0.54460001
0.28009999	0.2714	0.27129999	0.6164	0.6232	0.6189	0.54720002
0.28299999	0.273	0.27090001	0.62080002	0.62690002	0.6196	0.55000001
0.2816	0.27129999	0.27329999	0.61760002	0.62639999	0.61479998	0.54549998
0.2816	0.26949999	0.27070001	0.61650002	0.62550002	0.61559999	0.5474
0.28060001	0.27070001	0.27160001	0.61610001	0.62180001	0.61570001	0.54610002
0.28299999	0.27169999	0.26910001	0.6146	0.62440002	0.61440003	0.54509997
0.278	0.2703	0.26969999	0.61909997	0.62620002	0.6164	0.54860002
0.2775	0.26769999	0.26679999	0.6189	0.62510002	0.61769998	0.54759997
0.2762	0.26859999	0.26710001	0.6164	0.62330002	0.61629999	0.54629999
0.2814	0.2649	0.2651	0.61260003	0.62370002	0.61690003	0.55000001
0.27540001	0.26539999	0.26589999	0.61449999	0.62110001	0.61470002	0.5478
0.27720001	0.26660001	0.2651	0.61360002	0.62330002	0.61309999	0.54549998
0.2793	0.26699999	0.26550001	0.61400002	0.62290001	0.61400002	0.54860002
0.27630001	0.26699999	0.2651	0.61330003	0.62269998	0.61269999	0.54449999
0.27309999	0.26449999	0.26550001	0.61549997	0.62360001	0.61440003	0.54619998
0.27379999	0.26699999	0.26710001	0.61430001	0.62080002	0.6128	0.54710001
0.2748	0.26429999	0.26460001	0.61430001	0.6232	0.6135	0.54960001
0.27239999	0.26370001	0.26499999	0.61180001	0.62260002	0.61269999	0.54830003
0.27430001	0.26519999	0.26480001	0.61400002	0.62089998	0.61260003	0.54369998
0.2685	0.2626	0.2626	0.6103	0.61949998	0.61220002	0.54500002
0.27070001	0.2658	0.2647	0.61379999	0.62360001	0.61449999	0.5478
0.26840001	0.26179999	0.26100001	0.61320001	0.62059999	0.61290002	0.54769999
0.26789999	0.26199999	0.26100001	0.61299998	0.62169999	0.6142	0.54430002
0.2685	0.2599	0.26120001	0.6124	0.62040001	0.61250001	0.5474
0.26800001	0.25999999	0.25870001	0.61089998	0.61930001	0.61299998	0.54390001
0.2694	0.25979999	0.25940001	0.61159998	0.62029999	0.61189997	0.54460001
0.2687	0.2608	0.26249999	0.61440003	0.62110001	0.61210001	0.54500002
0.26730001	0.25940001	0.25979999	0.61440003	0.62099999	0.61519998	0.54890001
0.27000001	0.2613	0.25839999	0.61360002	0.61930001	0.61440003	0.5492
0.26910001	0.26140001	0.25920001	0.61049998	0.61790001	0.61140001	0.5442

0.2678	0.2595	0.25749999	0.61040002	0.61879998	0.60900003	0.546
0.27329999	0.26010001	0.2597	0.61210001	0.62019998	0.61220002	0.54629999
0.26809999	0.25979999	0.25740001	0.6117	0.61769998	0.611	0.5449
0.26800001	0.25780001	0.25940001	0.61470002	0.61909997	0.61379999	0.54350001
0.2676	0.2581	0.26010001	0.61119998	0.61720002	0.61070001	0.5438
0.26989999	0.25830001	0.2579	0.60799998	0.62080002	0.61229998	0.54390001
0.2658	0.25549999	0.25619999	0.61210001	0.62230003	0.61150002	0.54699999
0.264	0.25780001	0.25799999	0.61210001	0.61909997	0.61269999	0.54400003
0.26320001	0.25510001	0.25459999	0.60890001	0.61799997	0.60820001	0.5449
0.26609999	0.2586	0.25690001	0.61119998	0.61699998	0.61040002	0.54220003
0.2649	0.26499999	0.25690001	0.60860002	0.61799997	0.6081	0.54470003
0.2622	0.25569999	0.2561	0.6092	0.61690003	0.61000001	0.54030001
0.26550001	0.25830001	0.25819999	0.60930002	0.61570001	0.61080003	0.54269999
0.26370001	0.252	0.2538	0.60790002	0.61729997	0.61220002	0.54259998
0.26440001	0.2559	0.255	0.61000001	0.61729997	0.61009997	0.54320002
0.26359999	0.2579	0.2559	0.60820001	0.61720002	0.61040002	0.54519999
0.26089999	0.2613	0.25279999	0.61140001	0.61750001	0.60970002	0.54259998
0.26339999	0.2586	0.25440001	0.60900003	0.61500001	0.60860002	0.54170001
0.26179999	0.25420001	0.25490001	0.60680002	0.61589998	0.60750002	0.54280001
0.2608	0.25479999	0.25310001	0.6063	0.61580002	0.60689998	0.54269999
0.26289999	0.25659999	0.2543	0.60729998	0.61500001	0.60680002	0.54280001
0.2615	0.2529	0.2518	0.60839999	0.61479998	0.60909998	0.54509997
0.25839999	0.25240001	0.25240001	0.6074	0.6142	0.60829997	0.54369998
0.26350001	0.25529999	0.2543	0.6067	0.61650002	0.6049	0.54329997
0.25889999	0.25330001	0.2536	0.60869998	0.61540002	0.60799998	0.54519999
0.2586	0.25709999	0.25260001	0.60780001	0.61870003	0.60890001	0.54339999
0.2568	0.25659999	0.2536	0.60650003	0.61360002	0.60869998	0.54049999
0.25780001	0.25299999	0.2491	0.60650003	0.61360002	0.60530001	0.54299998
0.25709999	0.2525	0.25060001	0.6081	0.61449999	0.60689998	0.5438
0.26109999	0.25389999	0.25060001	0.60479999	0.61119998	0.6081	0.5438
0.2597	0.2518	0.24959999	0.60579997	0.6124	0.6063	0.54390001
0.25889999	0.25209999	0.2502	0.60500002	0.61189997	0.60659999	0.5399
0.25549999	0.25319999	0.25150001	0.6063	0.61229998	0.60640001	0.54460001
0.25639999	0.25009999	0.25009999	0.6045	0.61260003	0.60610002	0.54040003
0.25760001	0.252	0.25080001	0.60509998	0.61229998	0.60570002	0.54180002
0.25619999	0.248	0.2518	0.6045	0.61140001	0.60659999	0.54290003
0.2581	0.2485	0.2474	0.60409999	0.61330003	0.60640001	0.54269999
0.25830001	0.2467	0.2467	0.6024	0.60890001	0.60470003	0.54170001
0.2561	0.24770001	0.2498	0.60280001	0.6117	0.60399997	0.54250002
0.2545	0.2458	0.2492	0.60280001	0.61180001	0.60509998	0.54189998
0.25490001	0.2465	0.2475	0.60299999	0.60939997	0.60250002	0.54409999
0.25350001	0.2476	0.2449	0.60399997	0.61040002	0.60479999	0.5438
0.2604	0.2476	0.2449	0.60280001	0.61150002	0.60619998	0.54210001
0.25279999	0.24779999	0.2456	0.60299999	0.61000001	0.6027	0.54110003
0.2561	0.24879999	0.2476	0.60149997	0.60900003	0.60360003	0.54360002
0.25220001	0.245	0.2458	0.6027	0.60829997	0.60540003	0.54189998

0.25119999	0.2465	0.2449	0.60430002	0.61150002	0.60540003	0.54229999
0.25420001	0.2462	0.2467	0.60299999	0.61000001	0.60329998	0.54189998
0.25619999	0.2482	0.24770001	0.59920001	0.60979998	0.6024	0.5388
0.2527	0.2447	0.2464	0.60089999	0.60519999	0.60100001	0.54220003
0.2538	0.2455	0.2457	0.60219997	0.6085	0.60329998	0.5388
0.25040001	0.2438	0.2447	0.6013	0.60960001	0.60170001	0.54470003
0.25330001	0.2465	0.2483	0.59899998	0.60619998	0.59979999	0.53890002
0.25490001	0.24699999	0.2463	0.60049999	0.60949999	0.60140002	0.54269999
0.25	0.2427	0.2423	0.60180002	0.6092	0.60000002	0.54170001
0.2511	0.2445	0.24510001	0.5988	0.6045	0.60110003	0.54259998
0.25299999	0.2464	0.24519999	0.59909999	0.60759997	0.60210001	0.54089999
0.2536	0.24590001	0.243	0.60110003	0.60869998	0.60219997	0.54119998
0.25009999	0.24420001	0.2412	0.60009998	0.60710001	0.60259998	0.54360002
0.249	0.24330001	0.2428	0.60210001	0.60829997	0.602	0.54070002
0.25560001	0.24330001	0.24339999	0.60089999	0.60600001	0.60399997	0.54350001
0.25	0.24259999	0.2412	0.59829998	0.6063	0.60100001	0.5413
0.2499	0.2404	0.244	0.60079998	0.60750002	0.602	0.54299998
0.2499	0.24070001	0.2427	0.59719998	0.60500002	0.60140002	0.54329997
0.2499	0.23890001	0.2418	0.59859997	0.60890001	0.60149997	0.54229999
0.24950001	0.2437	0.2431	0.59850001	0.60659999	0.60049999	0.54280001
0.25139999	0.242	0.243	0.59920001	0.6045	0.6002	0.54180002
0.2516	0.24240001	0.243	0.5988	0.60439998	0.60089999	0.54049999
0.24779999	0.23899999	0.24150001	0.5977	0.60699999	0.59820002	0.5449
0.24869999	0.2394	0.2427	0.59729999	0.6045	0.6013	0.5399
0.2448	0.2414	0.23989999	0.59810001	0.60399997	0.60089999	0.54159999
0.2473	0.2386	0.2431	0.59619999	0.60280001	0.597	0.54320002
0.25229999	0.2454	0.2436	0.59829998	0.6063	0.60009998	0.54299998
0.24779999	0.24079999	0.2388	0.59670001	0.60610002	0.60000002	0.53969997
0.25139999	0.242	0.2428	0.59619999	0.60530001	0.59420002	0.54049999
0.2476	0.24240001	0.2414	0.59789997	0.60460001	0.59689999	0.54030001
0.2446	0.2386	0.23909999	0.59750003	0.6031	0.59689999	0.54290003
0.24240001	0.23989999	0.23710001	0.59960002	0.60339999	0.60079998	0.5424
0.24959999	0.24070001	0.2418	0.59799999	0.6045	0.59960002	0.54000002
0.2494	0.24169999	0.2419	0.59670001	0.6049	0.59789997	0.54089999
0.2491	0.2392	0.23989999	0.59640002	0.6049	0.5977	0.54220003
0.2474	0.2414	0.24160001	0.597	0.60259998	0.59600002	0.54049999
0.2456	0.23819999	0.24150001	0.59820002	0.6049	0.59799999	0.54049999
0.24779999	0.2406	0.23980001	0.59869999	0.6045	0.59920001	0.5406
0.2473	0.2406	0.23980001	0.59350002	0.60329998	0.59979999	0.53799999
0.2432	0.23729999	0.2358	0.59780002	0.6031	0.59719998	0.53759998
0.2418	0.23540001	0.2342	0.59740001	0.60470003	0.59789997	0.54280001
0.2439	0.23909999	0.2388	0.5952	0.60119998	0.59670001	0.54040003
0.2483	0.2405	0.23810001	0.59710002	0.6045	0.59689999	0.53899997
0.2436	0.2388	0.2361	0.5959	0.60180002	0.59670001	0.5431
0.243	0.2374	0.235	0.59689999	0.60000002	0.59710002	0.54460001
0.245	0.2405	0.23810001	0.59490001	0.602	0.59630001	0.54119998

0.2466	0.2384	0.2386	0.59320003	0.6027	0.59500003	0.542
0.2473	0.23890001	0.2361	0.5927	0.59810001	0.59189999	0.5406
0.2464	0.23559999	0.2374	0.5934	0.5995	0.59390002	0.54110003
0.24779999	0.23639999	0.237	0.59600002	0.6013	0.59509999	0.54210001
0.2493	0.23909999	0.23630001	0.59490001	0.60100001	0.59399998	0.54400003
0.2462	0.23540001	0.2335	0.59619999	0.60210001	0.59649998	0.5402
0.24869999	0.237	0.2369	0.59189999	0.59799999	0.59429997	0.54049999
0.24609999	0.23729999	0.23800001	0.59500003	0.59969997	0.59469998	0.54140002
0.2465	0.237	0.2378	0.59289998	0.6006	0.59570003	0.54250002
0.24439999	0.2353	0.2359	0.59530002	0.60170001	0.5959	0.5424
0.2463	0.235	0.2374	0.5923	0.59920001	0.59280002	0.54170001
0.2509	0.237	0.2378	0.59460002	0.59939998	0.59579998	0.54119998
0.2472	0.2362	0.23989999	0.59420002	0.5995	0.59359998	0.54390001
0.23909999	0.23630001	0.2369	0.59429997	0.60000002	0.59429997	0.53920001
0.2432	0.23819999	0.23999999	0.59390002	0.60119998	0.59509999	0.53930002
0.24860001	0.2359	0.23710001	0.59649998	0.60079998	0.59380001	0.53829998
0.24959999	0.23450001	0.2367	0.59469998	0.60009998	0.59399998	0.54119998
0.2446	0.2321	0.235	0.59210002	0.59799999	0.59259999	0.54170001
0.243	0.235	0.2358	0.59420002	0.59979999	0.5941	0.53969997
0.2464	0.2351	0.2353	0.59259999	0.59689999	0.59369999	0.54149997
0.2474	0.2361	0.2374	0.59210002	0.59609997	0.59100002	0.54299998
0.2481	0.23289999	0.2332	0.59259999	0.59789997	0.59210002	0.53780001
0.24439999	0.23469999	0.2358	0.59450001	0.5952	0.59350002	0.542
0.2474	0.235	0.2357	0.59210002	0.5977	0.5941	0.54210001
0.24519999	0.23109999	0.23190001	0.59170002	0.59789997	0.59469998	0.53969997
0.24510001	0.2323	0.2323	0.59210002	0.59820002	0.59210002	0.53979999
0.2431	0.2316	0.2307	0.58969998	0.59630001	0.5916	0.5413
0.24590001	0.2339	0.23010001	0.5905	0.59609997	0.59310001	0.53930002
0.2429	0.2322	0.2332	0.58990002	0.59719998	0.59200001	0.5395
0.2474	0.23549999	0.2335	0.59119999	0.59630001	0.58990002	0.542
0.2414	0.2321	0.2324	0.58990002	0.59500003	0.58939999	0.54149997
0.2448	0.2343	0.23100001	0.59030002	0.59600002	0.59259999	0.54280001
0.23899999	0.23270001	0.2304	0.5887	0.59240001	0.59009999	0.53979999
0.2405	0.23119999	0.2316	0.58829999	0.5941	0.59079999	0.54299998
0.24519999	0.2335	0.2344	0.59179997	0.59570003	0.59210002	0.54009998
0.248	0.23540001	0.2315	0.58700001	0.59280002	0.58890003	0.5402
0.2476	0.235	0.23199999	0.59109998	0.59460002	0.58950001	0.53869998
0.2397	0.2331	0.2287	0.58899999	0.59280002	0.59060001	0.54339999
0.2418	0.23540001	0.23190001	0.58840001	0.5948	0.58920002	0.53930002
0.24259999	0.2353	0.2342	0.58969998	0.59359998	0.59119999	0.54189998
0.24079999	0.23119999	0.22920001	0.58829999	0.59509999	0.59060001	0.5406
0.24429999	0.2324	0.2296	0.58710003	0.59259999	0.58810002	0.54259998
0.2456	0.2335	0.2324	0.59069997	0.59469998	0.59030002	0.54049999
0.24590001	0.23370001	0.2344	0.58569998	0.59219998	0.58950001	0.54110003
0.24070001	0.2304	0.23010001	0.58859998	0.59240001	0.58789998	0.54110003
0.2419	0.2297	0.23190001	0.58899999	0.59140003	0.58920002	0.54000002

0.2384	0.23029999	0.23289999	0.58840001	0.59320003	0.58960003	0.54589999
0.24240001	0.2317	0.23199999	0.58850002	0.59329998	0.58819997	0.542
0.2427	0.2298	0.2306	0.58740002	0.59170002	0.58960003	0.53960001
0.23810001	0.2286	0.2296	0.5891	0.59149998	0.58819997	0.54229999
0.236	0.2305	0.2316	0.5887	0.59350002	0.58969998	0.5402
0.2367	0.2313	0.23199999	0.58819997	0.59179997	0.58890003	0.54180002
0.2384	0.23	0.2309	0.58700001	0.59200001	0.58789998	0.53829998
0.2368	0.2298	0.2309	0.59030002	0.5905	0.58810002	0.54049999
0.24150001	0.2295	0.2309	0.58660001	0.59179997	0.58719999	0.54400003
0.24439999	0.2273	0.2318	0.58389997	0.59009999	0.58810002	0.54070002
0.2388	0.23100001	0.2307	0.58600003	0.59149998	0.58810002	0.53789997
0.2374	0.2306	0.2313	0.58780003	0.59289998	0.5873	0.54110003
0.2431	0.2309	0.23029999	0.58710003	0.59189999	0.58740002	0.53710002
0.2411	0.2298	0.22930001	0.58719999	0.59200001	0.58700001	0.542
0.2396	0.23190001	0.2287	0.58649999	0.59079999	0.58560002	0.54189998
0.23710001	0.2282	0.2295	0.58840001	0.59249997	0.58810002	0.54189998
0.2422	0.23	0.2295	0.5873	0.59320003	0.58939999	0.54140002
0.2392	0.2321	0.2309	0.58850002	0.59259999	0.58789998	0.5413
0.2402	0.22849999	0.2305	0.58670002	0.58960003	0.58670002	0.53860003
0.2392	0.22750001	0.2321	0.58679998	0.59030002	0.58279997	0.54250002
0.2396	0.229	0.2277	0.5862	0.59060001	0.58520001	0.53920001
0.23639999	0.2288	0.2272	0.58459997	0.59030002	0.58639997	0.54140002
0.2379	0.22849999	0.22750001	0.5862	0.59009999	0.58539999	0.53710002
0.24349999	0.2296	0.229	0.58520001	0.58789998	0.58600003	0.53920001
0.2422	0.22830001	0.2287	0.58350003	0.59039998	0.58459997	0.53960001
0.2376	0.2279	0.22830001	0.58789998	0.59189999	0.58749998	0.5406
0.234	0.2299	0.22840001	0.5851	0.58929998	0.58249998	0.5399
0.2383	0.22920001	0.22939999	0.58279997	0.58950001	0.57920003	0.53969997
0.23710001	0.2263	0.22750001	0.58649999	0.58840001	0.58459997	0.54040003
0.23720001	0.2287	0.2288	0.5869	0.58899999	0.5851	0.53979999
0.23810001	0.2281	0.2299	0.58469999	0.58859998	0.58230001	0.53820002
0.23370001	0.2296	0.2273	0.58319998	0.58819997	0.58389997	0.5402
0.2414	0.2297	0.228	0.58459997	0.58679998	0.5837	0.53979999
0.2396	0.2265	0.2272	0.58539999	0.58829999	0.58459997	0.53820002
0.23639999	0.2274	0.2255	0.58230001	0.58639997	0.58399999	0.53789997
0.2378	0.2287	0.22669999	0.58450001	0.5887	0.58279997	0.54040003
0.2358	0.2254	0.2261	0.583	0.58770001	0.58249998	0.53860003
0.2359	0.2261	0.22669999	0.58289999	0.5891	0.583	0.54079998
0.23469999	0.22660001	0.2252	0.5826	0.58329999	0.57990003	0.5399
0.23540001	0.22840001	0.2289	0.5855	0.58639997	0.58149999	0.53890002
0.233	0.2289	0.2289	0.5826	0.58530003	0.58099997	0.5402
0.23899999	0.22589999	0.2247	0.5848	0.58660001	0.58109999	0.53780001
0.23810001	0.2277	0.2272	0.58240002	0.58719999	0.583	0.5395
0.23639999	0.2282	0.22830001	0.57980001	0.58469999	0.58240002	0.53850001
0.2383	0.2282	0.2272	0.58170003	0.58700001	0.58170003	0.5413
0.2333	0.22589999	0.2254	0.58459997	0.58679998	0.58219999	0.53890002

0.23729999	0.22759999	0.2277	0.58279997	0.58609998	0.58029997	0.53829998
0.23649999	0.22750001	0.2277	0.58039999	0.58560002	0.58130002	0.54119998
0.236	0.2262	0.2262	0.58060002	0.58469999	0.58029997	0.5399
0.23450001	0.22830001	0.226	0.57959998	0.58380002	0.57999998	0.5363
0.23190001	0.22669999	0.22490001	0.58160001	0.58630002	0.58099997	0.53969997
0.2402	0.22830001	0.2255	0.58060002	0.58350003	0.5819	0.53969997
0.24150001	0.22669999	0.22679999	0.5819	0.58579999	0.5819	0.5395
0.236	0.2238	0.2217	0.58139998	0.5844	0.58289999	0.53649998
0.24240001	0.229	0.22750001	0.58090001	0.5862	0.57800001	0.53839999
0.234	0.2263	0.2247	0.58099997	0.58170003	0.57959998	0.54170001
0.23469999	0.22589999	0.2282	0.58090001	0.58319998	0.58149999	0.53570002
0.23360001	0.2228	0.22400001	0.57969999	0.58569998	0.58170003	0.54040003
0.2322	0.2234	0.22139999	0.57620001	0.58200002	0.57929999	0.53710002
0.235	0.2245	0.2289	0.5819	0.58319998	0.58090001	0.53640002
0.2349	0.2261	0.22589999	0.5808	0.58490002	0.57950002	0.53909999
0.23710001	0.2236	0.2274	0.57969999	0.58319998	0.58020002	0.5399
0.2333	0.2235	0.2277	0.58039999	0.58200002	0.57980001	0.54089999
0.2332	0.2246	0.2263	0.5794	0.58350003	0.58029997	0.53560001
0.2322	0.2253	0.22589999	0.58069998	0.5844	0.57660002	0.53789997
0.23630001	0.22660001	0.2273	0.57849997	0.58469999	0.57840002	0.5402
0.23450001	0.22319999	0.223	0.57859999	0.58060002	0.57950002	0.53759998
0.2321	0.22130001	0.2235	0.57819998	0.58139998	0.58020002	0.54119998
0.2378	0.2244	0.2254	0.5794	0.58310002	0.57669997	0.53600001
0.2314	0.2235	0.22310001	0.57770002	0.58099997	0.5783	0.53789997
0.2317	0.2242	0.22589999	0.57730001	0.5812	0.57679999	0.54070002
0.23289999	0.22400001	0.2264	0.57789999	0.57969999	0.57800001	0.537
0.2334	0.22390001	0.226	0.57639998	0.58060002	0.57859999	0.5388
0.23019999	0.2237	0.22679999	0.5783	0.58090001	0.57660002	0.53860003
0.2341	0.2252	0.22920001	0.57709998	0.57969999	0.57849997	0.53530002
0.2369	0.22139999	0.22480001	0.5765	0.58050001	0.57639998	0.53869998
0.2368	0.2207	0.2252	0.57770002	0.57980001	0.57609999	0.53899997
0.2333	0.2211	0.2246	0.57749999	0.57929999	0.57709998	0.5352
0.2271	0.2245	0.2271	0.57789999	0.57980001	0.5765	0.53839999
0.233	0.2216	0.2277	0.57730001	0.57959998	0.57669997	0.53920001
0.2308	0.2251	0.2253	0.579	0.57929999	0.57770002	0.53670001
0.2277	0.22229999	0.22660001	0.57859999	0.58209997	0.57669997	0.53789997
0.2261	0.2218	0.22390001	0.57969999	0.57800001	0.57730001	0.53930002
0.2296	0.2212	0.2263	0.57709998	0.5801	0.5765	0.53960001
0.2289	0.2212	0.2245	0.5783	0.58069998	0.57770002	0.5413
0.2304	0.2235	0.2282	0.57679999	0.58130002	0.57669997	0.53719997
0.227	0.2218	0.22669999	0.57529998	0.57800001	0.57359999	0.53789997
0.22679999	0.22139999	0.2243	0.57639998	0.57840002	0.57620001	0.53359997
0.2291	0.22229999	0.22579999	0.57679999	0.5772	0.57389998	0.53890002
0.22840001	0.222	0.2229	0.57639998	0.57910001	0.57690001	0.53740001
0.2288	0.2247	0.22400001	0.57489997	0.57880002	0.57410002	0.53789997
0.2297	0.223	0.22239999	0.57679999	0.5776	0.57499999	0.53659999

0.2324	0.22310001	0.22480001	0.5751	0.57980001	0.57440001	0.53600001
0.2325	0.22220001	0.2243	0.57609999	0.57770002	0.57419997	0.54149997
0.23019999	0.2221	0.2253	0.57489997	0.57819998	0.574	0.53750002
0.2297	0.22229999	0.2277	0.574	0.57590002	0.5733	0.53659999
0.2306	0.22149999	0.2234	0.57539999	0.5776	0.5747	0.53439999
0.2324	0.21969999	0.2219	0.5747	0.57789999	0.57429999	0.537
0.23019999	0.2216	0.2207	0.574	0.5758	0.5711	0.53719997
0.22920001	0.22229999	0.2228	0.57359999	0.57609999	0.56849998	0.53839999
0.2295	0.222	0.2191	0.57520002	0.57840002	0.57560003	0.53649998
0.2288	0.2207	0.2181	0.57550001	0.57690001	0.57410002	0.53789997
0.228	0.219	0.22220001	0.57410002	0.57609999	0.5751	0.53609997
0.2297	0.2194	0.2198	0.57450002	0.57529998	0.57200003	0.53509998
0.2271	0.22	0.22050001	0.57239997	0.57550001	0.57569999	0.53469998
0.2245	0.2208	0.22130001	0.57440001	0.57669997	0.57520002	0.53799999
0.22589999	0.22130001	0.21799999	0.57300001	0.57319999	0.5715	0.537
			0.57209998	0.57410002	0.57179999	0.53890002
			0.57440001	0.57810003	0.5733	0.53530002
			0.57249999	0.57270002	0.57279998	0.53860003
			0.57260001	0.57709998	0.57370001	0.53960001
			0.57440001	0.57560003	0.57249999	0.53689998
			0.57230002	0.57349998	0.57090002	0.53509998
			0.57440001	0.5783	0.57599998	0.53750002
			0.57059997	0.57529998	0.57349998	0.53850001
			0.57260001	0.57340002	0.57190001	
			0.57319999	0.57389998	0.57139999	
			0.57309997	0.57550001	0.57499999	
			0.57120001	0.57319999	0.56889999	

Эdel AcetylCoA - Ethanol pH7

2			3			1
0.5147	0.62629998	0.65530002	0.6681	0.80629998	0.1617	0.1696
0.56199998	0.66799998	0.71219999	0.65350002	0.79089999	0.162	0.1684
0.57569999	0.68660003	0.7647	0.66750002	0.77569997	0.1602	0.1672
0.58170003	0.6796	0.71319997	0.6573	0.75739998	0.1593	0.1665
0.58850002	0.68879998	0.75690001	0.6864	0.72310001	0.16230001	0.16850001
0.58840001	0.66829997	0.8071	0.70529997	0.73360002	0.1621	0.16859999
0.59210002	0.68080002	0.8089	0.72049999	0.722	0.1618	0.1693
0.59100002	0.6717	0.77829999	0.74089998	0.71820003	0.163	0.1701
0.58649999	0.6692	0.82410002	0.73089999	0.70770001	0.16249999	0.16779999
0.58829999	0.67720002	0.79839998	0.74809998	0.7119	0.1646	0.16859999
0.57980001	0.66659999	0.81050003	0.75120002	0.71820003	0.1601	0.1645
0.57489997	0.67330003	0.8039	0.76590002	0.7105	0.16320001	0.16869999
0.57810003	0.68489999	0.75040001	0.76880002	0.70520002	0.1656	0.16769999
0.57969999	0.6904	0.79290003	0.75190002	0.722	0.1594	0.1655
0.57550001	0.68019998	0.7974	0.74309999	0.72689998	0.16140001	0.1675
0.57789999	0.6857	0.74070001	0.75279999	0.74860001	0.16429999	0.1689
0.58090001	0.68800002	0.81150001	0.76179999	0.73909998	0.1612	0.16769999
0.58359998	0.69489998	0.8136	0.764	0.7536	0.161	0.16670001
0.5855	0.69260001	0.80110002	0.75760001	0.76370001	0.1613	0.16500001
0.58469999	0.68739998	0.75190002	0.7608	0.77130002	0.163	0.167
0.5819	0.68800002	0.80849999	0.75550002	0.773	0.1652	0.1639
0.5891	0.69330001	0.8143	0.77579999	0.79390001	0.16	0.1691
0.58710003	0.6929	0.82660002	0.74610001	0.77890003	0.1611	0.16419999
0.58719999	0.6904	0.81940001	0.74580002	0.78259999	0.1635	0.1666
0.58859998	0.68720001	0.83469999	0.76620001	0.78130001	0.1646	0.1664
0.58660001	0.68440002	0.76609999	0.74760002	0.78909999	0.15989999	0.16689999
0.58660001	0.68550003	0.81900001	0.74529999	0.79009998	0.1619	0.1691
0.58389997	0.68769997	0.81379998	0.74980003	0.7881	0.1631	0.168
0.58660001	0.67930001	0.79369998	0.7428	0.78839999	0.15880001	0.1662
0.58990002	0.68519998	0.87120003	0.7471	0.7906	0.1611	0.1649
0.58929998	0.68529999	0.82709998	0.741	0.79210001	0.16140001	0.1657
0.5887	0.68279999	0.81470001	0.75050002	0.78399998	0.1631	0.1644
0.58660001	0.68699998	0.83670002	0.74470001	0.78829998	0.1628	0.1636
0.58569998	0.685	0.82819998	0.73360002	0.78869998	0.1609	0.1655
0.58780003	0.6778	0.66320002	0.63800001	0.64429998	0.1636	0.1674
0.50209999	0.54830003	0.61180001	0.60369998	0.62279999	0.26140001	0.2175
0.491	0.53539997	0.59859997	0.5923	0.61900002	0.2358	0.24420001
0.48140001	0.5309	0.5988	0.59310001	0.61580002	0.2669	0.27250001
0.4885	0.54100001	0.59689999	0.59820002	0.6178	0.2933	0.29049999
0.49759999	0.5442	0.5966	0.59469998	0.61549997	0.32570001	0.324
0.50739998	0.55220002	0.59670001	0.58899999	0.61309999	0.3592	0.35159999
0.51169997	0.55430001	0.60039997	0.59130001	0.61830002	0.39300001	0.38569999
0.51249999	0.55629998	0.59109998	0.5905	0.60939997	0.42550001	0.4152
0.5176	0.55470002	0.5934	0.58560002	0.61360002	0.45989999	0.44729999

0.52609998	0.5654	0.59219998	0.58960003	0.62050003	0.49919999	0.4797
0.52600002	0.56730002	0.60030001	0.59109998	0.61430001	0.53299999	0.51120001
0.52990001	0.57300001	0.60110003	0.59210002	0.61669999	0.56660002	0.54189998
0.53280002	0.57849997	0.6013	0.59649998	0.61369997	0.60000002	0.57380003
0.53469998	0.5855	0.6006	0.59189999	0.6153	0.6286	0.60180002
0.53329998	0.59299999	0.6027	0.59859997	0.61390001	0.65570003	0.62870002
0.53490001	0.59869999	0.60799998	0.60189998	0.61400002	0.68229997	0.65679997
0.53579998	0.5984	0.6045	0.60439998	0.6128	0.70819998	0.68059999
0.5327	0.59829998	0.60729998	0.60399997	0.6189	0.72680002	0.70090002
0.53509998	0.60470003	0.6171	0.5995	0.61589998	0.74720001	0.7227
0.53430003	0.59969997	0.61610001	0.61210001	0.61760002	0.76490003	0.73970002
0.53310001	0.6006	0.61610001	0.61330003	0.61690003	0.78130001	0.75959998
0.53500003	0.59780002	0.61989999	0.61390001	0.63249999	0.80000001	0.7755
0.53189999	0.59500003	0.62330002	0.61299998	0.62309998	0.81559998	0.79030001
0.53189999	0.59859997	0.62529999	0.62080002	0.62669998	0.82770002	0.80369997
0.5352	0.59869999	0.6232	0.62669998	0.63099998	0.84579998	0.82139999
0.53350002	0.5988	0.62940001	0.62599999	0.6365	0.86110002	0.83670002
0.53119999	0.59810001	0.63529998	0.62709999	0.63739997	0.87459999	0.84759998
0.53399998	0.59859997	0.62510002	0.62379998	0.63169998	0.88770002	0.86379999
0.53280002	0.59969997	0.63419998	0.62959999	0.63590002	0.9012	0.87410003
0.53219998	0.59750003	0.63499999	0.63010001	0.63749999	0.9138	0.8876
0.53280002	0.59890002	0.63819999	0.62830001	0.6347	0.92580003	0.89780003
0.5352	0.59829998	0.63779998	0.63059998	0.63870001	0.9418	0.912
0.5316	0.5959	0.63669997	0.63739997	0.63800001	0.95169997	0.92750001
0.53079998	0.59500003	0.63810003	0.63459998	0.63510001	0.96740001	0.94050002
0.5302	0.59890002	0.64230001	0.63749999	0.64270002	0.97680002	0.95020002
0.53250003	0.59460002	0.63880002	0.63270003	0.64270002	0.98689997	0.96170002
0.52929997	0.59549999	0.63880002	0.6318	0.63779998	1.00209999	0.97399998
0.53500003	0.59850001	0.6498	0.63749999	0.64520001	1.01320004	0.98379999
0.52789998	0.597	0.63849998	0.63679999	0.64249998	1.02530003	0.99370003
0.52969998	0.59850001	0.63840002	0.63870001	0.64179999	1.03789997	1.00870001
0.53420001	0.597	0.64420003	0.64219999	0.63840002	1.04799998	1.01520002
0.53170002	0.5941	0.63789999	0.63859999	0.639	1.05780005	1.02880001
0.53130001	0.5952	0.6419	0.63800001	0.64279997	1.07070005	1.04079998
0.5316	0.59750003	0.6426	0.6372	0.64219999	1.08539999	1.05280006
0.5298	0.5959	0.64450002	0.6365	0.64340001	1.0927	1.06200004
0.52829999	0.59329998	0.6372	0.63620001	0.63990003	1.10339999	1.07210004
0.52950001	0.5984	0.63410002	0.63459998	0.64139998	1.11170006	1.07739997
0.52560002	0.59630001	0.63700002	0.63410002	0.63940001	1.12440002	1.0941
0.5334	0.59289998	0.64249998	0.63569999	0.64090002	1.13160002	1.09959996
0.52780002	0.59310001	0.63709998	0.63520002	0.64520001	1.14250004	1.11290002
0.52710003	0.59039998	0.64090002	0.63270003	0.63789999	1.15579998	1.12419999
0.5273	0.59560001	0.64630002	0.63499999	0.6444	1.16400003	1.13390005
0.52609998	0.59579998	0.63550001	0.63529998	0.64179999	1.17289996	1.14330006
0.52780002	0.59350002	0.63789999	0.63810003	0.63630003	1.18400002	1.15310001
0.5262	0.59350002	0.63749999	0.63749999	0.6365	1.19239998	1.1609

0.53030002	0.5927	0.64780003	0.63450003	0.64219999	1.20089996	1.16999996
0.52829999	0.59119999	0.63739997	0.63499999	0.64420003	1.21029997	1.18149996
0.52640003	0.59079999	0.6419	0.63410002	0.63990003	1.22000003	1.18509996
0.5266	0.59369999	0.6437	0.63429999	0.64349997	1.22969997	1.19910002
0.52520001	0.5923	0.63429999	0.63459998	0.63690001	1.24070001	1.2076
0.52859998	0.59259999	0.63590002	0.63410002	0.63940001	1.24269998	1.21239996
0.52530003	0.58850002	0.6426	0.63620001	0.63849998	1.25349998	1.22370005
0.52740002	0.5898	0.63679999	0.63279998	0.6451	1.26209998	1.23220003
0.52490002	0.59280002	0.63709998	0.63209999	0.6365	1.27040005	1.24249995
0.5255	0.59259999	0.63880002	0.63	0.63709998	1.27839994	1.24740005
0.5273	0.58719999	0.64120001	0.63279998	0.64120001	1.28499997	1.25489998
0.52789998	0.58539999	0.64810002	0.63459998	0.63849998	1.29770005	1.26689994
0.5248	0.59009999	0.6462	0.63429999	0.63919997	1.30410004	1.2744
0.52670002	0.58719999	0.63849998	0.63169998	0.63840002	1.3089	1.28100002
0.52630001	0.588	0.6376	0.62970001	0.63849998	1.31799996	1.28869998
0.52829999	0.5934	0.63669997	0.63330001	0.63779998	1.32179999	1.29419994
0.52520001	0.58859998	0.63489997	0.63309997	0.63700002	1.33099997	1.30149996
0.52450001	0.59130001	0.6354	0.62870002	0.6329	1.33889997	1.30659997
0.52490002	0.59039998	0.63510001	0.6336	0.63789999	1.34300005	1.31529999
0.52429998	0.59069997	0.63770002	0.6318	0.63679999	1.34979999	1.32449996
0.52670002	0.59189999	0.64429998	0.63270003	0.63770002	1.35689998	1.32959998
0.52649999	0.58649999	0.63739997	0.63349998	0.63990003	1.36570001	1.33609998
0.52450001	0.58679998	0.63779998	0.63260001	0.6358	1.36880004	1.34140003
0.52469999	0.58569998	0.63789999	0.62940001	0.63459998	1.37699997	1.34969997
0.52740002	0.58880001	0.6426	0.63160002	0.63309997	1.38489997	1.35440004
0.52520001	0.5898	0.63599998	0.63200003	0.6383	1.38590002	1.35609996
0.52139997	0.58969998	0.63260001	0.62800002	0.63410002	1.39479995	1.3678
0.52240002	0.58749998	0.64099997	0.63279998	0.63669997	1.40009999	1.3721
0.52749997	0.58600003	0.64230001	0.6376	0.63440001	1.41059995	1.38259995
0.523	0.58410001	0.63489997	0.62870002	0.63330001	1.41340005	1.38709998
0.52240002	0.58600003	0.63190001	0.63090003	0.63129997	1.4181	1.3944
0.52579999	0.58530003	0.63910002	0.63160002	0.62809998	1.41980004	1.39390004
0.52329999	0.58829999	0.6376	0.63080001	0.63260001	1.42420006	1.39900005
0.52319998	0.58759999	0.63120002	0.63160002	0.63499999	1.43610001	1.40849996
0.5219	0.583	0.63910002	0.6354	0.63669997	1.43640006	1.41170001
0.52179998	0.58160001	0.63239998	0.62709999	0.63529998	1.44379997	1.41939998
0.5212	0.58560002	0.65140003	0.62959999	0.63370001	1.44920003	1.42410004
0.5194	0.5826	0.64590001	0.62760001	0.63529998	1.45070004	1.42719996
0.52170002	0.58389997	0.6322	0.62919998	0.63489997	1.45669997	1.43410003
0.52179998	0.5848	0.63209999	0.62949997	0.63679999	1.46169996	1.4382
0.52350003	0.58759999	0.63319999	0.62980002	0.63880002	1.46829998	1.44480002
0.52090001	0.58270001	0.63319999	0.62739998	0.6365	1.47119999	1.44630003
0.51859999	0.58469999	0.63340002	0.6225	0.63370001	1.47609997	1.45449996
0.52359998	0.58139998	0.62940001	0.62330002	0.62959999	1.4799	1.45790005
0.5212	0.5851	0.6408	0.62949997	0.62900001	1.4806	1.45869994
0.52109998	0.58649999	0.63209999	0.625	0.62370002	1.48609996	1.46169996

0.52020001	0.58649999	0.63330001	0.62779999	0.634	1.48989999	1.46819997
0.52170002	0.5812	0.63669997	0.62379998	0.63370001	1.49810004	1.47420001
0.52160001	0.58670002	0.64429998	0.63489997	0.63370001	1.49940002	1.48070002
0.52170002	0.58410001	0.63330001	0.62690002	0.63499999	1.50399995	1.48119998
0.5219	0.58149999	0.63419998	0.62379998	0.62989998	1.5043	1.4835
0.51810002	0.58109999	0.63849998	0.62769997	0.63270003	1.50730002	1.48629999
0.52289999	0.58359998	0.63139999	0.62269998	0.63410002	1.51629996	1.4921
0.51910001	0.58410001	0.6383	0.6293	0.63330001	1.5165	1.49559999
0.5219	0.58069998	0.63090003	0.62699997	0.63080001	1.52059996	1.50300002
0.5205	0.57819998	0.63279998	0.6214	0.6275	1.52250004	1.50109994
0.52170002	0.58020002	0.62779999	0.62080002	0.62279999	1.52209997	1.50139999
0.52039999	0.58279997	0.63029999	0.62440002	0.62669998	1.52989995	1.50870001
0.52060002	0.58230001	0.62540001	0.62330002	0.62550002	1.53799999	1.51849997
0.52179998	0.58469999	0.63080001	0.62279999	0.62809998	1.53659999	1.51670003
0.51849997	0.58270001	0.63050002	0.62279999	0.62949997	1.53719997	1.51769996
0.51840001	0.5765	0.62800002	0.6207	0.62870002	1.54369998	1.5266
0.51800001	0.57800001	0.62900001	0.6207	0.62949997	1.54750001	1.52789998
0.52380002	0.58090001	0.6318	0.6322	0.62629998	1.54560006	1.52699995
0.52249998	0.58050001	0.63050002	0.62419999	0.6268	1.54890001	1.5302
0.51889998	0.58139998	0.63569999	0.62809998	0.6239	1.55359995	1.53540003
0.51990002	0.57969999	0.63950002	0.6232	0.62599999	1.55719995	1.53789997
0.52109998	0.57990003	0.63150001	0.62540001	0.62970001	1.55760002	1.53840005
0.51810002	0.57800001	0.62510002	0.62489998	0.63090003	1.5582	1.53859997
0.52100003	0.57749999	0.62519997	0.6225	0.6257	1.56509995	1.54709995
0.51849997	0.57920003	0.62919998	0.62099999	0.62220001	1.56620002	1.54840004
0.5219	0.58499998	0.6221	0.61830002	0.62449998	1.56700003	1.54920006
0.52020001	0.5848	0.62199998	0.61930001	0.62220001	1.56959999	1.5503
0.5183	0.57779998	0.62459999	0.6207	0.62639999	1.57620001	1.55999994
0.52079999	0.57770002	0.62849998	0.62360001	0.6318	1.57190001	1.5517
0.51749998	0.58060002	0.62239999	0.62169999	0.62730002	1.57770002	1.56130004
0.52090001	0.57740003	0.6239	0.62290001	0.63	1.58099997	1.56649995
0.52289999	0.57730001	0.62709999	0.61930001	0.63279998	1.58239996	1.5654
0.5183	0.58230001	0.6311	0.62239999	0.62669998	1.58150005	1.56780005
0.52020001	0.57669997	0.6365	0.62190002	0.63340002	1.58870006	1.56700003
0.51889998	0.57389998	0.62459999	0.62220001	0.62830001	1.58780003	1.57200003
0.51639998	0.57660002	0.63620001	0.62019998	0.62349999	1.59099996	1.57519996
0.51800001	0.58090001	0.62519997	0.62029999	0.63139999	1.58930004	1.57299995
0.51819998	0.5765	0.61930001	0.61769998	0.63309997	1.59430003	1.57959998
0.51789999	0.57520002	0.62620002	0.62010002	0.63389999	1.59650004	1.58060002
0.51980001	0.57749999	0.62760001	0.6207	0.62989998	1.5977	1.58290005
0.51590002	0.57690001	0.62199998	0.62040001	0.62279999	1.60039997	1.58270001
0.51520002	0.57840002	0.62449998	0.61849999	0.61629999	1.59599996	1.58060002
0.51709998	0.57450002	0.62559998	0.62330002	0.61860001	1.60399997	1.58829999
0.51849997	0.57489997	0.61909997	0.61900002	0.62419999	1.60319996	1.58780003
0.51889998	0.57620001	0.62230003	0.61790001	0.61949998	1.60520005	1.5891
0.51310003	0.57730001	0.63120002	0.62559998	0.61909997	1.6085	1.59060001

0.5169	0.5765	0.61970001	0.62059999	0.61919999	1.60759997	1.59150004
0.51529998	0.57429999	0.6214	0.61940002	0.62639999	1.60739994	1.59379995
0.51630002	0.57609999	0.62510002	0.61519998	0.61580002	1.61049998	1.59539998
0.51529998	0.57499999	0.62260002	0.61790001	0.62470001	1.61049998	1.59290004
0.51499999	0.5758	0.62279999	0.61989999	0.62809998	1.61150002	1.59549999
0.51130003	0.57709998	0.62769997	0.61979997	0.62470001	1.61619997	1.60039997
0.51920003	0.57700002	0.62449998	0.6275	0.616	1.61450005	1.60020006
0.514	0.57130003	0.62120003	0.62300003	0.62040001	1.62199998	1.60539997
0.51419997	0.5747	0.62720001	0.61659998	0.61580002	1.61539996	1.60350001
0.51529998	0.57370001	0.61839998	0.61750001	0.61900002	1.61940002	1.60619998
0.51270002	0.57300001	0.62629998	0.616	0.6171	1.62460005	1.60640001
0.514	0.57249999	0.61739999	0.6146	0.61739999		
0.5169	0.57529998	0.62290001	0.61540002	0.62029999		
0.5133	0.57050002	0.6142	0.61180001	0.61250001		
0.51429999	0.57239997	0.62059999	0.6261	0.6232		
0.51620001	0.57480001	0.625	0.61919999	0.61729997		
0.51340002	0.57309997	0.62029999	0.61409998	0.61449999		
0.51300001	0.57160002	0.62120003	0.61360002	0.61430001		
0.51050001	0.57190001	0.61860001	0.61260003	0.6128		
0.51289999	0.57249999	0.6178	0.61250001	0.61720002		
0.51450002	0.5686	0.63260001	0.61629999	0.61500001		
0.51550001	0.5722	0.61870003	0.6142	0.61659998		
0.51289999	0.57139999	0.616	0.6135	0.6146		
0.51520002	0.57459998	0.63819999	0.6189	0.6178		
0.51490003	0.5747	0.61809999	0.61739999	0.61290002		
0.51700002	0.57239997	0.61379999	0.61849999	0.61379999		
0.51380002	0.5697	0.61570001	0.61680001	0.61549997		
0.51569998	0.57160002	0.6164	0.61140001	0.6124		
0.51349998	0.56950003	0.61870003	0.61189997	0.61619997		
0.5151	0.57279998	0.62279999	0.61750001	0.61790001		
0.51419997	0.5711	0.6142	0.61250001	0.61619997		
0.50929999	0.56870002	0.6182	0.61680001	0.61540002		
0.5122	0.56940001	0.61839998	0.61409998	0.61669999		
0.51169997	0.56779999	0.62279999	0.6153	0.61580002		
0.51099998	0.57080001	0.61519998	0.61180001	0.61320001		
0.51209998	0.57130003	0.62129998	0.61769998	0.61290002		
0.51310003	0.57300001	0.62519997	0.61210001	0.60860002		
0.51300001	0.5675	0.61299998	0.61580002	0.60970002		
0.51429999	0.56989998	0.6221	0.61309999	0.61119998		
0.51370001	0.56660002	0.61919999	0.61049998	0.61220002		
0.51020002	0.57059997	0.62099999	0.61330003	0.61210001		
0.51190001	0.57029998	0.61619997	0.61000001	0.61129999		
0.51239997	0.56980002	0.61610001	0.61269999	0.61089998		
0.5108	0.56889999	0.6124	0.61390001	0.61019999		
0.51230001	0.56989998	0.61250001	0.60900003	0.61140001		
0.51069999	0.56699997	0.62269998	0.60979998	0.6063		

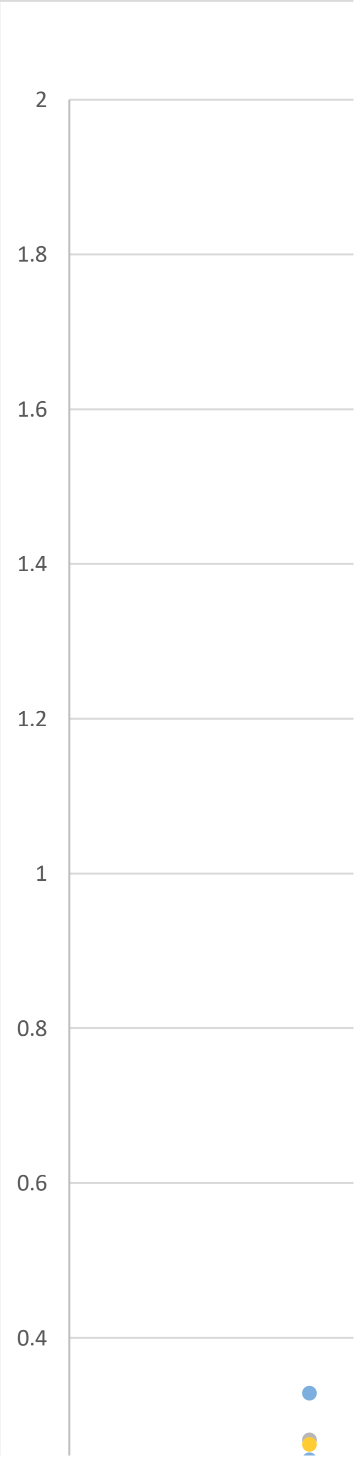

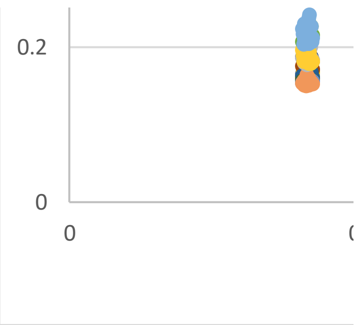

0.51109999	0.56849998	0.61510003	0.60650003	0.61269999	[illegible]	[illegible]
0.51099998	0.56889999	0.61760002	0.60930002	0.6142	[illegible]	[illegible]
0.509	0.56720001	0.61559999	0.61070001	0.61680001	[illegible]	[illegible]
0.5108	0.56699997	0.61650002	0.60949999	0.6178	[illegible]	[illegible]
0.50669998	0.56489998	0.61669999	0.61049998	0.61430001	[illegible]	[illegible]
0.51099998	0.56580001	0.6103	0.60839999	0.6128	[illegible]	[illegible]
0.50840002	0.5675	0.61699998	0.61080003	0.61809999	[illegible]	[illegible]
0.51120001	0.56980002	0.62349999	0.61769998	0.62339997	[illegible]	[illegible]
0.51099998	0.56889999	0.61150002	0.60869998	0.62010002	1.64999998	1.6401
0.51099998	0.56730002	0.61430001	0.60650003	0.62510002	1.65409994	1.64279997
0.50950003	0.56419998	0.61669999	0.60979998	0.6153	1.65250003	1.64209998
0.5097	0.56379998	0.61629999	0.61369997	0.61669999	1.65649998	1.64139998
0.51010001	0.56770003	0.61930001	0.60680002	0.60750002	1.65369999	1.64250004
0.50940001	0.565	0.61070001	0.60710001	0.6074	1.65540004	1.64520001
0.50950003	0.56459999	0.62279999	0.61080003	0.61189997	1.65629995	1.64600003
0.50999999	0.56550002	0.60960001	0.61119998	0.61180001	1.65999997	1.64719999
0.50980002	0.5675	0.61159998	0.60820001	0.61900002	1.65629995	1.64479995
0.51130003	0.56580001	0.6124	0.60600001	0.61839998	1.65629995	1.64849997
0.51319999	0.56599998	0.6153	0.611	0.62040001	1.66059995	1.64670002
0.51069999	0.56550002	0.60879999	0.60820001	0.6171	1.65540004	1.64330006
0.51099998	0.56339997	0.61339998	0.60970002	0.61870003	1.65999997	1.64590001
0.50999999	0.56760001	0.60869998	0.60680002	0.61809999	1.65869999	1.64269996
0.5108	0.5618	0.60790002	0.61059999	0.6142	1.65849996	1.64789999
0.50730002	0.56279999	0.61260003	0.60549998	0.61619997	1.65890002	1.64660001
0.51010001	0.56650001	0.61510003	0.61299998	0.62150002	1.65900004	1.6487
0.50880003	0.5661	0.6189	0.6067	0.61400002	1.65970004	1.64810002
0.51179999	0.56819999	0.60979998	0.60640001	0.61390001	1.65929997	1.64680004
0.51050001	0.56279999	0.60680002	0.60689998	0.61290002	1.65859997	1.64670002
0.50779998	0.56019998	0.61570001	0.60900003	0.61390001	1.66079998	1.65240002
0.50629997	0.5632	0.60900003	0.60699999	0.61400002	1.66030002	1.65219998
0.50989997	0.5632	0.62019998	0.60579997	0.61059999	1.66429996	1.65149999
0.50999999	0.56190002	0.61229998	0.60500002	0.61089998	1.65509999	1.64600003
0.5072	0.56010002	0.61479998	0.60710001	0.61009997	1.65939999	1.64820004
0.50690001	0.56160003	0.6074	0.60979998	0.61140001	1.66199994	1.65219998
0.50849998	0.5625	0.61129999	0.61009997	0.6124	1.66129994	1.65359998
0.5079	0.5636	0.60979998	0.60720003	0.61220002	1.66569996	1.65709996
0.51120001	0.56449997	0.61669999	0.60280001	0.60720003	1.66100001	1.64950001
0.50749999	0.56419998	0.62220001	0.61049998	0.6056	1.65779996	1.64779997
0.50440001	0.56150001	0.61320001	0.60360003	0.6103	1.66129994	1.64989996
0.50620002	0.56230003	0.61229998	0.59930003	0.60479999	1.66550004	1.65439999
0.50919998	0.5636	0.60470003	0.60650003	0.60500002	1.66260004	1.65069997
0.50599998	0.56260002	0.60790002	0.60589999	0.6092	1.66030002	1.6494
0.5072	0.55879998	0.61720002	0.60390002	0.6099	1.66470003	1.6523
0.5108	0.56160003	0.60780001	0.60360003	0.60470003	1.66129994	1.65079999
0.50800002	0.56389999	0.60570002	0.60210001	0.60750002	1.66509998	1.65559995
0.50590003	0.56300002	0.61040002	0.6024	0.60839999	1.66199994	1.64950001

0.50690001	0.56129998	0.60519999	0.60509998	0.61369997	1.66129994	1.65380001
0.50880003	0.56300002	0.60409999	0.60280001	0.61119998	1.66369998	1.65409994
0.50550002	0.56269997	0.60589999	0.6056	0.61940002	1.66579998	1.65339994
0.50760001	0.56230003	0.6024	0.60070002	0.60839999	1.66250002	1.65149999
0.50959998	0.5632	0.6049	0.602	0.61519998	1.66170001	1.65190005
0.50819999	0.55989999	0.60320002	0.6006	0.61180001	1.66610003	1.65540004
0.50529999	0.56	0.61500001	0.6081	0.6092	1.65840006	1.64740002
0.50700003	0.55690002	0.61489999	0.59920001	0.60470003	1.66509998	1.65359998
0.50550002	0.55940002	0.60610002	0.60180002	0.60680002	1.66180003	1.65540004
0.50830001	0.55989999	0.6074	0.59990001	0.60780001	1.66530001	1.653
0.50730002	0.56269997	0.6074	0.60610002	0.60659999	1.6595	1.64989996
0.50809997	0.56169999	0.61210001	0.60479999	0.60689998	1.66439998	1.65069997
0.50779998	0.56019998	0.61799997	0.60579997	0.60409999	1.66050005	1.64929998
0.50590003	0.55860001	0.6045	0.6024	0.60610002	1.66260004	1.65530002
0.50819999	0.5596	0.60869998	0.6074	0.60540003	1.66050005	1.65050006
0.50650001	0.56080002	0.60710001	0.60149997	0.60519999	1.66050005	1.64929998
0.50489998	0.5614	0.60570002	0.6038	0.60519999	1.66429996	1.65339994
0.50660002	0.56019998	0.61199999	0.60070002	0.6031	1.66489995	1.6552
0.50819999	0.55860001	0.60610002	0.59990001	0.60329998	1.66229999	1.65170002
0.50550002	0.56089997	0.6063	0.59630001	0.60470003	1.66460001	1.65480006
0.50569999	0.55970001	0.6056	0.59930003	0.60350001	1.66229999	1.65369999
0.50150001	0.55290002	0.6031	0.6045	0.60170001	1.66419995	1.64929998
0.50739998	0.5589	0.61019999	0.60100001	0.6038	1.66159999	1.65289998
0.50739998	0.5571	0.60339999	0.5977	0.60500002	1.66110003	1.653
0.50449997	0.5553	0.6038	0.59619999	0.60600001	1.66509998	1.65149999
0.50449997	0.55860001	0.60320002	0.59810001	0.60360003	1.66219997	1.6516
0.50669998	0.55870003	0.59869999	0.59780002	0.59850001	1.66059995	1.65149999
0.50220001	0.55879998	0.60390002	0.59969997	0.6056	1.66789997	1.65330005
0.50349998	0.55489999	0.60519999	0.60009998	0.6049	1.66320002	1.64979994
0.50389999	0.55620003	0.6067	0.59829998	0.61140001	1.66009998	1.65139997
0.50770003	0.55830002	0.60790002	0.59509999	0.6056	1.65719998	1.64839995
0.50300002	0.55809999	0.611	0.59820002	0.60479999	1.6631	1.65050006
0.50260001	0.55690002	0.60409999	0.59750003	0.60420001	1.66390002	1.65199995
0.50370002	0.55610001	0.59960002	0.60149997	0.6045	1.66480005	1.65320003
0.5025	0.55650002	0.60439998	0.60009998	0.60409999	1.66180003	1.65139997
0.50400001	0.55589998	0.60079998	0.5977	0.60079998	1.65880001	1.65079999
0.5043	0.5553	0.60399997	0.59869999	0.6049	1.66219997	1.653
0.50739998	0.55930001	0.60070002	0.5948	0.60329998	1.66079998	1.64810002
0.5043	0.55599999	0.6045	0.6002	0.60399997	1.65900004	1.65279996
0.50370002	0.55680001	0.59930003	0.59789997	0.611	1.6638	1.64989996
0.50559998	0.55500001	0.60110003	0.59820002	0.6031	1.6595	1.65059996
0.50269997	0.55400002	0.60180002	0.59649998	0.59979999	1.66159999	1.64900005
0.5036	0.55400002	0.60280001	0.60049999	0.611	1.66390002	1.65339994
0.50449997	0.55739999	0.60039997	0.59930003	0.60350001	1.66419995	1.65250003
0.50400001	0.55610001	0.59909999	0.59530002	0.60030001	1.66209996	1.65209997
0.50580001	0.55589998	0.60369998	0.60299999	0.59890002	1.6631	1.65100002

0.50620002	0.55870003	0.59920001	0.597	0.60360003	1.66040003	1.64740002
0.50169998	0.55540001	0.6013	0.59820002	0.60159999	1.66190004	1.65199995
0.50260001	0.55549997	0.60420001	0.59890002	0.60320002	1.6595	1.6487
0.5025	0.5521	0.60320002	0.59820002	0.60210001	1.65970004	1.65330005
0.50150001	0.5528	0.6063	0.59619999	0.60180002	1.66059995	1.65139997
0.50349998	0.55400002	0.60680002	0.59820002	0.59890002	1.65910006	1.64619994
0.50340003	0.55489999	0.60339999	0.59439999	0.59850001	1.65869999	1.65009999
0.5007	0.55309999	0.6013	0.597	0.6002	1.65980005	1.64950001
0.50370002	0.55549997	0.61080003	0.5977	0.60030001	1.66120005	1.64769995
0.50220001	0.55409998	0.6027	0.60070002	0.60329998	1.6552	1.64670002
0.50199997	0.55229998	0.60009998	0.59850001	0.60360003	1.6602	1.64789999
0.5018	0.5528	0.60320002	0.5977	0.60729998	1.66180003	1.64680004
0.50129998	0.55470002	0.60259998	0.59689999	0.60110003	1.66009998	1.65110004
0.50169998	0.55250001	0.59780002	0.59259999	0.60070002	1.65760005	1.64769995
0.5018	0.55479997	0.59670001	0.59240001	0.6002	1.65750003	1.65050006
0.5007	0.55250001	0.59719998	0.59350002	0.59899998	1.65690005	1.6487
0.5036	0.55330002	0.60110003	0.59820002	0.6027	1.65680003	1.64680004
0.50220001	0.55330002	0.60530001	0.59640002	0.60530001	1.65999997	1.6473
0.50209999	0.55229998	0.59670001	0.58960003	0.59890002	1.65779996	1.64590001
0.50239998	0.55379999	0.60180002	0.59600002	0.5977	1.66159999	1.64999998
0.50150001	0.55369997	0.60820001	0.60049999	0.6002	1.65929997	1.64670002
0.50230002	0.55489999	0.60119998	0.59350002	0.60049999	1.65530002	1.64610004
0.50129998	0.5521	0.60030001	0.59530002	0.5984	1.65910006	1.64639997
0.50050002	0.55180001	0.6002	0.59680003	0.6006	1.65779996	1.64810002
0.49959999	0.55330002	0.60089999	0.59140003	0.60360003	1.65489995	1.64709997
0.50150001	0.55190003	0.5977	0.59039998	0.60089999	1.65820003	1.6473
0.49939999	0.55290002	0.60219997	0.59570003	0.59799999	1.65799999	1.6444
0.4993	0.54930001	0.59560001	0.59119999	0.59789997	1.65890002	1.64610004
0.5025	0.55330002	0.59509999	0.59130001	0.6002	1.65890002	1.65050006
0.4989	0.55140001	0.60100001	0.59240001	0.60720003	1.65320003	1.64339995
0.50080001	0.55129999	0.5941	0.5941	0.59729999	1.65320003	1.64269996
0.5007	0.55129999	0.60000002	0.58749998	0.59350002	1.65409994	1.64489996
0.50129998	0.5521	0.59890002	0.59649998	0.60250002	1.6566	1.64639997
0.498	0.5485	0.59729999	0.59570003	0.59799999	1.6595	1.64690006
0.50019997	0.55119997	0.59640002	0.59130001	0.60049999	1.65450001	1.64489996
0.49990001	0.55110002	0.59640002	0.59189999	0.60110003	1.65409994	1.64339995
0.50040001	0.55150002	0.59320003	0.58579999	0.59240001	1.65219998	1.6408
0.49919999	0.54949999	0.6038	0.59289998	0.59869999	1.65040004	1.63919997
0.4993	0.551	0.60110003	0.58999997	0.59680003	1.65740001	1.64289999
0.49790001	0.54860002	0.59680003	0.59450001	0.60049999	1.65439999	1.64690006
0.50040001	0.54960001	0.5984	0.59369999	0.59719998	1.65610003	1.64839995
0.50029999	0.55159998	0.59530002	0.58789998	0.59509999	1.65489995	1.64559996
0.49970001	0.54909998	0.59429997	0.59069997	0.60030001	1.65069997	1.64160001
0.4962	0.54799998	0.60780001	0.5948	0.59640002	1.65269995	1.63759995
0.49900001	0.54869998	0.59490001	0.58649999	0.59369999	1.65100002	1.63870001
0.50019997	0.55000001	0.59649998	0.58850002	0.59490001	1.65450001	1.64250004

0.50080001	0.54909998	0.59469998	0.59030002	0.59539998	1.65310001	1.64040005
0.50150001	0.55129999	0.59549999	0.58859998	0.5966	1.6516	1.64300001
0.4962	0.54879999	0.59649998	0.59909999	0.59460002	1.65250003	1.64170003
0.4971	0.54659998	0.597	0.59069997	0.5948	1.65429997	1.64450002
0.49700001	0.54909998	0.59439999	0.59140003	0.5995	1.65250003	1.64059997
0.50050002	0.551	0.60890001	0.59259999	0.59799999	1.65390003	1.64479995
0.5007	0.54809999	0.59140003	0.59060001	0.59670001	1.64810002	1.63699996
0.4966	0.5478	0.5923	0.58749998	0.59500003	1.64649999	1.63909996
0.49559999	0.54930001	0.59369999	0.58670002	0.59619999	1.6559	1.64090002
0.49790001	0.54900002	0.597	0.58359998	0.59469998	1.65020001	1.63979995
0.49669999	0.54860002	0.59259999	0.5887	0.59369999	1.64900005	1.6408
0.498	0.54790002	0.59130001	0.58490002	0.59460002	1.64890003	1.64339995
0.49529999	0.54699999	0.59109998	0.59799999	0.59149998	1.64690006	1.63750005
0.4973	0.5478	0.58990002	0.588	0.59810001	1.64859998	1.63619995
0.4973	0.55010003	0.59609997	0.5844	0.59979999	1.64890003	1.64059997
0.4973	0.54809999	0.5959	0.60119998	0.59859997	1.64999998	1.63940001
0.50010002	0.54909998	0.58969998	0.58639997	0.59289998	1.64750004	1.63730001
0.49759999	0.54650003	0.59179997	0.58740002	0.5927	1.64880002	1.63769996
0.498	0.5474	0.58710003	0.58569998	0.59439999	1.64750004	1.64069998
0.4957	0.54729998	0.59899998	0.58850002	0.59609997	1.64690006	1.63429999
0.49739999	0.54659998	0.59670001	0.59439999	0.59189999	1.64320004	1.63419998
0.49869999	0.5485	0.58969998	0.58399999	0.59329998	1.64600003	1.63629997
0.4964	0.54699999	0.59140003	0.58630002	0.59429997	1.64909995	1.63779998
0.49700001	0.54619998	0.60030001	0.5909	0.59280002	1.64349997	1.63750005
0.4971	0.5467	0.58960003	0.58560002	0.59560001	1.64760005	1.63419998
0.4946	0.54750001	0.59390002	0.58880001	0.60030001	1.64590001	1.63740003
0.4982	0.54549998	0.58859998	0.59179997	0.59759998	1.64750004	1.63660002
0.49270001	0.54360002	0.59289998	0.58759999	0.59450001	1.64540005	1.63440001
0.49309999	0.54519999	0.59600002	0.59320003	0.59810001	1.64569998	1.63559997
0.4955	0.54570001	0.5898	0.5855	0.5959	1.64709997	1.63590002
0.49540001	0.54430002	0.5887	0.58399999	0.59390002	1.6451	1.63380003
0.49520001	0.54629999	0.59100002	0.58209997	0.59719998	1.6451	1.6365
0.4964	0.54360002	0.59460002	0.5869	0.59799999	1.64170003	1.63409996
0.4941	0.54509997	0.5898	0.58939999	0.59939998	1.64569998	1.63460004
0.49340001	0.54409999	0.59249997	0.58649999	0.59380001	1.64230001	1.63310003
0.4955	0.54339999	0.59930003	0.58710003	0.59249997	1.64590001	1.63549995
0.4955	0.54519999	0.58829999	0.57950002	0.59509999	1.64619994	1.6372
0.4964	0.54720002	0.59189999	0.59119999	0.58950001	1.64119995	1.62759995
0.49779999	0.54710001	0.59009999	0.58130002	0.59680003	1.64330006	1.63450003
0.49329999	0.54360002	0.58810002	0.58829999	0.60519999	1.64520001	1.63160002
0.49470001	0.5442	0.59490001	0.59189999	0.59200001	1.64330006	1.63349998
0.4941	0.54570001	0.60219997	0.583	0.58929998	1.64310002	1.63450003
0.4957	0.54470003	0.5873	0.58359998	0.5948	1.64250004	1.63240004
0.49239999	0.5424	0.58929998	0.58340001	0.59189999	1.64090002	1.63100004
0.4946	0.5424	0.58960003	0.58420002	0.5984	1.64230001	1.63399994
0.49579999	0.54500002	0.59600002	0.58840001	0.59450001	1.64059997	1.62909997

0.4937	0.54140002	0.58679998	0.58380002	0.59240001	1.64279997	1.63240004
0.49270001	0.54159999	0.5869	0.58560002	0.59670001	1.64680004	1.63429999
0.49430001	0.54259998	0.58660001	0.58050001	0.59439999	1.64069998	1.6286
0.49470001	0.54149997	0.588	0.58840001	0.59210002	1.63849998	1.62750006
0.4921	0.54269999	0.60000002	0.58890003	0.59469998	1.64030004	1.62759995
0.49340001	0.54350001	0.58789998	0.58310002	0.59240001	1.64100003	1.63189995
0.49270001	0.5431	0.5891	0.58099997	0.5927	1.64189994	1.63090003
0.4941	0.5424	0.59149998	0.58679998	0.59490001	1.6365	1.62380004
0.4928	0.54119998	0.58749998	0.583	0.58920002	1.63660002	1.62960005
0.49380001	0.54360002	0.58960003	0.58410001	0.58810002	1.63970006	1.62899995
0.49309999	0.54259998	0.5905	0.58829999	0.58990002	1.63479996	1.6279
0.49360001	0.54119998	0.5923	0.5869	0.59759998	1.63680005	1.6243
0.48969999	0.54049999	0.58700001	0.58240002	0.5891	1.63610005	1.6257
0.49259999	0.54329997	0.58819997	0.5862	0.58560002	1.63440001	1.62720001
0.49470001	0.54530001	0.58410001	0.5801	0.59289998	1.63960004	1.62989998
0.4946	0.54229999				1.63020003	1.62170005
0.49309999	0.54159999				1.6358	1.62880003
0.49380001	0.54269999				1.63730001	1.62750006
0.4955	0.54299998				1.63409996	1.62399995
0.49169999	0.5431				1.63399994	1.62520003
0.493	0.54009998				1.63569999	1.62390006
0.49180001	0.54180002				1.63750005	1.62650001
0.49599999	0.54290003				1.63510001	1.62539995
					1.63129997	1.62349999
					1.63329995	1.62249994

WT Ethanol - AcetylCoA pH8.8

		2			3	
0.161	0.1627	0.16689999	0.1593	0.2087	0.2017	0.177
0.1619	0.16150001	0.1663	0.1604	0.2008	0.1874	0.17200001
0.1593	0.1629	0.1684	0.1622	0.1998	0.18719999	0.17550001
0.16419999	0.1636	0.1679	0.1621	0.19949999	0.18529999	0.17389999
0.1604	0.1646	0.16779999	0.16159999	0.20479999	0.18269999	0.17389999
0.1596	0.1637	0.1656	0.1657	0.2098	0.1851	0.1735
0.15899999	0.1654	0.1679	0.1626	0.2288	0.1847	0.171
0.16329999	0.16509999	0.1639	0.16150001	0.2114	0.1846	0.1742
0.1644	0.1681	0.16779999	0.1617	0.2115	0.1831	0.1709
0.15880001	0.1652	0.1647	0.16060001	0.2273	0.1874	0.17290001
0.16150001	0.16320001	0.1672	0.16150001	0.2186	0.1849	0.1744
0.1601	0.16429999	0.1673	0.1636	0.2096	0.18870001	0.1741
0.1617	0.1649	0.1683	0.16	0.2159	0.18269999	0.1719
0.15880001	0.16680001	0.1684	0.15979999	0.21179999	0.18449999	0.1751
0.161	0.1655	0.1647	0.1586	0.21170001	0.1868	0.177
0.161	0.16500001	0.1679	0.1626	0.20640001	0.18279999	0.1717
0.1619	0.1666	0.168	0.1584	0.20550001	0.1822	0.1709
0.1611	0.163	0.16509999	0.1565	0.20460001	0.1849	0.1719
0.1603	0.1671	0.1673	0.1586	0.21340001	0.1824	0.1708
0.1601	0.16410001	0.1675	0.16140001	0.20829999	0.18889999	0.1727
0.15790001	0.16670001	0.1648	0.1565	0.213	0.1848	0.1715
0.16230001	0.1618	0.1635	0.156	0.2233	0.1833	0.17299999
0.1608	0.16419999	0.1637	0.1575	0.20460001	0.1855	0.1742
0.1586	0.1663	0.1654	0.1621	0.2062	0.1824	0.1716
0.1591	0.16599999	0.16779999	0.1574	0.2122	0.18610001	0.17460001
0.1587	0.16339999	0.1663	0.15889999	0.20819999	0.1855	0.1732
0.16150001	0.1646	0.16869999	0.15800001	0.20640001	0.183	0.1724
0.15899999	0.16329999	0.1639	0.1578	0.2077	0.18170001	0.1718
0.15889999	0.16230001	0.163	0.1543	0.207	0.18709999	0.17569999
0.1601	0.1648	0.1649	0.1591	0.20649999	0.18539999	0.1744
0.15889999	0.1638	0.16670001	0.1603	0.20739999	0.1841	0.17389999
0.16230001	0.1636	0.1657	0.1585	0.2067	0.1848	0.1727
0.1605	0.16140001	0.1648	0.1583	0.20389999	0.1849	0.1732
0.1594	0.1644	0.16249999	0.1568	0.20720001	0.1925	0.1728
0.15790001	0.1626	0.1679	0.1569	0.2307	0.2076	0.1963
0.1902	0.2694	0.22040001	0.1925	0.25189999	0.2026	0.19840001
0.2034	0.2604	0.2353	0.20739999	0.2723	0.2105	0.20559999
0.2209	0.2721	0.26190001	0.2321	0.2881	0.21430001	0.2114
0.2411	0.29120001	0.2852	0.25580001	0.2992	0.221	0.2273
0.26899999	0.31479999	0.31470001	0.2809	0.31709999	0.23890001	0.2419
0.29089999	0.3466	0.3468	0.31099999	0.34029999	0.25260001	0.26390001
0.32550001	0.38049999	0.38049999	0.34310001	0.3601	0.27329999	0.26969999
0.35479999	0.41460001	0.40700001	0.37670001	0.37760001	0.29730001	0.28510001
0.38769999	0.45050001	0.44490001	0.41370001	0.4025	0.31619999	0.29269999

0.42230001	0.48190001	0.47870001	0.4454	0.42309999	0.33160001	0.3125
0.4549	0.5147	0.51090002	0.4815	0.44240001	0.3515	0.32780001
0.49349999	0.55080003	0.54589999	0.51849997	0.46810001	0.37990001	0.34909999
0.52810001	0.58029997	0.57950002	0.55339998	0.493	0.37959999	0.37650001
0.55970001	0.6124	0.61119998	0.59079999	0.52039999	0.42989999	0.40040001
0.59259999	0.6419	0.64219999	0.62419999	0.537	0.4391	0.42590001
0.62099999	0.66890001	0.6717	0.6591	0.56919998	0.44600001	0.4479
0.65109998	0.69730002	0.6997	0.68739998	0.57309997	0.47729999	0.4707
0.67799997	0.72259998	0.7238	0.71509999	0.60339999	0.50510001	0.4885
0.70169997	0.74900001	0.74650002	0.7353	0.62699997	0.53509998	0.52100003
0.72250003	0.76590002	0.76929998	0.76190001	0.64459997	0.5438	0.55110002
0.74250001	0.78430003	0.78689998	0.78119999	0.65399998	0.5564	0.56690001
0.75880003	0.80549997	0.8064	0.80320001	0.67500001	0.58209997	0.58399999
0.77740002	0.8229	0.82410002	0.82020003	0.71210003	0.6027	0.60280001
0.79470003	0.83850002	0.84100002	0.83639997	0.71700001	0.62470001	0.62599999
0.8107	0.8563	0.85720003	0.85589999	0.73509997	0.63569999	0.63200003
0.8265	0.8671	0.86879998	0.86870003	0.74110001	0.65380001	0.6541
0.83920002	0.88239998	0.88510001	0.8829	0.75349998	0.66610003	0.66970003
0.85369998	0.89740002	0.9016	0.90240002	0.7658	0.6789	0.68510002
0.8653	0.91299999	0.9138	0.91540003	0.77920002	0.68889999	0.69260001
0.88160002	0.92019999	0.9253	0.9242	0.79650003	0.70730001	0.70880002
0.89109999	0.9339	0.93690002	0.94160002	0.80680001	0.71820003	0.72509998
0.90640002	0.9454	0.95020002	0.9526	0.82130003	0.7353	0.73430002
0.9192	0.9598	0.96359998	0.96469998	0.82920003	0.74599999	0.75029999
0.9321	0.97140002	0.97140002	0.97530001	0.82190001	0.7554	0.76630002
0.94370002	0.97820002	0.98269999	0.98680001	0.8427	0.76670003	0.77139997
0.95630002	0.99199998	0.99769998	1.00010002	0.83880001	0.77990001	0.78719997
0.96929997	1.0043	1.00979996	1.01349998	0.86839998	0.79530001	0.80379999
0.97860003	1.01359999	1.02119994	1.02409995	0.86119998	0.8003	0.80470002
0.99159998	1.02530003	1.0316	1.0381	0.88120002	0.81419998	0.81940001
1.00320005	1.03639996	1.04120004	1.04830003	0.89609998	0.8251	0.83469999
1.01489997	1.04929996	1.05219996	1.05649996	0.90789998	0.83319998	0.84210002
1.02639997	1.05610001	1.0632	1.06980002	0.91570002	0.83890003	0.84759998
1.03919995	1.06789994	1.074	1.07990003	0.9224	0.85640001	0.86479998
1.05289996	1.07910001	1.08529997	1.09189999	0.93019998	0.86559999	0.86739999
1.0618	1.08430004	1.09150004	1.10119998	0.94590002	0.8725	0.88529998
1.07350004	1.0977	1.10309994	1.11010003	0.95319998	0.88559997	0.88929999
1.07939994	1.10679996	1.11360002	1.1214	0.96640003	0.8926	0.90679997
1.0927	1.11880004	1.12390006	1.13039994	0.97070003	0.90090001	0.90979999
1.10329998	1.12290001	1.13269997	1.1401	0.98379999	0.91280001	0.92259997
1.1135	1.13469994	1.13810003	1.14740002	0.99059999	0.92729998	0.92760003
1.12689996	1.14559996	1.15050006	1.16069996	0.99760002	0.93229997	0.93879998
1.13419998	1.15250003	1.15910006	1.16869998	1.00469995	0.94239998	0.95179999
1.14470005	1.16069996	1.16830003	1.17649996	1.01789999	0.9483	0.95310003
1.15320003	1.16649997	1.17690003	1.18869996	1.0266	0.96219999	0.96130002
1.16159999	1.17799997	1.18490005	1.19640005	1.0352	0.97030002	0.96939999

1.17349994	1.18799996	1.19430006	1.20410001	1.04120004	0.97259998	0.98030001
1.18280005	1.19669998	1.20490003	1.21730006	1.05410004	0.98460001	0.99349999
1.1882	1.20379996	1.21389997	1.22580004	1.04820001	0.9939	0.99580002
1.20249999	1.21080005	1.21710002	1.22930002	1.07669997	1.00510001	1.00160003
1.20959997	1.21560001	1.22459996	1.23730004	1.07889998	1.01289999	1.01300001
1.21459997	1.22309995	1.23399997	1.24530005	1.08229995	1.01839995	1.02740002
1.22860003	1.23430002	1.24549997	1.25489998	1.0927	1.02779996	1.03310001
1.23810005	1.24240005	1.25220001	1.26370001	1.09949994	1.03729999	1.03209996
1.2464	1.24909997	1.25899994	1.27359998	1.10539997	1.04050004	1.04079998
1.25129998	1.25399995	1.26559997	1.27649999	1.10969996	1.05859995	1.05009997
1.26020002	1.2658	1.27450001	1.28579998	1.12909997	1.05400002	1.04910004
1.27190006	1.26789999	1.27339995	1.28929996	1.11860001	1.0668	1.05920005
1.27699995	1.27900004	1.28960001	1.30060005	1.13670003	1.07500005	1.07959998
1.28559995	1.28499997	1.29330003	1.30780005	1.14040005	1.08519995	1.07659996
1.29289997	1.29180002	1.30069995	1.31210005	1.14750004	1.08749998	1.07969999
1.30180001	1.2974	1.30550003	1.3233	1.15390003	1.09669995	1.09609997
1.30579996	1.30410004	1.31400001	1.32449996	1.15699995	1.10309994	1.0948
1.3154	1.31159997	1.32099998	1.33350003	1.16559994	1.10450006	1.10720003
1.32130003	1.31509995	1.3233	1.33720005	1.17480004	1.11699998	1.11530006
1.32980001	1.3154	1.32939994	1.34500003	1.17569995	1.12440002	1.1135
1.33449996	1.32869995	1.33640003	1.34809995	1.19120002	1.12909997	1.12390006
1.34280002	1.33459997	1.34220004	1.35839999	1.19169998	1.14300001	1.13160002
1.34949994	1.34109998	1.35039997	1.36580002	1.19700003	1.1437	1.12899995
1.35710001	1.34739995	1.35679996	1.36940002	1.20510006	1.14690006	1.14059997
1.35959995	1.34959996	1.35810006	1.3721	1.21130002	1.14909995	1.14600003
1.36440003	1.35669994	1.36670005	1.38059998	1.21469998	1.16630006	1.15040004
1.37849998	1.36059999	1.3721	1.38600004	1.21659994	1.16530001	1.15289998
1.38090003	1.36469996	1.37600005	1.38909996	1.22679996	1.17809999	1.16729999
1.38779998	1.37259996	1.38170004	1.39540005	1.23710001	1.17760003	1.16270006
1.39380002	1.37629998	1.38569999	1.39709997	1.23599994	1.18359995	1.16589999
1.40069997	1.38129997	1.3901	1.40400004	1.25080001	1.18589997	1.17610002
1.40219998	1.38460004	1.39409995	1.40929997	1.24919999	1.19589996	1.18920004
1.40769994	1.39059997	1.40120006	1.41729999	1.25020003	1.20449996	1.18289995
1.41830003	1.39600003	1.40209997	1.41840005	1.24790001	1.20220006	1.18739998
1.42040002	1.39839995	1.40919995	1.42369998	1.26329994	1.20650005	1.19079995
1.42809999	1.40970004	1.41439998	1.4296	1.27390003	1.21519995	1.20070004
1.43229997	1.40960002	1.41729999	1.43330002	1.28170002	1.21619999	1.20270002
1.43439996	1.41209996	1.42320001	1.4368	1.2737	1.22319996	1.21039999
1.44420004	1.41760004	1.42620003	1.44019997	1.28279996	1.22790003	1.21070004
1.44719994	1.42009997	1.43190002	1.44719994	1.29190004	1.23039997	1.21420002
1.45290005	1.42929995	1.43690002	1.45009995	1.28960001	1.23689997	1.21889997
1.45679998	1.42900002	1.43780005	1.45410001	1.29139996	1.24230003	1.22500002
1.46370006	1.43809998	1.44500005	1.45910001	1.30639994	1.25160003	1.2385
1.46519995	1.43630004	1.44420004	1.45889997	1.30410004	1.25209999	1.227
1.46850002	1.44410002	1.45109999	1.4677	1.30560005	1.25320005	1.2313
1.46790004	1.44449997	1.45150006	1.46630001	1.31140006	1.26440001	1.2385

1.47749996	1.44679999	1.45899999	1.47109997	1.31309998	1.26189995	1.23959994
1.48269999	1.45469999	1.46280003	1.47599995	1.32410002	1.27559996	1.24419999
1.48979998	1.45439994	1.46539998	1.48010004	1.32290006	1.27610004	1.25590003
1.49220002	1.45920002	1.47130001	1.48469996	1.32560003	1.28209996	1.26129997
1.495	1.46599996	1.46870005	1.48479998	1.32729995	1.27269995	1.25530005
1.49699998	1.46379995	1.47000003	1.4878	1.33829999	1.2845	1.25569999
1.5029	1.46990001	1.47669995	1.49189997	1.33560002	1.28919995	1.26750004
1.50310004	1.47549999	1.48259997	1.49339998	1.34640002	1.29149997	1.26440001
1.51250005	1.47370005	1.48319995	1.49960005	1.34630001	1.29419994	1.26900005
1.51250005	1.47880006	1.48909998	1.50230002	1.34619999	1.31149995	1.2802
1.51090002	1.48339999	1.48740005	1.50160003	1.36000001	1.30239999	1.27970004
1.52119994	1.48539996	1.48720002	1.50320005	1.35889995	1.31110001	1.27699995
1.528	1.48580003	1.49489999	1.50820005	1.3635	1.31210005	1.28840005
1.52880001	1.49179995	1.49829996	1.5151	1.37220001	1.31379998	1.28559995
1.52760005	1.49380004	1.49839997	1.51339996	1.36839998	1.31930006	1.28799999
1.53670001	1.50059998	1.50779998	1.52180004	1.37730002	1.31340003	1.29589999
1.53729999	1.50230002	1.50699997	1.52069998	1.38320005	1.32029998	1.30200005
1.53629994	1.50059998	1.50730002	1.52090001	1.38489997	1.33080006	1.29499996
1.54120004	1.49989998	1.51010001	1.5237	1.38670003	1.33430004	1.29639995
1.54519999	1.50890005	1.51489997	1.52999997	1.38730001	1.32529998	1.29859996
1.5503	1.51329994	1.51520002	1.528	1.38370001	1.33780003	1.30159998
1.551	1.51199996	1.51820004	1.53069997	1.39069998	1.33840001	1.30949998
1.54779994	1.51859999	1.52499998	1.53770006	1.38999999	1.33829999	1.30630004
1.55939996	1.51370001	1.52040005	1.53349996	1.38489997	1.34360003	1.30669999
1.55929995	1.5165	1.52409995	1.5381	1.39479995	1.34329998	1.3089
1.5618	1.52419996	1.52719998	1.53980005	1.39579999	1.34329998	1.31770003
1.56280005	1.52590001	1.53129995	1.5474	1.39719999	1.34609997	1.32260001
1.56739998	1.52859998	1.53180003	1.54589999	1.39649999	1.34959996	1.31470001
1.5675	1.53110003	1.53499997	1.54560006	1.39760005	1.35099995	1.31879997
1.57309997	1.52890003	1.5359	1.54949999	1.39950001	1.35739994	1.32860005
1.57260001	1.53489995	1.53859997	1.55260003	1.41100001	1.36409998	1.3283
1.57770002	1.53620005	1.54050004	1.55250001	1.41439998	1.36300004	1.33010006
1.5747	1.53670001	1.53890002	1.55340004	1.41760004	1.37030005	1.32860005
1.57669997	1.53859997	1.54400003	1.55550003	1.41530001	1.36479998	1.33150005
1.58459997	1.54170001	1.546	1.55910003	1.41849995	1.36489999	1.33990002
1.58790004	1.54100001	1.54419994	1.55900002	1.42400002	1.3779	1.32720006
1.58420002	1.54159999	1.54359996	1.55760002	1.42060006	1.37119997	1.33270001
1.59230006	1.54419994	1.55060005	1.56289995	1.42359996	1.37709999	1.33630002
1.59070003	1.54869998	1.54929996	1.56159997	1.43079996	1.38590002	1.34350002
1.59329998	1.54890001	1.55499995	1.56819999	1.42620003	1.37979996	1.33790004
1.59389997	1.55379999	1.55359995	1.57009995	1.42850006	1.3822	1.34440005
1.5927	1.54849994	1.55350006	1.57050002	1.4253	1.38160002	1.34739995
1.60140002	1.55579996	1.55589998	1.5697	1.43519998	1.38080001	1.34080005
1.59979999	1.5539	1.55490005	1.56900001	1.43299997	1.38429999	1.34920001
1.59909999	1.56120002	1.56130004	1.57299995	1.43239999	1.3872	1.3563
1.60370004	1.55700004	1.56190002	1.57410002	1.4332	1.39600003	1.347

1.60389996	1.56070006	1.56429994	1.57669997	1.44149995	1.38859999	1.35350001
1.6085	1.56289995	1.56110001	1.57669997	1.44299996	1.39059997	1.35020006
1.60580003	1.56099999	1.56579995	1.58019996	1.4382	1.39810002	1.36500001
1.60590005	1.56640005	1.56760001	1.57959998	1.44700003	1.40499997	1.35619998
1.60950005	1.56630003	1.56770003	1.58319998	1.44079995	1.39460003	1.35549998
1.61600006	1.57000005	1.56840003	1.57840002	1.44210005	1.40540004	1.36059999
1.61230004	1.56570005	1.56819999	1.58389997	1.45819998	1.40310001	1.35909998
1.61720002	1.56910002	1.57169998	1.58630002	1.45469999	1.4095	1.36459994
1.61679995	1.57159996	1.57490003	1.58879995	1.45319998	1.40139997	1.35780001
1.62	1.57509995	1.57589996	1.58879995	1.44930005	1.40120006	1.36370003
1.62170005	1.57149994	1.57350004	1.58650005	1.4526	1.4066	1.37880003

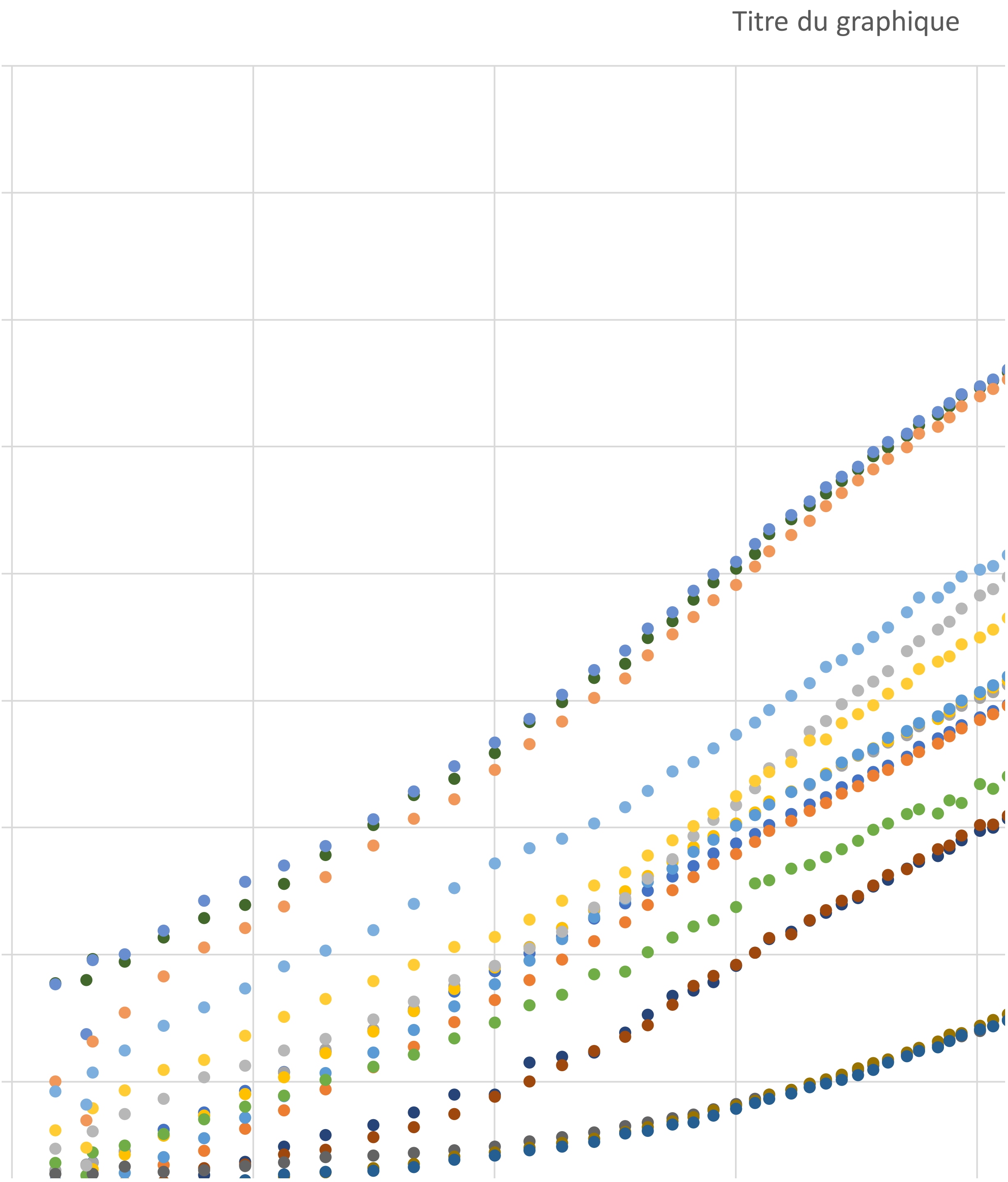

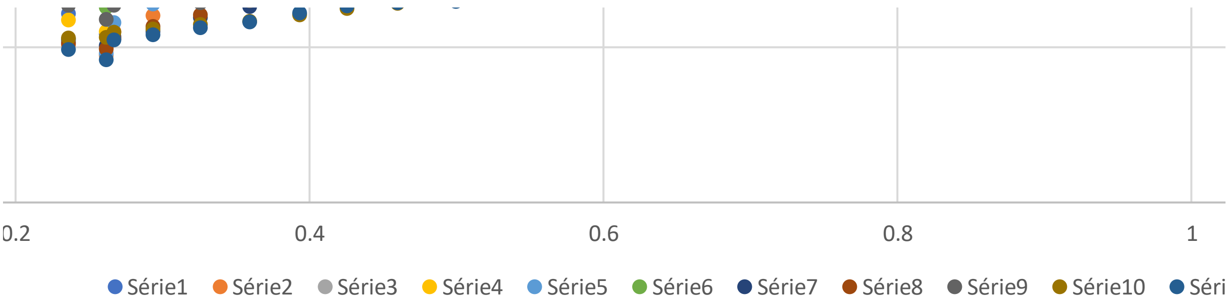

[illegible]	[illegible]	[illegible]	[illegible]	[illegible]	[illegible]	[illegible]
1.65429997	1.6085	1.60309994	1.61810005	1.49220002	1.44669998	1.39810002
1.65699995	1.61000001	1.60739994	1.61670005	1.4892	1.44280005	1.39409995
1.65509999	1.6085	1.60469997	1.61520004	1.48930001	1.44599998	1.39929998
1.65530002	1.60780001	1.60660005	1.61989999	1.48749995	1.44659996	1.38929999
1.65509999	1.61530006	1.61010003	1.62100005	1.50150001	1.44599998	1.40009999
1.66009998	1.60860002	1.6063	1.61919999	1.49059999	1.44749999	1.3973
1.66110003	1.61020005	1.60699999	1.61870003	1.50750005	1.45150006	1.39709997
1.66129994	1.61240005	1.60599995	1.6142	1.50880003	1.45249999	1.40499997
1.66040003	1.60930002	1.60500002	1.61810005	1.49800003	1.44809997	1.40180004
1.66229999	1.60979998	1.60710001	1.62220001	1.49989998	1.45229995	1.39999998
1.66059995	1.60909998	1.60860002	1.61849999	1.49360001	1.44519997	1.39750004
1.65999997	1.61220002	1.60730004	1.61800003	1.49950004	1.44930005	1.39660001
1.66050005	1.61329997	1.61090004	1.62049997	1.50020003	1.45120001	1.39590001
1.65970004	1.61319995	1.60590005	1.61520004	1.49469995	1.45099998	1.39929998
1.66120005	1.6099	1.60720003	1.61769998	1.49899995	1.45640004	1.39709997
1.66349995	1.60749996	1.60699999	1.61880004	1.49860001	1.45369995	1.40170002
1.65999997	1.61380005	1.61059999	1.62230003	1.50349998	1.45150006	1.39750004
1.66139996	1.61539996	1.60679996	1.61600006	1.50580001	1.44799995	1.39919996
1.66199994	1.61259997	1.60800004	1.62119997	1.49590003	1.44949996	1.39540005
1.66369998	1.6142	1.60839999	1.61969995	1.50829995	1.45299995	1.40540004
1.66100001	1.61300004	1.60800004	1.61930001	1.51069999	1.44910002	1.40030003
1.66670001	1.61670005	1.6128	1.62199998	1.50100005	1.44930005	1.40409994
1.66499996	1.61479998	1.61339998	1.61960006	1.50839996	1.45109999	1.40040004
1.66240001	1.61759996	1.61080003	1.62030005	1.50759995	1.45519996	1.39569998
1.66470003	1.61670005	1.61670005	1.62740004	1.50129998	1.45140004	1.39579999
1.66199994	1.61530006	1.60669994	1.61720002	1.50129998	1.45529997	1.40139997
1.66349995	1.61730003	1.61039996	1.62150002	1.50829995	1.44939995	1.40260005
1.66999996	1.61370003	1.60860002	1.61960006	1.50150001	1.45130002	1.40009999
1.66439998	1.61720002	1.61329997	1.62380004	1.49520004	1.45200002	1.39579999
1.66139996	1.61800003	1.61380005	1.62419999	1.49880004	1.46220005	1.39699996
1.66330004	1.61699998	1.60839999	1.61839998	1.5036	1.44840002	1.39779997
1.66499996	1.62080002	1.61249995	1.6221	1.49909997	1.4562	1.39849997
1.66709995	1.61450005	1.61059999	1.61989999	1.5036	1.46150005	1.3987
1.6645	1.61650002	1.61170006	1.62160003	1.50870001	1.45210004	1.40040004
1.66330004	1.61679995	1.61070001	1.62249994	1.50139999	1.4526	1.39839995
1.66569996	1.61800003	1.61269999	1.61940002	1.50199997	1.45640004	1.40020001
1.66980004	1.61819994	1.61459994	1.62469995	1.51300001	1.45609999	1.40170002
1.66569996	1.61800003	1.61189997	1.62090003	1.51370001	1.46000004	1.39900005

1.66709995	1.61489999	1.61220002	1.62539995	1.50759995	1.45920002	1.3994
1.66919994	1.61679995	1.61189997	1.62339997	1.51119995	1.45879996	1.39909995
1.66359997	1.61790001	1.61029994	1.6171	1.50689995	1.45599997	1.39849997
1.66559994	1.61570001	1.61399996	1.62380004	1.49960005	1.45770001	1.40429997
1.6688	1.62179995	1.61580002	1.62370002	1.50969994	1.45799994	1.40369999
1.66579998	1.61510003	1.61039996	1.62010002	1.50380003	1.45439994	1.39950001
1.6652	1.61749995	1.61230004	1.62360001	1.50880003	1.45749998	1.39520001
1.6674	1.62310004	1.61479998	1.62349999	1.50160003	1.45910001	1.41069996
1.66840005	1.61909997	1.61010003	1.62240005	1.50899994	1.45500004	1.40869999
1.66680002	1.61829996	1.61549997	1.62380004	1.50179994	1.45669997	1.403
1.66589999	1.61749995	1.60800004	1.62109995	1.50610006	1.45270002	1.39750004
1.66550004	1.61380005	1.61049998	1.61839998	1.50189996	1.45580006	1.39849997
1.66400003	1.62059999	1.61539996	1.6243	1.51010001	1.44959998	1.40110004
1.66680002	1.62	1.61119998	1.62310004	1.50320005	1.45930004	1.40079999
1.66110003	1.61909997	1.61650002	1.62460005	1.505	1.46300006	1.40269995
1.66340005	1.61730003	1.60979998	1.61749995	1.50119996	1.45650005	1.39999998
1.66919994	1.61839998	1.61199999	1.61930001	1.50639999	1.45290005	1.40030003
1.67069995	1.61730003	1.61000001	1.61720002	1.50049996	1.4562	1.39909995
1.66799998	1.62230003	1.61409998	1.62460005	1.50139999	1.46099997	1.40129995
1.66750002	1.61469996	1.61170006	1.62249994	1.50329995	1.46459997	1.40219998
1.66419995	1.62020004	1.6135	1.62	1.50380003	1.45599997	1.40279996
1.66270006	1.61849999	1.61479998	1.61960006	1.50569999	1.45739996	1.3987
1.66719997	1.61670005	1.61179996	1.62049997	1.50039995	1.46370006	1.40880001
1.66579998	1.61899996	1.61029994	1.61829996	1.50080001	1.45519996	1.39960003
1.66410005	1.61430001	1.61029994	1.62	1.50409997	1.45819998	1.39960003
1.66480005	1.61759996	1.61300004	1.62310004	1.50539994	1.45770001	1.40279996
1.66670001	1.61810005	1.61070001	1.61930001	1.50199997	1.46000004	1.40090001
1.66820002	1.61539996	1.60829997	1.61839998	1.49600005	1.46019995	1.40289998
1.66480005	1.61730003	1.60930002	1.61790001	1.51380002	1.45669997	1.39909995
1.66600001	1.61699998	1.61600006	1.62440002	1.50489998	1.45809996	1.39950001
1.66540003	1.62010002	1.61259997	1.62240005	1.50409997	1.45940006	1.40419996
1.66610003	1.61500001	1.60800004	1.61660004	1.5086	1.45299995	1.39619994
1.66569996	1.61849999	1.61080003	1.62090003	1.49950004	1.45879996	1.403
1.66729999	1.61319995	1.61090004	1.61670005	1.50139999	1.46239996	1.40460002
1.66349995	1.6178	1.61310005	1.62030005	1.50950003	1.46389997	1.39760005
1.66639996	1.61679995	1.61020005	1.61819994	1.50150001	1.45739996	1.40209997
1.66670001	1.61539996	1.60699999	1.61759996	1.50269997	1.45179999	1.39849997
1.66340005	1.62080002	1.61679995	1.62160003	1.50569999	1.45840001	1.40359998
1.66639996	1.61880004	1.61119998	1.61759996	1.50520003	1.45679998	1.39970005
1.66330004	1.61059999	1.60619998	1.61670005	1.50020003	1.45819998	1.40100002
1.66649997	1.61399996	1.60769999	1.61769998	1.50059998	1.4569	1.39629996
1.66069996	1.61870003	1.61049998	1.61889994	1.49679995	1.46130002	1.39649999
1.66760004	1.62	1.6128	1.61950004	1.50489998	1.46010005	1.40040004
1.66680002	1.61819994	1.61290002	1.62300003	1.51180005	1.45829999	1.39610004
1.66579998	1.6128	1.61099994	1.62	1.51209998	1.45920002	1.39670002
1.66649997	1.6178	1.60730004	1.61520004	1.51110005	1.45959997	1.3987

1.66369998	1.61230004	1.60790002	1.61570001	1.50399995	1.45459998	1.39279997
1.66600001	1.61590004	1.61070001	1.62129998	1.51139998	1.45829999	1.39579999
1.6645	1.61790001	1.61010003	1.61740005	1.50899994	1.45790005	1.39789999
1.6652	1.61199999	1.60810006	1.61590004	1.50399995	1.45630002	1.39950001
1.66670001	1.61919999	1.60749996	1.61800003	1.50080001	1.45949996	1.40460002
1.66079998	1.61459994	1.60619998	1.61500001	1.50639999	1.45630002	1.40380001
1.66600001	1.61459994	1.61000001	1.61829996	1.50139999	1.4605	1.39610004
1.66369998	1.61960006	1.61160004	1.6178	1.50709999	1.46039999	1.398
1.66279995	1.61370003	1.60749996	1.61619997	1.50890005	1.4576	1.39559996
1.66289997	1.61310005	1.60749996	1.61860001	1.50279999	1.45829999	1.39750004
1.66139996	1.61570001	1.60609996	1.61210001	1.5086	1.46329999	1.39090002
1.66139996	1.61660004	1.60839999	1.61530006	1.50310004	1.45459998	1.39619994
1.6652	1.6142	1.60720003	1.61670005	1.51600003	1.45720005	1.39890003
1.66340005	1.61580002	1.60959995	1.61769998	1.50510001	1.45529997	1.39950001
1.66299999	1.61500001	1.60679996	1.61510003	1.50559998	1.46229994	1.3987
1.66299999	1.61500001	1.61080003	1.61889994	1.50039995	1.46010005	1.39929998
1.6609	1.61699998	1.60699999	1.61189997	1.50469995	1.46529996	1.40450001
1.65929997	1.61029994	1.60800004	1.61360002	1.50520003	1.45910001	1.40190005
1.65810001	1.61539996	1.60969996	1.61329997	1.50230002	1.4576	1.39670002
1.66509998	1.61520004	1.60650003	1.61409998	1.49520004	1.45410001	1.39349997
1.65939999	1.61629999	1.60539997	1.61039996	1.51760006	1.45749998	1.39479995
1.65900004	1.61629999	1.61179996	1.61730003	1.50030005	1.46500003	1.39859998
1.66120005	1.61500001	1.60870004	1.61339998	1.50209999	1.45930004	1.40289998
1.66180003	1.60979998	1.6063	1.61389995	1.48769999	1.45640004	1.3951
1.6602	1.61080003	1.60440004	1.61370003	1.49749994	1.46010005	1.39810002
1.66250002	1.61210001	1.60880005	1.6128	1.50989997	1.45220006	1.39400005
1.65820003	1.60909998	1.60430002	1.61290002	1.49590003	1.45360005	1.39820004
1.65840006	1.61119998	1.6056	1.61189997	1.49399996	1.45309997	1.39979994
1.66170001	1.61109996	1.60459995	1.61290002	1.50349998	1.45640004	1.39170003
1.65719998	1.61179996	1.60640001	1.61080003	1.49890006	1.46169996	1.3944
1.65670002	1.61339998	1.60880005	1.61580002	1.49919999	1.45420003	1.3901
1.65610003	1.60810006	1.60109997	1.61160004	1.51110005	1.45309997	1.39129996
1.66050005	1.61129999	1.60430002	1.6128	1.50250006	1.45550001	1.39209998
1.65989995	1.61389995	1.60769999	1.61039996	1.51139998	1.45739996	1.39429998
1.65869999	1.61160004	1.602	1.60650003	1.49699998	1.44749999	1.39069998
1.65499997	1.60819995	1.60440004	1.61119998	1.50639999	1.4576	1.39520001
1.65569997	1.60969996	1.60640001	1.6135	1.50610006	1.4562	1.39189994
1.65450001	1.61310005	1.60549998	1.60860002	1.50489998	1.44980001	1.39670002
1.65680003	1.61189997	1.6049	1.61070001	1.50870001	1.45510006	1.39629996
1.65789998	1.61319995	1.60889995	1.60959995	1.50670004	1.45369995	1.39549994
1.65919995	1.60889995	1.60469997	1.61129999	1.50469995	1.45439994	1.39629996
1.65820003	1.60549998	1.60160005	1.60800004	1.48360002	1.45410001	1.39779997
1.65620005	1.61020005	1.60160005	1.60739994	1.49660003	1.45679998	1.39349997
1.65409994	1.60870004	1.59969997	1.60619998	1.49720001	1.44869995	1.39470005
1.65209997	1.6085	1.60529995	1.61099994	1.50709999	1.45850003	1.39409995
1.65460002	1.61300004	1.60500002	1.60889995	1.49740005	1.45340002	1.39330006

1.65269995	1.60720003	1.59899998	1.60570002	1.49969995	1.45039999	1.39619994
1.65799999	1.6085	1.60220003	1.60909998	1.4964	1.45009995	1.3901
1.65450001	1.61059999	1.60370004	1.6092	1.49059999	1.45790005	1.39119995
1.65760005	1.60570002	1.59990001	1.60660005	1.4971	1.45190001	1.39489996
1.65680003	1.60810006	1.60459995	1.61090004	1.49419999	1.45299995	1.3908
1.65629995	1.60570002	1.59759998	1.60469997	1.4964	1.45109999	1.3901
1.65170002	1.60710001	1.60249996	1.60710001	1.50680006	1.45720005	1.3901
1.65040004	1.60619998	1.60099995	1.60650003	1.48819995	1.45340002	1.39059997
1.65600002	1.60619998	1.59920001	1.60450006	1.48559999	1.45369995	1.39380002
1.65260005	1.60580003	1.60210001	1.60710001	1.51600003	1.46099997	1.39839995
1.65409994	1.60650003	1.6013	1.60640001	1.48119998	1.45169997	1.39390004
1.65579998	1.60599995	1.59969997	1.60609996	1.49310005	1.45060003	1.39180005
1.65269995	1.60730004	1.59949994	1.60580003	1.49810004	1.45290005	1.39049995
1.64880002	1.60689998	1.6006	1.60699999	1.50409997	1.45270002	1.39649999
1.65429997	1.60609996	1.60249996	1.60599995	1.51199996	1.44519997	1.39559996
1.65460002	1.60469997	1.59850001	1.60370004	1.50109994	1.44959998	1.39180005
1.65330005	1.60730004	1.59920001	1.60309994	1.49909997	1.45169997	1.39260006
1.65170002	1.60430002	1.60049999	1.60570002	1.50189996	1.44739997	1.38909996
1.65250003	1.60500002	1.5984	1.60459995	1.49890006	1.45389998	1.38979995
1.65149999	1.60450006	1.59969997	1.60259998	1.49810004	1.45120001	1.38709998
1.64789999	1.60350001	1.59679997	1.60080004	1.49100006	1.44369996	1.38520002
1.65059996	1.60549998	1.59870005	1.60249996	1.49179995	1.46150005	1.39310002
1.65120006	1.60099995	1.59500003	1.60140002	1.49269998	1.45659995	1.38139999
1.65020001	1.60599995	1.602	1.6056	1.50779998	1.45130002	1.38999999
1.65050006	1.60210001	1.59350002	1.59809995	1.50489998	1.45229995	1.39030004
1.65139997	1.60010004	1.59679997	1.60109997	1.49839997	1.449	1.38859999
1.65170002	1.60210001	1.5977	1.602	1.50139999	1.44340003	1.38839996
1.648	1.60249996	1.60029995	1.60259998	1.50090003	1.44910002	1.38859999
1.64690006	1.60599995	1.60029995	1.59969997	1.48679996	1.44459999	1.38709998
1.64830005	1.60119998	1.5927	1.59990001	1.49409997	1.44739997	1.38479996
1.64709997	1.60140002	1.59879994	1.60210001	1.49969995	1.44700003	1.39230001
1.65059996	1.60300004	1.59630001	1.59829998	1.48650002	1.44770002	1.39100003
1.64709997	1.59630001	1.59140003	1.59640002	1.49979997	1.43780005	1.38779998
1.64900005	1.60370004	1.59809995	1.60169995	1.49810004	1.45130002	1.38590002
1.64320004	1.60189998	1.59819996	1.59979999	1.49000001	1.44679999	1.38919997
1.64670002	1.60259998	1.59560001	1.59850001	1.50160003	1.44019997	1.37849998
1.65059996	1.59739995	1.59350002	1.59660006	1.48930001	1.44799995	1.38680005
1.64230001	1.59850001	1.59389997	1.59630001	1.48230004	1.44749999	1.37660003
1.64839995	1.59730005	1.59580004	1.59749997	1.49790001	1.44649994	1.38440001
1.64289999	1.59309995	1.58819997	1.59249997	1.49730003	1.45079994	1.38730001
1.64559996	1.59870005	1.59430003	1.60090005	1.49109995	1.44480002	1.38370001
1.65090001	1.59549999	1.59230006	1.59650004	1.49940002	1.449	1.38590002
1.64310002	1.59609997	1.5898	1.5948	1.47969997	1.44729996	1.38110006
1.64310002	1.59949994	1.59360003	1.59500003	1.49549997	1.43970001	1.38429999
1.64709997	1.59519994	1.59230006	1.59360003	1.49129999	1.4332	1.38119996
1.64610004	1.59809995	1.5898	1.59319997	1.49940002	1.45200002	1.38329995

1.64489996	1.5948	1.5934	1.59759998	1.49070001	1.44749999	1.38230002
1.64520001	1.5941	1.59019995	1.59070003	1.48559999	1.44579995	1.38989997
1.63950002	1.59739995	1.59440005	1.59780002	1.49049997	1.44710004	1.38610005
1.64240003	1.60179996	1.59379995	1.59809995	1.49059999	1.44200003	1.38479996
1.64260006	1.59200001	1.59010005	1.59519994	1.49829996	1.449	1.38670003
1.64479995	1.59590006	1.59379995	1.597	1.49909997	1.44749999	1.38269997
1.64540005	1.59549999	1.59010005	1.59189999	1.49820006	1.45510006	1.38880002
1.63919997	1.59200001	1.58749998	1.59280002	1.48389995	1.44410002	1.3829
1.64349997	1.59200001	1.59200001	1.59300005	1.49100006	1.44260001	1.38629997
1.63970006	1.59689999	1.59159994	1.59430003	1.49590003	1.43889999	1.38170004
1.64209998	1.59249997	1.58860004	1.58870006	1.49170005	1.44239998	1.39160001
1.63689995	1.59319997	1.58710003	1.59130001	1.49039996	1.44330001	1.38469994
1.63989997	1.5941	1.59459996	1.59259999	1.49530005	1.43939996	1.38549995
1.63940001	1.59360003	1.58729994	1.59060001	1.48370004	1.44140005	1.38510001
1.64129996	1.58879995	1.5891	1.59280002	1.49779999	1.44210005	1.37810004
1.63859999	1.59360003	1.58860004	1.59089994			
1.64059997	1.59169996	1.58630002	1.59169996			
1.64020002	1.59360003	1.58959997	1.59060001			
1.63680005	1.59280002	1.58780003	1.58780003			
1.63849998	1.58449996	1.58449996	1.58850002			
1.6372	1.58949995	1.58599997	1.59070003			
1.63709998	1.58930004	1.58440006	1.58720005			
1.63670003	1.58870006	1.58860004	1.5898			
1.63810003	1.59039998	1.58340001	1.5855			
1.63670003	1.59200001	1.58640003	1.5905			
	1.58969998	1.58449996	1.58679998			

Mutant 446-449del Ethanol - AcetylCoA pH8.8

	1			2		
0.16	0.1601	0.16069999	0.1591	0.1605	0.1647	0.1777
0.162	0.1644	0.1638	0.1582	0.1583	0.1582	0.18719999
0.1611	0.164	0.1654	0.15449999	0.15539999	0.15270001	0.1824
0.1601	0.161	0.1613	0.1559	0.1556	0.1543	0.1973
0.1613	0.1629	0.1605	0.1576	0.1585	0.15809999	0.1865
0.1612	0.1644	0.1602	0.15719999	0.1577	0.1577	0.184
0.15970001	0.15970001	0.1621	0.1557	0.1552	0.15880001	0.1822
0.1604	0.16249999	0.16069999	0.1575	0.15710001	0.1602	0.18359999
0.16159999	0.1608	0.1605	0.15790001	0.15710001	0.15710001	0.1846
0.1613	0.16230001	0.16760001	0.1564	0.1539	0.15530001	0.1857
0.15899999	0.1602	0.1653	0.15880001	0.1573	0.1578	0.1864
0.161	0.16329999	0.1638	0.1532	0.1552	0.1515	0.1864
0.1604	0.1603	0.1613	0.1573	0.156	0.1531	0.183
0.1603	0.1611	0.1595	0.1532	0.15449999	0.1531	0.185
0.1603	0.1617	0.16060001	0.155	0.155	0.1569	0.18260001
0.16159999	0.1622	0.1603	0.1558	0.1541	0.15459999	0.20100001
0.1584	0.16329999	0.16240001	0.156	0.15350001	0.1539	0.19760001
0.16150001	0.162	0.1601	0.1565	0.1557	0.15369999	0.1877
0.15979999	0.1613	0.1585	0.15549999	0.155	0.1539	0.1829
0.16060001	0.1622	0.1601	0.1558	0.1548	0.15260001	0.18089999
0.1601	0.1627	0.1609	0.15710001	0.15710001	0.153	0.1805
0.1587	0.1609	0.1618	0.15700001	0.15629999	0.1532	0.18009999
0.15880001	0.1627	0.1608	0.15790001	0.1539	0.1534	0.1849
0.1585	0.1595	0.1617	0.1548	0.15350001	0.1564	0.18629999
0.161	0.1629	0.16410001	0.15549999	0.1549	0.1534	0.182
0.16159999	0.16150001	0.1648	0.156	0.15629999	0.15090001	0.1806
0.16	0.1613	0.1618	0.15809999	0.15530001	0.15629999	0.1873
0.1608	0.1637	0.16339999	0.1568	0.15449999	0.15350001	0.1869
0.1602	0.1611	0.1637	0.1569	0.15440001	0.1534	0.1867
0.15970001	0.15970001	0.16240001	0.15459999	0.1569	0.1534	0.18610001
0.1593	0.1626	0.1613	0.1556	0.1547	0.1506	0.1815
0.1586	0.1604	0.1647	0.15790001	0.1552	0.15440001	0.1953
0.1596	0.16320001	0.162	0.15700001	0.1539	0.1547	0.19239999
0.15989999	0.1626	0.16339999	0.1543	0.1551	0.15549999	0.18340001
0.1593	0.1602	0.1627	0.1565	0.1557	0.15530001	0.26800001
0.2362	0.2131	0.1842	0.56019998	0.47440001	0.3387	0.26750001
0.25440001	0.2123	0.1974	0.5546	0.55339998	0.40040001	0.29449999
0.25440001	0.2194	0.2097	0.59280002	0.59140003	0.46349999	0.32179999
0.2658	0.2243	0.2164	0.58899999	0.60039997	0.5086	0.34869999
0.25780001	0.2296	0.22570001	0.62709999	0.63800001	0.56559998	0.373
0.2604	0.2339	0.2332	0.65780002	0.68449998	0.611	0.40700001
0.26710001	0.242	0.2446	0.67830002	0.71429998	0.6415	0.4249
0.27289999	0.25049999	0.2536	0.71100003	0.74010003	0.67570001	0.44870001
0.2807	0.2568	0.25799999	0.75700003	0.77109998	0.722	0.46709999

0.28349999	0.2638	0.25920001	0.80409998	0.81349999	0.77149999	0.4975
0.28749999	0.2705	0.2651	0.85110003	0.85710001	0.81410003	0.52630001
0.29139999	0.28189999	0.27689999	0.87709999	0.89649999	0.8448	0.55980003
0.29719999	0.2886	0.28389999	0.91759998	0.93430001	0.89120001	0.58219999
0.3062	0.2958	0.292	0.96649998	0.97149998	0.93150002	0.60960001
0.31209999	0.30309999	0.2974	0.99739999	1.00940001	0.9673	0.63599998
0.3202	0.31020001	0.3048	1.03610003	1.04849994	1.00419998	0.67379999
0.3301	0.3204	0.31830001	1.05840003	1.07869995	1.03470004	0.68870002
0.33570001	0.32780001	0.3224	1.09889996	1.11339998	1.0711	0.71929997
0.34240001	0.3373	0.3321	1.12520003	1.13940001	1.10459995	0.7507
0.34799999	0.34400001	0.33590001	1.15919995	1.17289996	1.1322	0.78680003
0.35640001	0.35510001	0.34619999	1.18610001	1.19889998	1.15789998	0.81239998
0.36500001	0.36219999	0.35710001	1.20790005	1.2191	1.18270004	0.83590001
0.37270001	0.37	0.36590001	1.2313	1.24699998	1.21140003	0.86199999
0.37959999	0.37920001	0.3732	1.26259995	1.26999998	1.23549998	0.8937
0.3867	0.38710001	0.38139999	1.28579998	1.29260004	1.26090002	0.91509998
0.39579999	0.39660001	0.3908	1.30710006	1.31389999	1.28349996	0.95109999
0.40099999	0.4034	0.39660001	1.32669997	1.33650005	1.30659997	0.96789998
0.4077	0.4109	0.4023	1.34630001	1.35319996	1.32739997	0.99440002
0.41729999	0.42070001	0.41049999	1.36399996	1.36819994	1.34710002	1.0158
0.42480001	0.42950001	0.41870001	1.38530004	1.39139998	1.36479998	1.02980006
0.43180001	0.43509999	0.43000001	1.3994	1.40769994	1.38119996	1.04620004
0.4425	0.4456	0.43959999	1.41760004	1.42089999	1.39900005	1.07799995
0.44980001	0.45289999	0.4481	1.43429995	1.44029999	1.42060006	1.0941
0.45730001	0.46290001	0.45410001	1.45009995	1.45449996	1.43149996	1.11160004
0.46599999	0.47389999	0.4637	1.46360004	1.4684	1.44620001	1.12450004
0.47119999	0.4761	0.47310001	1.48140001	1.48310006	1.46340001	1.14530003
0.4799	0.48769999	0.48210001	1.49300003	1.49520004	1.47940004	1.16559994
0.48800001	0.49720001	0.4867	1.50390005	1.50580001	1.49090004	1.176
0.49689999	0.50599998	0.49590001	1.51760006	1.52129996	1.50629997	1.19490004
0.505	0.5122	0.5036	1.52989995	1.52849996	1.51859999	1.21000004
0.51020002	0.51899999	0.51389998	1.54139996	1.54429996	1.5237	1.2227
0.51920003	0.52630001	0.51859999	1.55280006	1.55350006	1.54400003	1.22800004
0.52450001	0.5345	0.52850002	1.56169999	1.56270003	1.55180001	1.24520004
0.53479999	0.54589999	0.53600001	1.57389998	1.57410002	1.56449997	1.2507
0.54320002	0.55119997	0.54549998	1.58899999	1.58720005	1.57280004	1.26370001
0.54820001	0.55879998	0.5517	1.59389997	1.59290004	1.58350003	1.27359998
0.55599999	0.56639999	0.56099999	1.59650004	1.60070002	1.59319997	1.28919995
0.56660002	0.57440001	0.56400001	1.6099	1.61259997	1.60739994	1.29869998
0.57179999	0.58170003	0.5758	1.61870003	1.62160003	1.61099994	1.30929995
0.5776	0.5923	0.5819	1.62839997	1.6257	1.62059999	1.31789994
0.58710003	0.597	0.59119999	1.63409996	1.63469994	1.63170004	1.32420003
0.59390002	0.6045	0.59649998	1.64419997	1.64590001	1.63829994	1.33889997
0.59869999	0.61309999	0.60170001	1.6523	1.65180004	1.64789999	1.3441
0.60619998	0.61760002	0.61320001	1.65569997	1.65620005	1.65419996	1.35459995
0.6142	0.62730002	0.61739999	1.66170001	1.66390002	1.65760005	1.35409999

0.6196	0.63239998	0.62419999	1.67079997	1.66789997	1.66209996	1.37870002
0.6279	0.64020002	0.63429999	1.67439997	1.67620003	1.67050004	1.36699998
0.63279998	0.6455	0.64020002	1.67499995	1.67439997	1.67439997	1.37329996
0.64160001	0.65390003	0.65170002	1.68470001	1.68420005	1.6832	1.38689995
0.6469	0.66180003	0.65310001	1.68910003	1.68869996	1.68149996	1.39760005
0.6541	0.6688	0.66109997	1.69239998	1.69400001	1.68850005	1.39590001
0.66159999	0.67510003	0.67129999	1.69879997	1.69850004	1.70480001	1.41770005
0.667	0.68190002	0.67640001	1.70640004	1.70389998	1.70130002	1.40579998
0.67140001	0.68650001	0.68379998	1.70669997	1.70580006	1.70500004	1.41250002
0.68089998	0.6954	0.69389999	1.71229994	1.71389997	1.71609998	1.41999996
0.68449998	0.7026	0.69859999	1.71300006	1.71340001	1.71730006	1.42120004
0.69160002	0.70920002	0.70050001	1.72060001	1.71749997	1.7227	1.43340003
0.69880003	0.71460003	0.70740002	1.72329998	1.72370005	1.72529995	1.42820001
0.70090002	0.71850002	0.71429998	1.72889996	1.72399998	1.73160005	1.44239998
0.713	0.73040003	0.72640002	1.73029995	1.73140001	1.72660005	1.44149995
0.71719998	0.73449999	0.72710001	1.73520005	1.73860002	1.73179996	1.44819999
0.72539997	0.74430001	0.73409998	1.73670006	1.73189998	1.72950006	1.45140004
0.72930002	0.7471	0.7367	1.7392	1.73720002	1.74269998	1.45050001
0.7367	0.75489998	0.75	1.73810005	1.73749995	1.73800004	1.45500004
0.74419999	0.76139998	0.75739998	1.745	1.74039996	1.73819995	1.45420003
0.74949998	0.76660001	0.76340002	1.74520004	1.74600005	1.74619997	1.45829999
0.75470001	0.7726	0.7651	1.74749994	1.74479997	1.74409997	1.46089995
0.76010001	0.77950001	0.76929998	1.75080001	1.74899995	1.75339997	1.4691
0.76300001	0.78219998	0.77999997	1.75419998	1.7507	1.75100005	1.47449994
0.7694	0.79079998	0.77939999	1.75240004	1.75539994	1.75020003	1.47319996
0.78009999	0.79820001	0.79360002	1.75580001	1.75539994	1.75709999	1.47469997
0.78359997	0.80180001	0.7949	1.76289999	1.75940001	1.7608	1.48220003
0.78759998	0.80830002	0.80089998	1.75950003	1.76049995	1.76069999	1.48010004
0.79400003	0.81410003	0.81	1.76170003	1.76629996	1.76789999	1.47969997
0.79680002	0.8193	0.81470001	1.76719999	1.75960004	1.75339997	1.48829997
0.80330002	0.8251	0.81999999	1.76950002	1.7622	1.76069999	1.48280001
0.80970001	0.83090001	0.82279998	1.76709998	1.76540005	1.76349998	1.49070001
0.81550002	0.83490002	0.82709998	1.76660001	1.76530004	1.76559997	1.48450005
0.81849998	0.83819997	0.8319	1.77139997	1.76680005	1.77540004	1.49370003
0.82630002	0.84810001	0.84289998	1.77390003	1.76900005	1.76409996	1.49539995
0.82859999	0.8502	0.84719998	1.77129996	1.77020001	1.77380002	1.4921
0.83579999	0.85900003	0.85039997	1.77680004	1.76979995	1.77670002	1.49479997
0.83920002	0.86019999	0.85329998	1.77649999	1.77569997	1.77690005	1.50629997
0.8427	0.86799997	0.8646	1.778	1.77380002	1.76919997	1.5036
0.84859997	0.87419999	0.8664	1.77450001	1.77279997	1.77320004	1.50419998
0.85219997	0.87580001	0.87190002	1.77649999	1.7773	1.77760005	1.50919998
0.85860002	0.88300002	0.87419999	1.77999997	1.77620006	1.77199996	1.51010001
0.86400002	0.88459998	0.87840003	1.78250003	1.77670002	1.77279997	1.50269997
0.86839998	0.8915	0.88249999	1.77939999	1.77680004	1.7823	1.50730002
0.875	0.89719999	0.89120001	1.78110003	1.77939999	1.77520001	1.50779998
0.87830001	0.90270001	0.89639997	1.77989995	1.77869999	1.7766	1.50349998

0.88300002	0.90539998	0.9012	1.78659999	1.78120005	1.78400004	1.51059997
0.88529998	0.90719998	0.90759999	1.78219998	1.77939999	1.78120005	1.51890004
0.89050001	0.91820002	0.9113	1.7845	1.78460002	1.77499998	1.51269996
0.89740002	0.92129999	0.91600001	1.78629994	1.78009999	1.77390003	1.51730001
0.90009999	0.92369998	0.92220002	1.78639996	1.78569996	1.78410006	1.51440001
0.90640002	0.92809999	0.92400002	1.78340006	1.77929997	1.77880001	1.51779997
0.90960002	0.93360001	0.92379999	1.78250003	1.77839994	1.77690005	1.51310003
0.91210002	0.93790001	0.93370003	1.78250003	1.7802	1.77849996	1.51380002
0.91829997	0.9443	0.9386	1.79180002	1.78480005	1.78320003	1.51750004
0.92409998	0.94770002	0.94440001	1.78740001	1.78190005	1.78009999	1.51660001
0.92659998	0.95139998	0.94700003	1.78240001	1.78100002	1.77600002	1.52279997
0.93110001	0.9551	0.94749999	1.78610003	1.78279996	1.78059995	1.52289999
0.93559998	0.9612	0.95380002	1.78540003	1.7809	1.78120005	1.52310002
0.9375	0.96359998	0.9601	1.78840005	1.7859	1.7888	1.52180004
0.94120002	0.96740001	0.96270001	1.78729999	1.78199995	1.77690005	1.52520001
0.94499999	0.97039998	0.96509999	1.79349995	1.78279996	1.78289998	1.52919996
0.95029998	0.9745	0.96719998	1.78840005	1.78629994	1.79059994	1.5201
0.95429999	0.97750002	0.97399998	1.78709996	1.78400004	1.77880001	1.5266
0.95929998	0.98449999	0.98049998	1.79050004	1.78540003	1.79139996	1.53009999
0.96200001	0.98890001	0.98390001	1.78460002	1.78129995	1.78149998	1.52690005
0.9648	0.99059999	0.9831	1.79009998	1.78390002	1.77719998	1.5223
0.96850002	0.99190003	0.98629999	1.78849995	1.78919995	1.78929996	1.52419996
0.97320002	0.99919999	0.99150002	1.78600001	1.78129995	1.77900004	1.52929997
0.97409999	1.00119996	0.99699998	1.79009998	1.78659999	1.7895	1.52520001
0.98019999	1.00440001	0.99760002	1.78789997	1.78279996	1.77880001	1.52090001
0.98339999	1.00730002	1.00109994	1.7888	1.78369999	1.77839994	1.52520001
0.9892	1.01250005	1.00370002	1.78910005	1.78740001	1.79250002	1.52849996
0.99180001	1.01769996	1.01230001	1.78890002	1.78540003	1.77830005	1.52219999
0.9928	1.02049994	1.01629996	1.78750002	1.78830004	1.78890002	1.54299998
0.99720001	1.02550006	1.02190006	1.79100001	1.78260005	1.78419995	1.5359
0.99830002	1.02900004	1.02209997	1.78910005	1.7859	1.78789997	1.52670002
1.00580001	1.03110003	1.02779996	1.79100001	1.79040003	1.7895	1.52310002
1.00600004	1.03320003	1.02890003	1.78980005	1.78400004	1.78610003	1.52610004
1.01119995	1.03499997	1.02960002	1.78699994	1.7823	1.78750002	1.52670002
1.01590002	1.04250002	1.03820002	1.79369998	1.78410006	1.77960002	1.528
1.02049994	1.04299998	1.03789997	1.79330003	1.78670001	1.78820002	1.53260005
1.01909995	1.04809999	1.03740001	1.78919995	1.7895	1.78890002	1.52719998
1.02540004	1.05180001	1.04320002	1.79190004	1.78190005	1.77810001	1.53890002
1.02649999	1.05540001	1.04900002	1.79050004	1.78750002	1.77939999	1.52289999
1.0316	1.05980003	1.0503	1.78970003	1.78509998	1.78699994	1.53129995
1.03279996	1.06120002	1.05579996	1.79069996	1.78840005	1.78820002	1.52839994
1.03569996	1.06500006	1.05789995	1.78980005	1.78620005	1.78569996	1.52279997
1.03919995	1.06420004	1.05869997	1.79110003	1.78470004	1.78579998	1.52310002
1.04120004	1.06710005	1.06060004	1.7888	1.78269994	1.78830004	1.52980006
1.04369998	1.07159996	1.06830001	1.78919995	1.78079998	1.77320004	1.53149998
1.04779994	1.07780004	1.07149994	1.79139996	1.78439999	1.78789997	1.52250004

1.05439997	1.08130002	1.07159996	1.78559995	1.78340006	1.78219998	1.52909994
1.05540001	1.08159995	1.07490003	1.79009998	1.78419995	1.78330004	1.53069997
1.05659997	1.08510005	1.07630002	1.78980005	1.78209996	1.77380002	1.52590001
1.05770004	1.08790004	1.08399999	1.78789997	1.78340006	1.78390002	1.53330004
1.06260002	1.09280002	1.08809996	1.78719997	1.7802	1.77970004	1.52980006
1.06480002	1.09109998	1.08560002	1.78929996	1.77820003	1.77139997	1.52929997
1.07099998	1.09630001	1.08729994	1.78509998	1.78030002	1.77269995	1.53250003
1.0711	1.10010004	1.09570003	1.78699994	1.78289998	1.78330004	1.52810001
1.07389998	1.10249996	1.09710002	1.78960001	1.78569996	1.78429997	1.52970004
1.07860005	1.10710001	1.10160005	1.78960001	1.78250003	1.78199995	1.53149998
1.07879996	1.10810006	1.1027	1.78639996	1.78069997	1.77999997	1.52119994

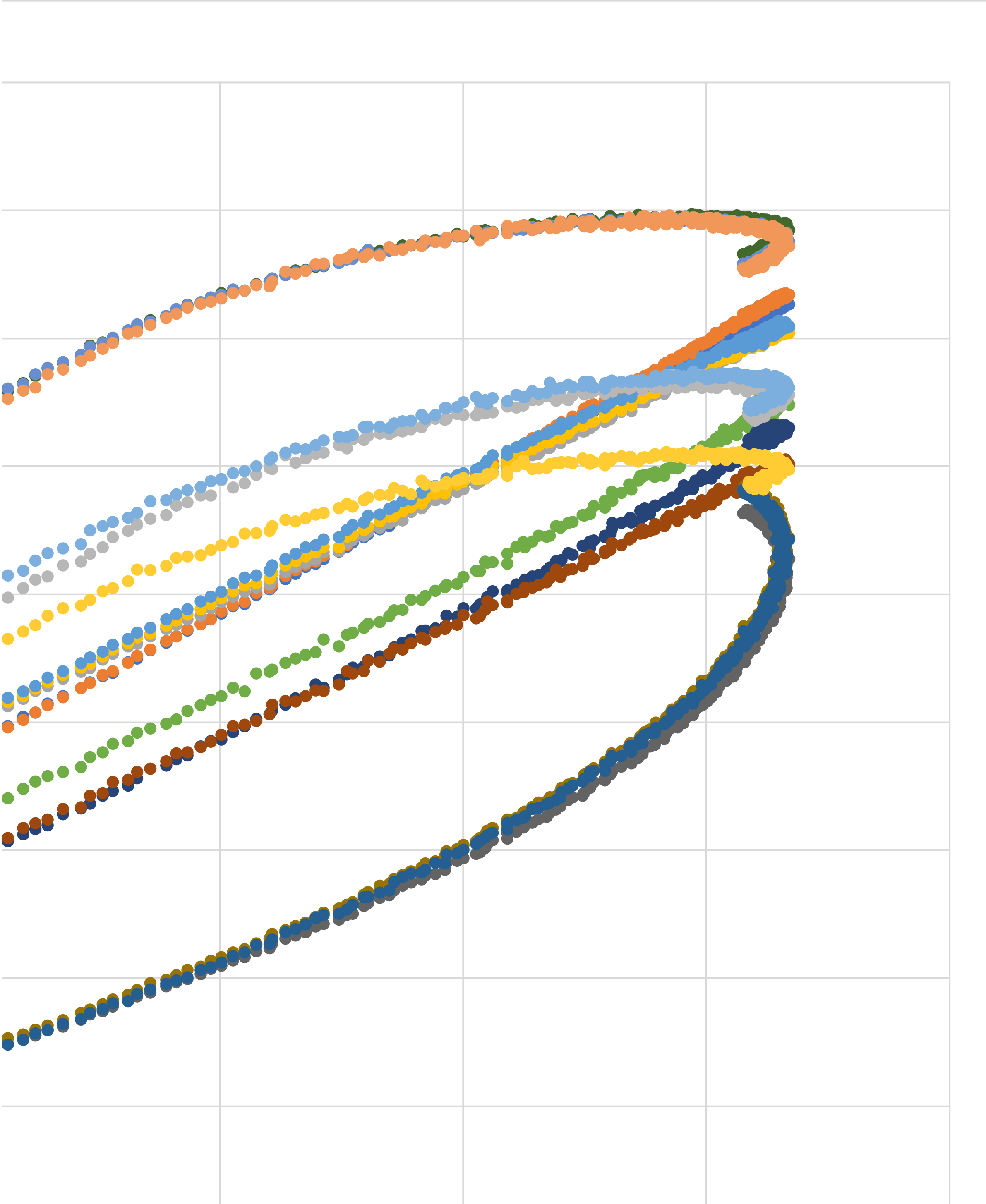

[illegible]	[illegible]	[illegible]	[illegible]	[illegible]	[illegible]	[illegible]04
[illegible]	[illegible]	[illegible]	[illegible]	[illegible]	[illegible]	[illegible]05
[illegible]	[illegible]	[illegible]	[illegible]	[illegible]	[illegible]	[illegible]96
[illegible]	[illegible]	[illegible]	[illegible]	[illegible]	[illegible]	[illegible]03
[illegible]	[illegible]	[illegible]	[illegible]	[illegible]	[illegible]	[illegible]98
[illegible]	[illegible]	[illegible]	[illegible]	[illegible]	[illegible]	[illegible]02
[illegible]	[illegible]	[illegible]	[illegible]	[illegible]	[illegible]	[illegible]05
[illegible]	[illegible]	[illegible]	[illegible]	[illegible]	[illegible]	[illegible]05
[illegible]	[illegible]	[illegible]	[illegible]	[illegible]	[illegible]	[illegible]06
[illegible]	[illegible]	[illegible]	[illegible]	[illegible]	[illegible]	[illegible]01
[illegible]	[illegible]	[illegible]	[illegible]	[illegible]	[illegible]	[illegible]02
[illegible]	[illegible]	[illegible]	[illegible]	[illegible]	[illegible]	[illegible]04
[illegible]	[illegible]	[illegible]	[illegible]	[illegible]	[illegible]	[illegible]03
[illegible]	[illegible]	[illegible]	[illegible]	[illegible]	[illegible]	[illegible]02
[illegible]	[illegible]	[illegible]	[illegible]	[illegible]	[illegible]	[illegible]73
[illegible]	[illegible]	[illegible]	[illegible]	[illegible]	[illegible]	[illegible]01
[illegible]	[illegible]	[illegible]	[illegible]	[illegible]	[illegible]	[illegible]98
[illegible]	[illegible]	[illegible]	[illegible]	[illegible]	[illegible]	[illegible]03
[illegible]	[illegible]	[illegible]	[illegible]	[illegible]	[illegible]	[illegible]96
[illegible]	[illegible]	[illegible]	[illegible]	[illegible]	[illegible]	[illegible]04
[illegible]	[illegible]	[illegible]	[illegible]	[illegible]	[illegible]	[illegible]95
[illegible]	[illegible]	[illegible]	[illegible]	[illegible]	[illegible]	[illegible]05
[illegible]	[illegible]	[illegible]	[illegible]	[illegible]	[illegible]	[illegible]95
[illegible]	[illegible]	[illegible]	[illegible]	[illegible]	[illegible]	[illegible]04
[illegible]	[illegible]	[illegible]	[illegible]	[illegible]	[illegible]	[illegible]05
[illegible]	[illegible]	[illegible]	[illegible]	[illegible]	[illegible]	[illegible]97
[illegible]	[illegible]	[illegible]	[illegible]	[illegible]	[illegible]	[illegible]97
[illegible]	[illegible]	[illegible]	[illegible]	[illegible]	[illegible]	[illegible]99
[illegible]	[illegible]	[illegible]	[illegible]	[illegible]	[illegible]	[illegible]94
[illegible]	[illegible]	[illegible]	[illegible]	[illegible]	[illegible]	[illegible]98
[illegible]	[illegible]	[illegible]	[illegible]	[illegible]	[illegible]	[illegible]97
[illegible]	[illegible]	[illegible]	[illegible]	[illegible]	[illegible]	[illegible]95
[illegible]	[illegible]	[illegible]	[illegible]	[illegible]	[illegible]	[illegible]95
[illegible]	[illegible]	[illegible]	[illegible]	[illegible]	[illegible]	[illegible]97
[illegible]	[illegible]	[illegible]	[illegible]	[illegible]	[illegible]	[illegible]98

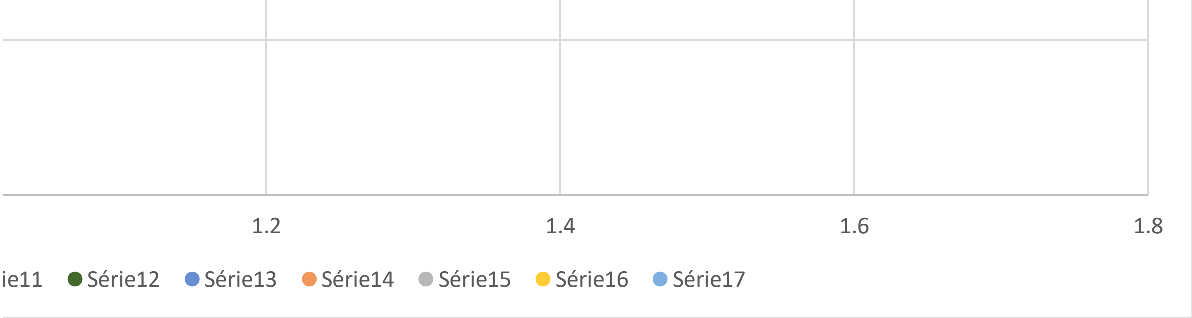

[illegible]	[illegible]	[illegible]	[illegible]	[illegible]	[illegible]	[illegible]
[illegible]	[illegible]	[illegible]	[illegible]	[illegible]	[illegible]	[illegible]
[illegible]	[illegible]	[illegible]	[illegible]	[illegible]	[illegible]	[illegible]
[illegible]	[illegible]	[illegible]	[illegible]	[illegible]	[illegible]	[illegible]
[illegible]	[illegible]	[illegible]	[illegible]	[illegible]	[illegible]	[illegible]
[illegible]	[illegible]	[illegible]	[illegible]	[illegible]	[illegible]	[illegible]
[illegible]	[illegible]	[illegible]	[illegible]	[illegible]	[illegible]	[illegible]
[illegible]	[illegible]	[illegible]	[illegible]	[illegible]	[illegible]	[illegible]
1.17569995	1.20480001	1.19739997	1.77810001	1.76839995	1.76180005	1.51909995
1.17540002	1.20290005	1.19869995	1.77950001	1.76680005	1.77219999	1.52590001
1.17630005	1.20729995	1.20159996	1.77509999	1.76979995	1.76800001	1.51470006
1.17949998	1.21329999	1.20570004	1.77939999	1.76709998	1.76680005	1.51479995
1.17690003	1.21159995	1.20589995	1.77929997	1.76689994	1.7701	1.52520001
1.1803	1.21539998	1.20739996	1.77960002	1.76839995	1.76979995	1.52579999
1.18169999	1.21200001	1.20959997	1.77919996	1.76950002	1.76530004	1.51880002
1.18079996	1.21169996	1.20700002	1.77820003	1.76660001	1.76880002	1.52310002
1.18499994	1.22049999	1.21249998	1.77620006	1.76639998	1.76689994	1.51660001
1.18889999	1.2234	1.21300006	1.78410006	1.76549995	1.75820005	1.51789999
1.18879998	1.21780002	1.21300006	1.77919996	1.76450002	1.75960004	1.51450002
1.18700004	1.22220004	1.21560001	1.77670002	1.76489997	1.75839996	1.51559997
1.19369996	1.22650003	1.21819997	1.77649999	1.76600003	1.76600003	1.51020002
1.19420004	1.22809994	1.22039998	1.77590001	1.76559997	1.7651	1.51750004
1.1925	1.22350001	1.21959996	1.78149998	1.76549995	1.75779998	1.52289999
1.19640005	1.22730005	1.22130001	1.77629995	1.7615	1.75730002	1.51590002
1.199	1.22570002	1.22029996	1.77810001	1.76259995	1.75590003	1.52100003
1.19669998	1.23140001	1.22549999	1.77690005	1.76559997	1.76300001	1.51750004
1.20430005	1.23169994	1.22580004	1.77789998	1.76999998	1.76909995	1.51629996
1.20369995	1.23029995	1.22539997	1.77750003	1.76209998	1.75639999	1.51090002
1.20439994	1.23529994	1.22780001	1.77499998	1.76139998	1.76300001	1.51699996
1.20589995	1.23899996	1.22829998	1.77890003	1.76639998	1.76440001	1.51520002
1.20510006	1.23759997	1.23080003	1.78100002	1.76460004	1.76530004	1.51919997
1.20659995	1.23790002	1.23360002	1.77540004	1.76390004	1.76339996	1.51740003
1.21309996	1.24170005	1.23450005	1.77559996	1.76349998	1.7615	1.52339995
1.21080005	1.24160004	1.23679996	1.77450001	1.76090002	1.76090002	1.51390004
1.21319997	1.24269998	1.23689997	1.77460003	1.76090002	1.75909996	1.52100003
1.20969999	1.24629998	1.23650002	1.77890003	1.76479995	1.76310003	1.51289999
1.21720004	1.24549997	1.23909998	1.77499998	1.76380002	1.755	1.51090002
1.21430004	1.24600005	1.23759997	1.77320004	1.75950003	1.75259995	1.51349998
1.21580005	1.24810004	1.24249995	1.77320004	1.76279998	1.75880003	1.51320004
1.21650004	1.25080001	1.24460006	1.77629995	1.76419997	1.76090002	1.51180005
1.21889997	1.255	1.24489999	1.77349997	1.76440001	1.76199996	1.51049995
1.22029996	1.25129998	1.24409997	1.77610004	1.76289999	1.76010001	1.50950003
1.22090006	1.25689995	1.24759996	1.77569997	1.75810003	1.76010001	1.50870001
1.2198	1.25320005	1.24979997	1.77400005	1.75670004	1.75259995	1.50699997
1.22640002	1.25610006	1.25160003	1.77349997	1.75479996	1.75	1.51020002
1.2256	1.2615	1.25269997	1.77310002	1.75909996	1.75300002	1.51689994

1.22780001	1.25829995	1.25250006	1.77429998	1.76129997	1.75569999	1.51320004
1.22749996	1.26289999	1.25300002	1.77559996	1.76030004	1.75829995	1.51440001
1.22619998	1.25960004	1.25670004	1.77170002	1.75820005	1.75639999	1.50610006
1.23290002	1.26199996	1.25839996	1.77119994	1.76119995	1.75940001	1.50240004
1.23119998	1.26049995	1.25759995	1.77499998	1.75979996	1.75600004	1.51680005
1.23259997	1.2665	1.26129997	1.77100003	1.75619996	1.75520003	1.50960004
1.23230004	1.26800001	1.26010001	1.76549995	1.75650001	1.75399995	1.52550006
1.23179996	1.26380002	1.2608	1.77020001	1.75730002	1.75349998	1.51750004
1.23619998	1.26929998	1.26279998	1.77489996	1.75610006	1.74619997	1.51129997
1.24119997	1.273	1.26429999	1.77100003	1.75569999	1.74629998	1.50969994
1.23740005	1.27190006	1.26470006	1.76950002	1.7579	1.74689996	1.50969994
1.23740005	1.27400005	1.26670003	1.76569998	1.74870002	1.75250006	1.51419997
1.24269998	1.27690005	1.26880002	1.76909995	1.75320005	1.75209999	1.50639999
1.23899996	1.27359998	1.26730001	1.77250004	1.75759995	1.75349998	1.50419998
1.24259996	1.27639997	1.26880002	1.76689994	1.75479996	1.75049996	1.50960004
1.23979998	1.27569997	1.27149999	1.76689994	1.75220001	1.74759996	1.50250006
1.24530005	1.27989995	1.27289999	1.76849997	1.75080001	1.7457	1.51440001
1.24740005	1.28139997	1.27550006	1.77040005	1.75520003	1.74679995	1.50849998
1.24779999	1.28349996	1.27390003	1.77030003	1.75539994	1.74580002	1.5086
1.24810004	1.28079998	1.27579999	1.76810002	1.75220001	1.75100005	1.50999999
1.25139999	1.27980006	1.27600002	1.76929998	1.75090003	1.74349999	1.50759995
1.24860001	1.28419995	1.27760005	1.76629996	1.74979997	1.74969995	1.50489998
1.25080001	1.28540003	1.27620006	1.76740003	1.75460005	1.74899995	1.50370002
1.24759996	1.28240001	1.27989995	1.76250005	1.75150001	1.75030005	1.51119995
1.25310004	1.28639996	1.28199995	1.76549995	1.75119996	1.74870002	1.51010001
1.2529	1.2888	1.2802	1.76409996	1.74860001	1.74800003	1.50469995
1.25660002	1.2895	1.28320003	1.76989996	1.75590003	1.74849999	1.50680006
1.25479996	1.2881	1.28540003	1.76890004	1.75080001	1.74419999	1.51129997
1.25329995	1.28960001	1.28170002	1.76359999	1.74559999	1.74389994	1.5072
1.25919998	1.29359996	1.28489995	1.76489997	1.74960005	1.74590003	1.50699997
1.25510001	1.28939998	1.28600001	1.76320004	1.74829996	1.74339998	1.50569999
1.25940001	1.29340005	1.29059994	1.76020002	1.74720001	1.74559999	1.50610006
1.26230001	1.29190004	1.29050004	1.76839995	1.75199997	1.74399996	1.50779998
1.2608	1.29620004	1.28699994	1.76730001	1.75100005	1.74849999	1.50870001
1.26349998	1.29990005	1.28840005	1.76419997	1.74890006	1.7414	1.50259995
1.26269996	1.30069995	1.29219997	1.76600003	1.74950004	1.74619997	1.50999999
1.26110005	1.29830003	1.29079998	1.76479995	1.74440002	1.74059999	1.50689995
1.26339996	1.2974	1.29400003	1.76160002	1.74740005	1.74059999	1.50580001
1.26810002	1.29820001	1.29509997	1.76520002	1.74510002	1.73720002	1.50269997
1.26250005	1.29929996	1.29569995	1.75940001	1.74629998	1.73880005	1.50530005
1.2651	1.30200005	1.29779994	1.76629996	1.75109994	1.7471	1.50240004
1.26820004	1.30149996	1.29209995	1.75919998	1.74440002	1.7385	1.50209999
1.26900005	1.30420005	1.2974	1.76450002	1.74679995	1.73979998	1.50849998
1.26979995	1.30579996	1.3003	1.76339996	1.74720001	1.7421	1.50440001
1.27269995	1.30589998	1.30200005	1.75759995	1.74790001	1.7457	1.49989998
1.27090001	1.30869997	1.30079997	1.75979996	1.7464	1.74689996	1.50160003

1.26909995	1.30869997	1.3017	1.7586	1.74370003	1.74790001	1.50240004
1.2766	1.30920005	1.30400002	1.76520002	1.74759996	1.74349999	1.50240004
1.27429998	1.30879998	1.3046	1.75950003	1.74549997	1.74539995	1.50639999
1.27559996	1.31070006	1.30630004	1.76259995	1.74730003	1.74220002	1.50329995
1.27670002	1.30949998	1.30680001	1.7608	1.74779999	1.74220002	1.50390005
1.27419996	1.31140006	1.30219996	1.7543	1.74160004	1.74070001	1.5
1.27869999	1.3154	1.30680001	1.76069999	1.74600005	1.73959994	1.50619996
1.27859998	1.3089	1.30830002	1.76209998	1.7457	1.74160004	1.49430001
1.278	1.31130004	1.31029999	1.76010001	1.74510002	1.74430001	1.50230002
1.28359997	1.31760001	1.30719995	1.7572	1.74160004	1.74150002	1.49989998
1.27779996	1.31400001	1.30509996	1.75530005	1.74150002	1.74179995	1.49769998
1.2809	1.31700003	1.3089	1.75660002	1.74109995	1.73510003	1.50590003
1.28400004	1.32019997	1.30809999	1.75909996	1.74469995	1.73889995	1.49769998
1.28260005	1.3175	1.30900002	1.75419998	1.74300003	1.73810005	1.50109994
1.28100002	1.3175	1.30900002	1.7536	1.7385	1.73930001	1.49590003
1.28610003	1.32190001	1.31089997	1.75769997	1.7464	1.74170005	1.49960005
1.28359997	1.31860006	1.31659997	1.75769997	1.74030006	1.73269999	1.50100005
1.28659999	1.32389998	1.31570005	1.75399995	1.74000001	1.74090004	1.50179994
1.28579998	1.32009995	1.32019997	1.75619996	1.73899996	1.73599994	1.49779999
1.28400004	1.32249999	1.3132	1.75779998	1.74469995	1.73839998	1.49450004
1.28359997	1.32249999	1.31480002	1.75740004	1.74199998	1.73230004	1.49460006
1.28849995	1.32219994	1.3175	1.75230002	1.73539996	1.73020005	1.50090003
1.28670001	1.32120001	1.31869996	1.75580001	1.73899996	1.73619998	1.50240004
1.2881	1.32519996	1.31669998	1.75689995	1.74399996	1.7385	1.49880004
1.28960001	1.32620001	1.3161	1.75639999	1.74059999	1.72959995	1.49549997
1.28789997	1.32539999	1.31579995	1.75380003	1.73749995	1.73500001	1.49820006
1.29170001	1.32630002	1.32190001	1.75080001	1.73280001	1.72710001	1.49790001
1.29209995	1.32550001	1.32379997	1.75520003	1.73930001	1.7306	1.50090003
1.29470003	1.33060002	1.32060003	1.75849998	1.73710001	1.73580003	1.49339998
1.29219997	1.33029997	1.32319999	1.75100005	1.7392	1.73469996	1.49409997
1.29560006	1.32990003	1.32930005	1.75539994	1.73790002	1.73099995	1.49559999
1.29270005	1.33089995	1.32539999	1.75419998	1.73810005	1.73479998	1.49460006
1.29550004	1.32930005	1.32679999	1.75259995	1.73380005	1.72850001	1.49720001
1.29709995	1.33440006	1.32490003	1.75250006	1.73689997	1.72950006	1.49919999
1.29400003	1.33169997	1.324	1.75230002	1.73650002	1.7342	1.49290001
1.29630005	1.33389997	1.32879996	1.75240004	1.73370004	1.72780001	1.49039996
1.29859996	1.33399999	1.32249999	1.75150001	1.73759997	1.7313	1.49549997
1.29620004	1.33319998	1.32509995	1.75109994	1.73720002	1.73199999	1.49290001
1.29680002	1.33369994	1.32860005	1.74660003	1.73239994	1.73010004	1.49389994
1.29840004	1.33389997	1.33130002	1.75259995	1.73500001	1.73409998	1.49090004
1.30219996	1.33899999	1.32739997	1.75310004	1.73500001	1.73210001	1.49319994
1.3017	1.33940005	1.32739997	1.75170004	1.7335	1.72710001	1.48839998
1.29929996	1.33710003	1.32969999	1.74730003	1.73150003	1.72430003	1.48979998
1.29859996	1.33529997	1.33399999	1.7464	1.73430002	1.72510004	1.49720001
1.30369997	1.33879995	1.33630002	1.74660003	1.73080003	1.72749996	1.48889995
1.30040002	1.33879995	1.32939994	1.74989998	1.73239994	1.73080003	1.49049997

1.30120003	1.34210002	1.33159995	1.7493	1.73370004	1.73220003	1.4914
1.30490005	1.34259999	1.33109999	1.75129998	1.7342	1.72819996	1.49310005
1.30369997	1.33840001	1.33329999	1.74660003	1.73090005	1.72880006	1.49199998
1.30420005	1.34179997	1.32949996	1.7464	1.73020005	1.72979999	1.49670005
1.30490005	1.34150004	1.33700001	1.7507	1.7349	1.72899997	1.49409997
1.30149996	1.33850002	1.33759999	1.75250006	1.7335	1.72580004	1.49860001
1.30690002	1.34300005	1.33889997	1.74580002	1.73070002	1.72230005	1.4921
1.30799997	1.34500003	1.33459997	1.74590003	1.73109996	1.72399998	1.48510003
1.30350006	1.34379995	1.33299994	1.74619997	1.72829998	1.722	1.49800003
1.30780005	1.34469998	1.33729994	1.74759996	1.72940004	1.72300005	1.4849
1.30799997	1.34580004	1.33630002	1.74870002	1.73380005	1.72350001	1.48689997
1.30999994	1.34560001	1.34010005	1.74580002	1.72909999	1.72399998	1.49339998
1.3082	1.34360003	1.33500004	1.74409997	1.7299	1.71930003	1.49070001
1.30879998	1.34870005	1.34399998	1.74530005	1.72829998	1.71969998	1.48829997
1.31009996	1.34669995	1.34130001	1.7457	1.72500002	1.72140002	1.48590004
1.31060004	1.34710002	1.33850002	1.74440002	1.72669995	1.72379994	1.49609995
1.30949998	1.34689999	1.33759999	1.74319994	1.72819996	1.72070003	1.48860002
1.30990005	1.34679997	1.3398	1.74419999	1.72920001	1.71850002	1.48769999
1.31369996	1.34860003	1.34379995	1.74619997	1.72899997	1.72160006	1.48930001
1.30939996	1.34720004	1.34399998	1.74319994	1.72689998	1.72130001	1.47899997
1.31229997	1.35179996	1.34070003	1.74059999	1.727	1.71990001	1.48520005
1.31040001	1.34979999	1.34099996	1.74469995	1.727	1.72080004	1.4777
1.31260002	1.35239995	1.34080005	1.74430001	1.72529995	1.72309995	1.48730004
1.3175	1.35290003	1.34909999	1.74290001	1.72290003	1.71860003	1.48160005
1.31299996	1.34959996	1.35020006	1.73959994	1.72640002	1.72370005	1.48389995
1.3161	1.35640001	1.34309995	1.74160004	1.72479999	1.722	1.48839998
1.31599998	1.35570002	1.34430003	1.74629998	1.72689998	1.72179997	1.47759998
1.31570005	1.35230005	1.34739995	1.74389994	1.72399998	1.72189999	1.48109996
1.31669998	1.35300004	1.34469998	1.73880005	1.72230005	1.7227	1.48570001
1.31719995	1.35370004	1.35070002	1.73969996	1.72259998	1.71959996	1.48269999
1.31990004	1.35819995	1.34420002	1.74090004	1.72230005	1.71800005	1.47860003
1.31879997	1.35549998	1.34500003	1.74510002	1.72350001	1.72150004	1.48670006
1.31780005	1.35899997	1.34850001	1.7378	1.72500002	1.72230005	1.48290002
1.32070005	1.35539997	1.34609997	1.74310005	1.72459996	1.71940005	1.47950006
1.31690001	1.35609996	1.34730005	1.73469996	1.72109997	1.71710002	1.48520005
1.32009995	1.35780001	1.35329998	1.73740005	1.722	1.72000003	1.48249996
1.32019997	1.36049998	1.34819996	1.74460006	1.72580004	1.71879995	1.48099995
1.31819999	1.35590005	1.35389996	1.73689997	1.72379994	1.71940005	1.48249996
1.31819999	1.35800004	1.34749997	1.73930001	1.7256	1.72090006	1.48360002
1.3175	1.35940003	1.35669994	1.73469996	1.71580005	1.71730006	1.48169994
1.3197	1.35730004	1.35459995	1.73280001	1.71689999	1.71539998	1.48280001
1.32140005	1.36210001	1.35099995	1.74160004	1.72710001	1.71490002	1.47979999
1.31980002	1.36000001	1.34889996	1.73660004	1.71889997	1.71039999	1.48559999
1.32070005	1.3592	1.35179996	1.73609996	1.72399998	1.71519995	1.48300004
1.32149994	1.35969996	1.35679996	1.73730004	1.71720004	1.71669996	1.47469997
1.31860006	1.35839999	1.3563	1.73300004	1.71510005	1.71599996	1.4806

1.32519996	1.36590004	1.35309994	1.73619998	1.72230005	1.71280003	1.48070002
1.32369995	1.36399996	1.35329998	1.73880005	1.72109997	1.71200001	1.47969997
1.32439995	1.36179996	1.35000002	1.73360002	1.71759999	1.71010005	1.47979999
1.32219994	1.36119998	1.35000002	1.73479998	1.71949995	1.71720004	1.48150003
1.32239997	1.3642	1.35370004	1.73679996	1.71580005	1.71270001	1.48339999
1.32519996	1.36339998	1.35389996	1.73529994	1.72070003	1.71679997	1.48049998
1.32459998	1.36230004	1.35360003	1.73409998	1.71809995	1.71309996	1.48220003
1.32679999	1.36479998	1.35430002	1.72780001	1.71739995	1.71200001	1.48119998
1.32599998	1.36629999	1.35420001	1.73360002	1.71749997	1.71019995	1.4878
1.32609999	1.3642	1.36389995	1.73189998	1.71360004	1.71249998	1.47090006
1.32640004	1.3678	1.36259997	1.7335	1.7191	1.71169996	1.48329997
1.32260001	1.36409998	1.35350001	1.73300004	1.71490002	1.71500003	1.47599995
1.32930005	1.36570001	1.35640001	1.7313	1.71710002	1.70930004	1.48090005
1.32599998	1.36570001	1.35570002	1.73189998	1.71580005	1.70599997	1.48049998
1.32910001	1.36749995	1.36389995	1.73259997	1.7148	1.71140003	1.48319995
1.32669997	1.36380005	1.36380005	1.73169994	1.71659994	1.70920002	
1.32739997	1.36699998	1.36290002	1.73559999	1.71459997	1.71140003	
1.33159995	1.36759996	1.35720003	1.73039997	1.71309996	1.70879996	
1.32850003	1.3671	1.35520005	1.73049998	1.71210003	1.71029997	
1.32920003	1.37279999	1.3592	1.73339999	1.71630001	1.71060002	
1.32790005	1.36899996	1.3642	1.72889996	1.7105	1.70640004	
1.32459998	1.36549997	1.36479998	1.72930002	1.71239996	1.70799994	
1.33070004	1.37100005	1.3599	1.72880006	1.71220005	1.70480001	
1.3283	1.36870003	1.35769999	1.72930002	1.71140003	1.70539999	
1.3319	1.36950004	1.36619997	1.73099995	1.71140003	1.70640004	
1.32720006	1.36730003	1.3664				

3	
0.1876	0.2053
0.1813	0.2095
0.1864	0.2164
0.1904	0.21709999
0.18529999	0.2093
0.1821	0.21780001
0.184	0.2262
0.20110001	0.2131
0.1842	0.20559999
0.18359999	0.227
0.18189999	0.2212
0.183	0.2119
0.18179999	0.21269999
0.1824	0.20389999
0.184	0.2051
0.1789	0.2076
0.18619999	0.2164
0.18780001	0.2172
0.18889999	0.20469999
0.186	0.2097
0.1831	0.2077
0.1815	0.2304
0.1917	0.22840001
0.19329999	0.24240001
0.1816	0.21250001
0.20020001	0.2106
0.1859	0.22409999
0.20190001	0.2383
0.1964	0.2236
0.19840001	0.219
0.1825	0.21179999
0.1793	0.2089
0.19329999	0.2052
0.1869	0.21690001
0.26199999	0.32800001
0.2958	0.36359999
0.32350001	0.3845
0.35839999	0.41389999
0.3863	0.44909999
0.41819999	0.48769999
0.4339	0.51679999
0.4725	0.54680002
0.50230002	0.58170003
0.5302	0.6063

0.55830002	0.63819999
0.58359998	0.67970002
0.61180001	0.70499998
0.62779999	0.7439
0.6548	0.76740003
0.68480003	0.78289998
0.70910001	0.80680001
0.72979999	0.83230001
0.75620002	0.85799998
0.78039998	0.88870001
0.80220002	0.90350002
0.82249999	0.92460001
0.8495	0.94669998
0.8732	0.96560001
0.88730001	0.98549998
0.90310001	1.00740004
0.9375	1.02740002
0.93879998	1.05350006
0.96439999	1.06400001
0.97839999	1.08099997
0.99239999	1.10049999
1.01100004	1.11549997
1.02690005	1.13919997
1.04960001	1.16260004
1.06149995	1.16240001
1.07009995	1.17799997
1.08850002	1.19529998
1.09930003	1.2062
1.11199999	1.21200001
1.13030005	1.22969997
1.1415	1.23679996
1.15149999	1.25279999
1.16680002	1.26400006
1.17789996	1.27180004
1.1825	1.27810001
1.19149995	1.29949999
1.20469999	1.30639994
1.20969999	1.31299996
1.22000003	1.31980002
1.2385	1.32739997
1.23740005	1.34570003
1.24419999	1.34710002
1.25689995	1.35590005
1.25899994	1.36269999
1.26129997	1.3678
1.2694	1.37650001

1.27639997	1.38049996
1.28180003	1.38890004
1.29470003	1.39219999
1.296	1.40030003
1.29890001	1.40999997
1.30680001	1.41400003
1.31649995	1.42159998
1.31369996	1.42840004
1.31889999	1.42639995
1.32490003	1.43299997
1.32679999	1.4404
1.33420002	1.4461
1.34099996	1.44570005
1.33679998	1.44860005
1.34679997	1.46099997
1.3513	1.46200001
1.35539997	1.46200001
1.35520005	1.45920002
1.36629999	1.46700001
1.36170006	1.47070003
1.35650003	1.47130001
1.37779999	1.48020005
1.3721	1.47430003
1.36819994	1.48010004
1.37150002	1.49109995
1.37549996	1.49129999
1.37750006	1.49290001
1.38170004	1.5
1.38030005	1.50800002
1.38039994	1.50059998
1.38100004	1.50329995
1.38759995	1.49769998
1.38800001	1.50650001
1.38530004	1.50199997
1.39129996	1.50170004
1.40009999	1.51139998
1.40929997	1.50810003
1.40310001	1.50870001
1.39579999	1.51730001
1.39649999	1.51300001
1.39789999	1.51499999
1.40330005	1.53079998
1.40600002	1.52199996
1.4052	1.52279997
1.40670002	1.52209997
1.40670002	1.52709997

1.40470004	1.5244
1.41260004	1.52559996
1.40869999	1.53250003
1.40429997	1.52509999
1.40970004	1.53170002
1.4066	1.52330005
1.41279995	1.52970004
1.40199995	1.52190006
1.40849996	1.5323
1.41219997	1.53190005
1.41240001	1.53419995
1.41610003	1.53269994
1.41110003	1.53439999
1.41100001	1.53250003
1.41499996	1.53129995
1.40719998	1.53429997
1.41349995	1.53489995
1.41040003	1.53149998
1.41429996	1.53299999
1.41349995	1.53170002
1.41859996	1.53540003
1.41890001	1.54190004
1.41620004	1.53429997
1.41939998	1.53369999
1.41649997	1.5345
1.42270005	1.54349995
1.41589999	1.54170001
1.4152	1.54260004
1.41999996	1.53750002
1.41999996	1.53659999
1.42060006	1.53299999
1.41340005	1.5359
1.41610003	1.54089999
1.41069996	1.54709995
1.41470003	1.53919995
1.41670001	1.54299998
1.41789997	1.54809999
1.42460001	1.54110003
1.41600001	1.54270005
1.41849995	1.53900003
1.421	1.53610003
1.41830003	1.54100001
1.41719997	1.53799999
1.41949999	1.5438
1.41779995	1.54359996
1.41690004	1.54289997

1.41869998	1.54040003
1.4109	1.53970003
1.41579998	1.53849995
1.42159998	1.54530001
1.41540003	1.54279995
1.4095	1.54410005
1.41460001	1.53799999
1.42159998	1.54110003
1.42009997	1.54480004
1.41460001	1.54509997
1.41390002	1.54159999
1.42079997	1.54540002
1.41410005	1.54579997
1.4131	1.54410005
1.41980004	1.54200006
1.41260004	1.5388
1.40929997	1.54299998
1.41900003	1.5431
1.41340005	1.53989995
1.42209995	1.53750002
1.41579998	1.53890002
1.41690004	1.5345
1.41330004	1.54299998
1.41600001	1.54120004
1.4181	1.53250003
1.41009998	1.53849995
1.4188	1.54130006
1.41419995	1.54190004
1.41429996	1.54180002
1.41620004	1.5402
1.41910005	1.53939998
1.41120005	1.53540003
1.41219997	1.53120005
1.41750002	1.54180002
1.41750002	1.54159999
1.4145	1.5402
1.41540003	1.53830004
1.41680002	1.53659999
1.41550004	1.5316
1.41240001	1.53530002
1.41100001	1.53129995
1.41729999	1.53719997
1.4102	1.53569996
1.40919995	1.5359
1.41680002	1.54359996
1.40649998	1.53269994

1.40919995	1.53260005
1.40929997	1.53489995
1.40649998	1.5352
1.41499996	1.53279996
1.4109	1.53400004
1.40840006	1.53699994
1.40939999	1.53400004
1.41219997	1.53509998
1.41260004	1.5316
1.40989995	1.53209996
1.40719998	1.52750003
1.40810001	1.53840005
1.40799999	1.53779995
1.40960002	1.53349996
1.40779996	1.53760004
1.40499997	1.53840005
1.40859997	1.53310001
1.41279995	1.54120004
1.40810001	1.53209996
1.40929997	1.53380001
1.40400004	1.52579999
1.4059	1.53699994
1.4066	1.53770006
1.41320002	1.53699994
1.40970004	1.53129995
1.40639997	1.53540003
1.40279996	1.5273
1.41129994	1.53400004
1.40910006	1.5237
1.40890002	1.52670002
1.4059	1.53349996
1.40929997	1.52839994
1.40219998	1.528
1.40709996	1.52590001
1.40540004	1.52919996
1.40139997	1.52390003
1.4023	1.5187
1.40359998	1.51979995
1.4016	1.52180004
1.40250003	1.52590001
1.3944	1.52719998
1.40769994	1.52260005
1.40219998	1.52649999
1.40310001	1.52559996
1.40240002	1.5244
1.40480006	1.52890003

1.40610003	1.52359998
1.39670002	1.52960002
1.40180004	1.52450001
1.40330005	1.52470005
1.40400004	1.52310002
1.39699996	1.52919996
1.39820004	1.52380002
1.40219998	1.53219998
1.39820004	1.52040005
1.40209997	1.52100003
1.39639997	1.52059996
1.39979994	1.52030003
1.39989996	1.52170002
1.39489996	1.5187
1.39549994	1.5244
1.3987	1.52269995
1.39690006	1.52600002
1.39970005	1.52400005
1.40100002	1.52279997
1.39999998	1.52310002
1.39890003	1.51619995
1.39810002	1.52320004
1.39680004	1.53009999
1.39989996	1.52359998
1.39989996	1.51540005
1.39639997	1.51689994
1.40069997	1.52250004
1.39460003	1.52240002
1.39180005	1.52320004
1.39540005	1.51499999
1.39189994	1.52380002
1.39199996	1.51719999
1.39579999	1.51699996
1.39540005	1.51370001
1.39219999	1.51629996
1.39219999	1.51390004
1.39090002	1.51370001
1.39230001	1.51760006
1.38849998	1.51090002
1.39590001	1.51289999
1.39209998	1.51689994
1.39310002	1.51750004
1.39660001	1.51310003
1.39260006	1.51450002
1.39260006	1.51240003
1.39129996	1.51559997

1.39030004	1.51320004
1.38880002	1.51559997
1.39569998	1.51810002
1.39199996	1.51349998
1.38960004	1.51129997
1.39030004	1.50660002
1.3951	1.50769997
1.38890004	1.51320004
1.39240003	1.50709999
1.38810003	1.50600004
1.38929999	1.51450002
1.39579999	1.51440001
1.39250004	1.51100004
1.39030004	1.51689994
1.38730001	1.50639999
1.39419997	1.51170003
1.38900006	1.51139998
1.39049995	1.51489997
1.38859999	1.50820005
1.38880002	1.50409997
1.38800001	1.51409996
1.39110005	1.50800002
1.38960004	1.50810003
1.38870001	1.50940001
1.39160001	1.51259995
1.38429999	1.50899994
1.38020003	1.5079
1.38590002	1.50779998
1.38530004	1.50870001
1.38610005	1.51300001
1.38730001	1.50569999
1.38569999	1.50600004
1.38820004	1.50680006
1.3872	1.50769997
1.38339996	1.50269997
1.38530004	1.51470006
1.38259995	1.50810003
1.38510001	1.5036
1.3858	1.51230001
1.38380003	1.51590002
1.38829994	1.51020002
1.3858	1.50020003
1.38259995	1.50160003
1.38329995	1.5043
1.38520002	1.50870001
1.3872	1.50530005

1.38310003	1.50370002
1.38110006	1.49950004
1.38460004	1.5043
1.38370001	1.50310004
1.3836	1.50880003
1.38440001	1.50020003
1.38180006	1.50759995
1.38110006	1.49979997
1.38399994	1.50370002
1.38	1.50119996
1.38199997	1.5
1.38160002	1.50209999
1.38469994	1.50039995
1.37769997	1.50460005
1.38170004	1.50549996
1.38199997	1.5043
1.37880003	1.50409997
1.37709999	1.49590003
1.38670003	1.50049996
1.37800002	1.49989998
1.37750006	1.49860001
1.37960005	1.50650001
1.37160003	1.49670005
1.38250005	1.49749994
1.38010001	1.49319994
1.37940001	1.49699998
1.37479997	1.50090003
1.37660003	1.50349998
1.37720001	1.49660003
1.37730002	1.49699998
1.3779	1.50370002
1.37740004	1.4993
1.37660003	1.49329996
1.37479997	1.49699998
1.37839997	1.5007
1.37590003	1.49539995
1.3786	1.50329995
1.37300003	1.49419999
1.37150002	1.5
1.37549996	1.5043
1.36889994	1.49759996
1.37650001	1.50030005
1.37619996	1.49530005
1.37520003	1.49749994
1.36950004	1.49530005
1.37020004	1.49430001

1.37150002	1.49520004
1.36389995	1.4921
1.37119997	1.49430001
1.37329996	1.49329996
1.37220001	1.50059998
1.36880004	1.495
1.37670004	1.49769998
1.36730003	1.49940002
1.3721	1.48909998
1.37129998	1.4957
1.37580001	1.49479997
1.36609995	1.48689997
1.37559998	1.49319994
1.37109995	1.48989999
1.36430001	1.48749995
